# Enhanced crystal-melt segregation within dynamic mush systems links silicic cumulates with caldera-forming eruptions

**DOI:** 10.1038/s41467-026-74812-4

**Published:** 2026-06-27

**Authors:** Lucas M. Lino, Francy R. Quiroz-Valle, Silvio R. F. Vlach, Olivier Bachmann, Dougal A. Jerram, Valdecir de A. Janasi, Mathias Hueck, Antomat Avelino Macêdo Filho, Miguel Â. S. Basei

**Affiliations:** 1 Instituto de Geociências, Universidade de São Paulo – USP, São Paulo, Brasil; 2 Department of Earth and Planetary Sciences, ETH Zurich, Zürich, Switzerland; 3 NJORD Centre, Department of Geosciences, University of Oslo, PO Box 1028 Blindern, Oslo, Norway; 4 DougalEARTH Ltd., 31 Whitefields Crescent, Solihull, UK; 5 Institute of Geosciences, Ruhr-Universität, Bochum, Germany

**Keywords:** Petrology, Volcanology, Mineralogy

## Abstract

Supported by the mush model, the origin of crystal-poor, high-silica rhyolitic magmas (≥ 75 SiO₂ wt.%) is commonly linked to melt extraction in shallow, crystalline mushes, yet their complementary cumulates remain elusive in the upper crust. Here we present the “dynamic mush model” for the Campo Alegre-Corupá system (Brazil), combining textural analysis and thermodynamic modeling to show that high-silica melts formed through upper-crustal fractionation, leaving a granitic residue and feeding a caldera-forming eruption. Syenites and melasyenites represent their silicic-mafic cumulates, the syenites being formed at relatively low crystallinities (~16-33 of bulk vol.%) after extraction of large amounts of interstitial melts (~47-80 of liquid vol.%). In contrast to static models (extraction window ~50-70 vol.%), our results indicate early melt segregation in a dynamic reservoir. Alkali-feldspar crystals were size-selectively and hydraulically sorted by upward melt flow; aggregates were then formed at low crystallinities, supposedly via synneusis, enabling rapid sinking, and were repacked by recharge. This process, enhanced by relatively high magma fluxes, efficiently separated crystals from melt, explaining the origin of large-volume eruptible magmas in the upper crust. Our findings redefine mush evolution while underscoring the role of flow-driven crystal sorting (elutriation) in caldera-forming systems.

## Abstract (web summary)

In this study, the authors show that silicic magma reservoirs are dynamic systems that efficiently separate crystals from melt, generating high quantities of eruptible magma for large caldera-forming eruptions that leave behind crystal-rich rocks.

## Introduction

The relationship between silicic volcanic rocks and shallow plutonic systems from a trans-crustal perspective remains a central topic in igneous petrology^1–16^. The mush model, which describes magma reservoirs not as fully molten bodies, but as crystal-rich (~35–70 vol.%), partially molten systems, has been widely used to explore their shared petrogenetic evolution^1–9^. However, linking volcanic and plutonic domains remains problematic because plutonic rocks rarely preserve clear compositional or textural evidence of cumulate formation^10–12^, as they commonly represent frozen mixtures of crystals and variable proportions of trapped residual melts^13^. Conversely, although volcanic rocks may reflect the end products of crystal-melt segregation, they can show ambiguous petrogenetic signatures, combining fractional crystallization or extraction from and/or remelting of silicic mushes, complicating geochemical modeling^7,14^.

Although challenging, understanding the formation of crystal-poor, eruptible magma in the upper crust is critical as it drives large silicic eruptions^15,16^. Silicic cumulates often record variable melt-extraction efficiencies and magma mixing, hindering their geochemical distinction from rocks along the same liquid line of descent (LLD)^7,13^. Additionally, textural modifications are difficult to track owing to the limited availability of qualitative and quantitative approaches^17^. While crystal-liquid segregation is well-documented in mafic, sometimes near-monomineralic cumulates, unequivocal evidence in silicic systems remains less common^18–20^. Most silicic cumulates are identified geochemically^6,9,21^, with fewer textural examples^22–24^, mainly because they are polymineralic. Advances in detailed petrographic analysis, especially when integrated with compositional data^25,26^, offer new opportunities to resolve the evolution of complex magma/crystal systems. Applying these approaches to silicic systems, while explicitly considering processes such as crystal-melt segregation, clustering and attachment via synneusis, and framework development, provides a pathway to link crystal mushes to eruptive products.

Here, we investigate the Neoproterozoic Campo Alegre-Corupá complex (Supplementary Fig. 1), recently recognized as a well-exposed volcanic-plutonic system in which syenites represent the crystal-rich residue of caldera-forming eruption(s)^9,27,28^. Using integrated textural and compositional data, we evaluate crystal-melt segregation processes. Textural evidence indicates that alkali-feldspar glomerocrysts form the fundamental building blocks of the silicic cumulates through mutual attachment, supposedly via synneusis^29^, and/or reworking/remobilization of pre-existing, low-volume touching frameworks^30^. These features point to a dynamic, melt-rich system characterized by physical mobility, likely fueled by repeated magma recharge. Our findings suggest that melt extraction began at lower crystallinities than predicted by the classical mush model, resulting in higher melt extraction efficiency than initially computed. If broadly applicable to silicic systems, such as those found beneath the Taupō, Yellowstone, and Campi Flegrei calderas, this mechanism could help reconcile the apparent discrepancy between crystal-poor silicic eruptive products and the supposed absence of silicic cumulates in many caldera-forming systems worldwide.

## Results and discussion

### Qualitative textural and compositional characterization

**Fig. 1:**
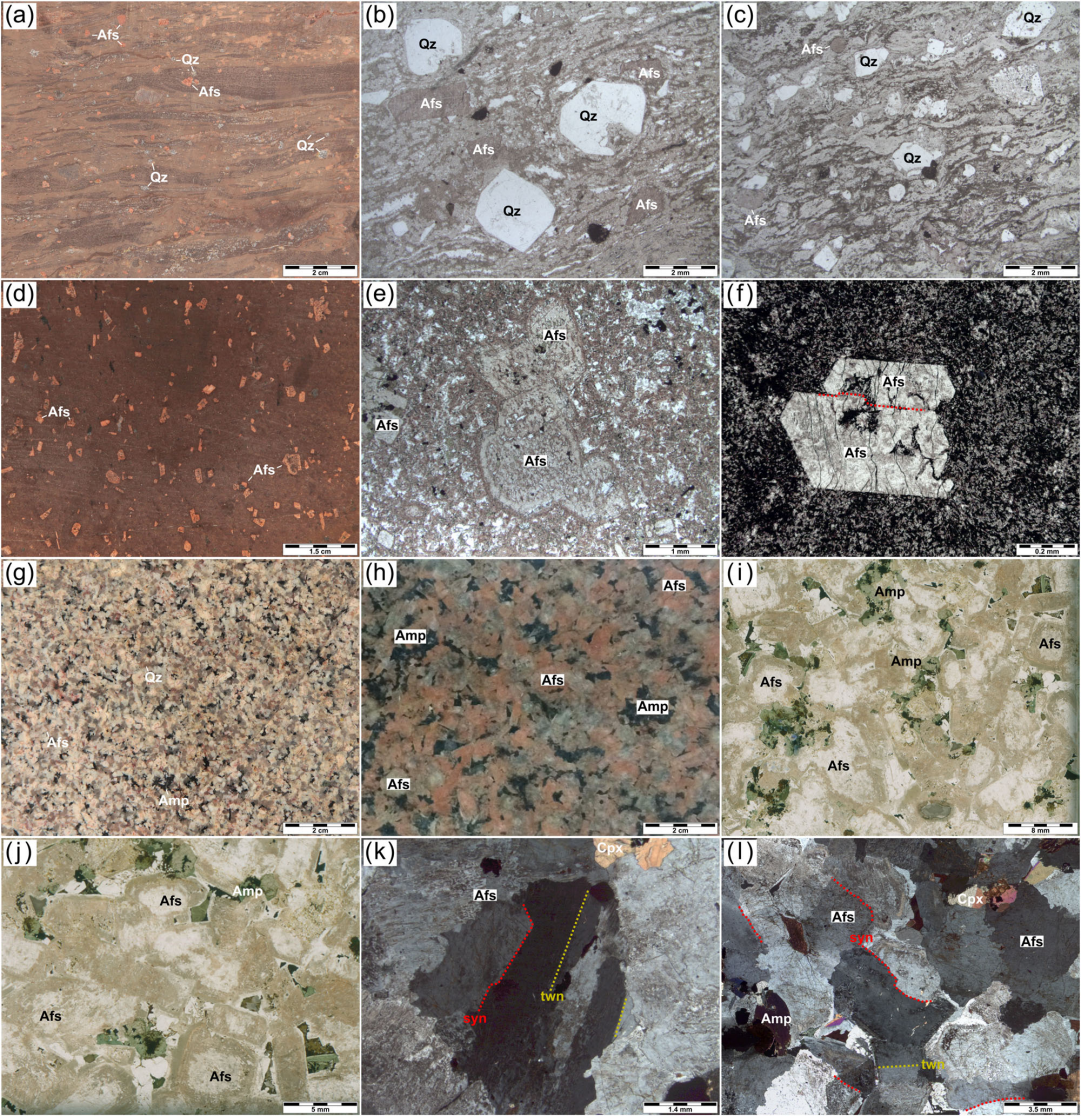
Representative rock slabs and thin sections from volcanic and plutonic rocks of the Campo Alegre-Corupá Volcanic and Igneous Plumbing System (CAC-VIPS). **a** Macroscopic and **b**,**c** microscopic textural aspects of high-silica ignimbrites from the caldera-forming eruption(s). Note the occurrence of well-formed and frequently fragmented alkali-feldspar (Afs) and quartz (Qz) crystals representing a relatively small volume of the bulk rock. Their main constituents are juvenile fragments, including lapilli-sized fiamme immersed in a devitrified ash matrix. **d** Macroscopic and **e**,**f** microscopic textural aspects of porphyritic trachytes from early- to late-stage effusive eruptions relative to the caldera-forming phase. The main mineral phase is Afs, occurring as phenocrysts and representing the major constituent of the matrix. Note the formation of crystal agglomerates and crystal pairs supposedly formed by synneusis. **g** Representative rock slab of the medium-grained, high-silica granite from the cupola of the Corupá Pluton, composed of Afs and Qz mainly, and containing Ca-amphibole (Amp), and **h** a syenite from the pink facies containing the same main mineral phases. **i** Representative large thin section of a syenite from the green facies, illustrating Afs crystal agglomerates and pairs supposedly formed via synneusis randomly arranged, and **j** closely packed Afs crystal aggregates constituted by individual specimens and pairs of crystals supposedly formed via synneusis. **k** Representative thin section of a syenite from the green facies with Afs crystals constituting closely packed glomerocrysts in which aggregates are supposedly formed via synneusis, and **l** closely packed Afs crystal aggregate exhibiting several pairs with a sheared face between them, supposedly formed via synneusis, and containing interstitial Amp, clinopyroxene (Cpx), and Qz. Dashed lines in **f**,**k**,**l** mark the plane of attachment of Afs crystals supposedly formed via synneusis (syn) and highlight crystals with Carlsbad twinning (twn).

The silicic volcanic rocks of the Campo Alegre-Corupá Basin and the composite syenitic-granitic Corupá Pluton represent cogenetic igneous rocks^27,28,31^. Whole-rock and zircon geochemical data support their interpretation as a single volcanic-plutonic complex (Campo Alegre-Corupá Volcanic and Igneous Plumbing System, CAC-VIPS), in which high-silica melts were extracted from a felspathic crystal mush, producing a silicic cumulate^9^. The silicic volcanic compositions include trachydacites, trachytes, and high-silica rhyolites (Fig. 1a–f), all related to the development and formation of a caldera-volcano^27,31^. These units comprise coherent to autoclastic lava flows and pumice-bearing pyroclastic sequences. Pre-caldera trachydacites and trachytes, as well as post-caldera crystal-rich trachytes, are typically porphyritic and contain euhedral alkali-feldspar (Afs) phenocrysts (Fig. 1d–f). Early erupted, crystal-poor, non-welded rhyolitic tuffs (~75–77 SiO_2_ wt.%) mark the onset of the caldera formation. The main caldera-forming pyroclastic sequences consist of crystal-poor to crystal-bearing (~5–15 vol.% Afs and quartz), poorly welded to rheomorphic lapilli tuffs (Fig. 1a–c), containing mafic and felsic cognate lithic fragments and crystals within a high-temperature devitrified ash matrix^27^. Monomineralic glomerocrysts of Afs and quartz occur throughout the volcanic successions, with Afs clots more common in trachytic lavas (Fig. 1e, f) and quartz clusters more abundant in the tuffs.

**Fig. 2:**
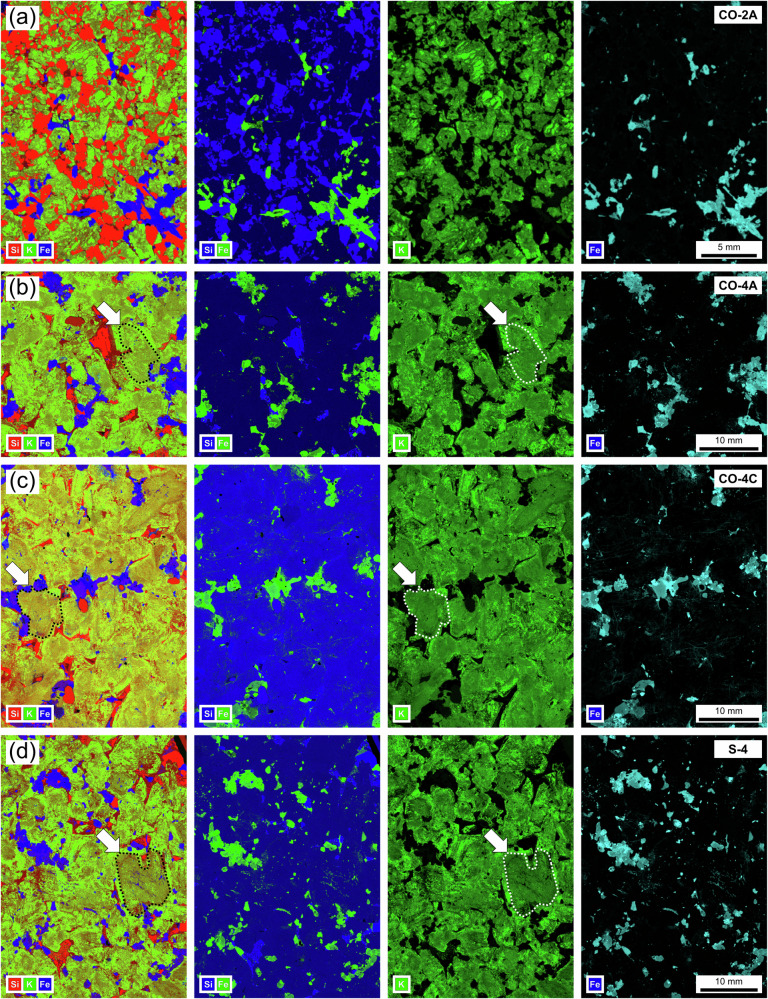
µ-XRF compositional maps from representative samples from the Corupá Pluton, including composite Si + K + Fe, Si + Fe, K, and Fe maps. **a** High-silica, medium-grained equigranular granite exhibiting euhedral to subhedral crystals of alkali-feldspar (Afs), quartz (Qz), and minor amphibole (Amp). **b–d** Syenites with relatively high (~67 SiO_2_ wt.%; pink facies), intermediate (~65 SiO_2_ wt.%; green facies), and low (~62 SiO_2_ wt.%; pink facies) silica contents are constituted of euhedral to subhedral crystals of closely packed Afs and interstitial Qz, Amp, clinopyroxene (Cpx), and minor Fe-Ti oxides and olivine. Note that in the syenites, the rims of Afs crystals are relatively enriched in potassium. **b–d** In the composite (Si, K, and Fe) and K maps, arrows pointing to dashed lines are used to illustrate Afs clots with common late-grown rims, which were excluded from the detailed quantitative textural analysis.

The Corupá Pluton consists predominantly of fine- to very coarse-grained syenites (~62-68 SiO_2_ wt.%), associated with a subordinate cupola of fine- to medium-grained, high-silica granite (~75–77 SiO_2_ wt.%), separated by a ~ 7 wt.% silica gap^9,32^ (Fig. 1g–i). The pluton displays vertical zoning from granite to syenite, marked by decreasing silica content and increasing grain size. The granitic cupola is composed mainly of alkali-feldspar (58.5 ± 6.8 vol.%) and quartz (37.3 ± 7.0 vol.%), with minor calcic-sodic to sodic amphiboles (3.5 ± 1.3 vol.%) and accessory ilmenite, chevkinite, apatite, and zircon (Fig. 2a). The syenites comprise two main facies (green and pink), the pink color reflecting hydrothermal alteration^32^. The subordinated green facies is composed of alkali-feldspar (85.6 ± 4.2 vol.%), quartz (4.4 ± 2.7 vol.%), clinopyroxene (hedenbergite; 4.5 ± 2.5 vol.%), calcic- to calcic-sodic amphibole (2.4 ± 1.0 vol.%), and olivine (fayalite; 0.3 ± 0.4 vol.%), probably representing an adcumulate. Conversely, the predominant pink facies consists of alkali-feldspar (82.0 ± 4.4 vol.%), quartz (5.0 ± 3.9 vol.%), hedenbergite and aegirine-augite (2.6 ± 1.4 vol.%), and calcic- to calcic-sodic amphibole (6.8 ± 3.2 vol.%), probably with ortho- to mesocumulate characteristics (Fig. 2b–d). Rare mela-syenites are composed of alkali-feldspar (34.7 vol.%), clinopyroxene (hedenbergite; 19.1 vol.%), olivine (fayalite; 28.5 vol.%), biotite (1.8 vol.%), apatite (5.6 vol.%), and magnetite (9.8 vol.%). All syenitic facies include closely packed Afs with potassium-rich rims (Fig. 2), along with late-crystallized K-rich Afs, quartz, granophyre, and accessory chevkinite, ilmenite, apatite, and zircon. Minor mafic occurrences are characterized by K-rich diorites that locally form hybrid intermediate rocks^32^.

### Quantitative textural analysis

**Fig. 3:**
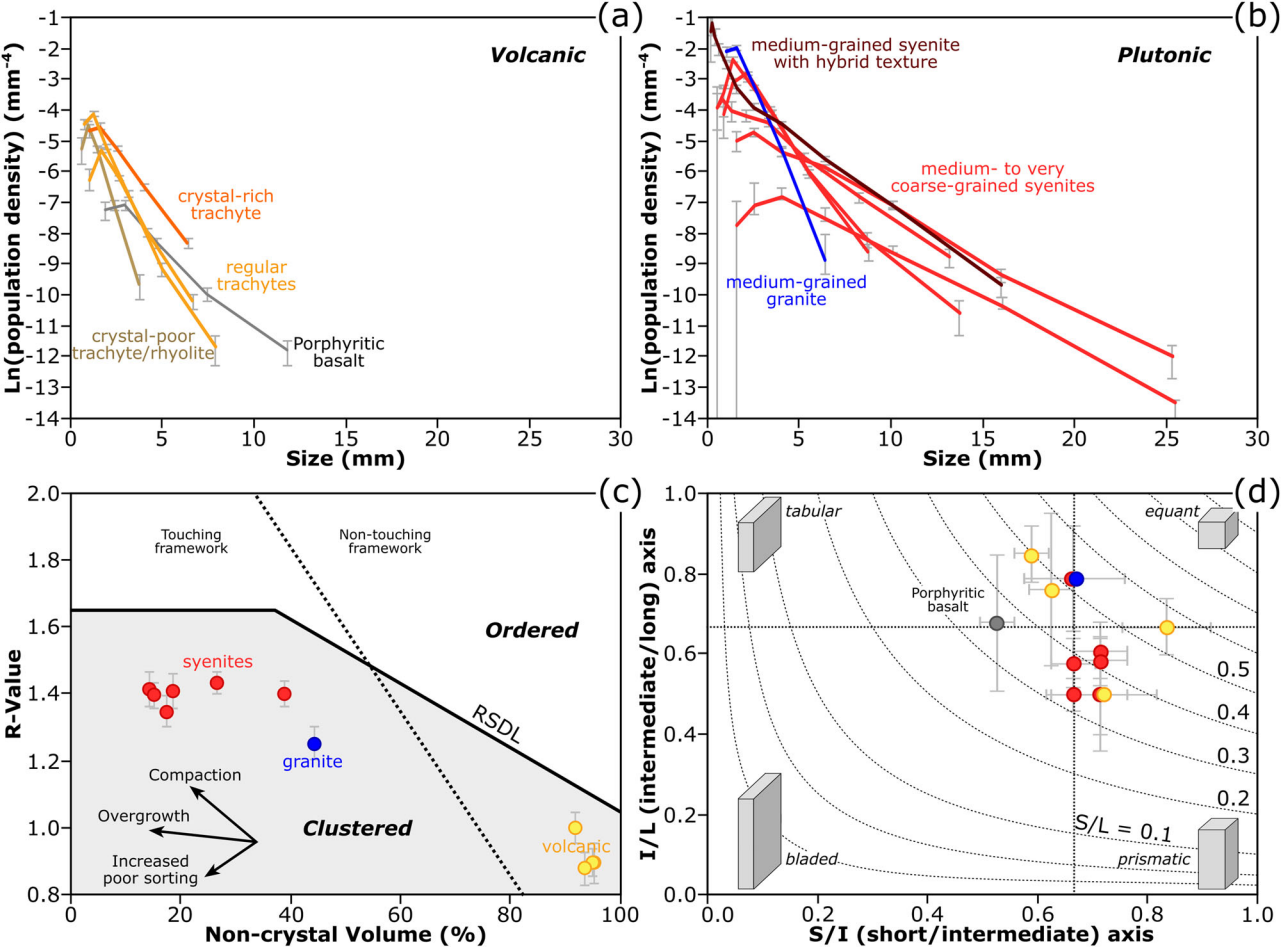
Quantitative textural measurements obtained from volcanic and plutonic rocks of the Campo Alegre-Corupá volcanic and igneous plumbing system (CAC-VIPS). **a**,**b** Crystal size distributions (CSDs) from volcanic and plutonic rocks of the CAC-VIPS. Note that CSD lines are constituted by multiple crystal size populations, each one with corresponding uncertainties (± 1σ standard deviation based on Poisson counting statistics) in their population densities. **c** Spatial distribution patterns (SDP) of alkali-feldspar constituting the volcanic and plutonic rocks from the CAC-VIPS. In this diagram, the non-crystal volume represents the complementary volume of other mineral phases in the bulk-rock, obtained through the following relationship: non-crystal = 100 - main phase vol.%. RSDL stands for Random Sphere Distribution Line, separating ordered from clustered distributions, and the arrows indicate the textural evolution caused by compaction, overgrowth, and increasing poor sorting (ref. ^36^), whereas the dashed line limits touching from non-touching frameworks (ref. ^30^). Note the gap between samples from contrasting realms and the overall textural evolution following trends of compaction and overgrowth form the silicic volcanics up to the syenites. Uncertainties are ± 1σ standard deviation. **d** Zingg Diagram as used to highlight the 3D shapes of crystals from their axial ratios, illustrating the habits of alkali-feldspar from silicic volcanic and plutonic samples, along with plagioclase from a porphyritic basalt. Note that alkali-feldspar crystals from the syenites are, in general, equant to prismatic, whereas in the volcanic samples and the granitic cupola, they exhibit equant to tabular forms. Corresponding uncertainties are given as 2σ SE (see Methods).

In the volcanic record, crystal size distributions (CSDs) of Afs phenocrysts were quantified in trachytic lava-flows erupted before and after the caldera-forming event(s), in a subvolcanic rhyolitic dike, and in a syn-eruptive densely welded lapilli tuff (see Conceptualization and Methodology, Supplementary Notes 1–6, Supplementary Figs. 2, 3 and Table 1). CSDs of plagioclase phenocrysts from a transitional basaltic lava-flow preceding the ignimbrite-forming eruption(s) were also measured for comparison (Fig. 3a). From the plutonic record, we analyzed one medium-grained granite and six medium- to coarse-grained syenites (Fig. 3b). Overall, CSDs display linear trends with typical convex-upward curvature at the smallest size fractions^33,34^. Regular trachytes (Fig. 3a red) and the medium-grained granite (Fig. 3b blue) show similar slopes but different Y-intercepts (*i.e*., final population densities). In contrast, crystal-poor and crystal-rich trachytes follow trends consistent with fractionation and crystal accumulation^34^, respectively (see Supplementary Notes 1). Plutonic samples define a fanning pattern from medium-grained granite to coarse-grained syenite, indicating progressive evolution marked by increasing residence times and/or crystal accumulation^34^. This pattern suggests a single crystallization stage accompanied by Ostwald ripening, reflected by more pronounced hump-shaped distributions in coarse-grained syenites^35^. A kinked CSD can only be observed in a medium-grained syenite sample with a hybrid texture. Afs crystals in the silicic volcanic units define, on average, five size populations, with crystal sizes ranging from 0.6 to 7.9 mm, and characteristic lengths (C_L_ = −1/slope of the CSD) between 0.53 and 0.87 mm. In the plutonic rocks, Afs crystals define seven size populations on average, with crystal sizes varying between 0.16 and 25.5 mm, and C_L_ in the interval between 0.69 and 3.25 mm.

Spatial Distribution Patterns (SDP) of Afs were estimated based on their relative modal abundances and clustering. Crystal clustering is expressed through R-values:

1 $$R=\frac{{r}_{A}}{{r}_{E}}=\frac{\sum r\left(1/N\right)}{1/(2\sqrt{{N}_{A}})}$$

where *r*_*A*_ and *r*_*E*_ are the observed and predicted nearest neighbor distances between crystals, which can be approximated by the second term of the equation, where *N* is the number of crystals, *r* is the distance to the nearest neighbor, and *N*_*A*_ is the number of crystals per unit area in a random distribution^36^ (see Conceptualization and Methodology and Supplementary Notes 2). Traditionally, R-values = 1, <1, and > 1 indicate random, clustered, and ordered crystal distributions, respectively. However, this depends on the Random Sphere Distribution Line (RSDL), which allows a better comparison with real distributions exhibiting clustered to ordered frameworks^30^. The different frameworks are delimited in R-value versus melt vol.% (equivalent to non-crystal vol.%) plots. Both volcanic and plutonic samples show clustered SDPs and follow the overall RSDL trend, although at lower R-values, likely reflecting relatively poorer sorting when compared to the trend of same-sized spheres (Fig. 3c). Cluster analysis further distinguishes touching and non-touching frameworks^30^: volcanic samples display non-touching frameworks, whereas plutonic samples show touching frameworks. CSD analysis assumes that 3D crystal populations can be inferred from 2D sections under specific conditions of random sectioning, simple crystal shapes, and textural homogeneity^34^. Despite these drawbacks, it also permits estimation of average 3D crystal habits defined by the long (L), intermediate (I), and short (S) axes^37^. Because 3D crystal habits are sensitive to magmatic crystallization conditions^34^, they can be used to explore crystallization kinetics through the ratios of their axes^38,39^. Estimated shapes indicate that Afs crystals are nearly equant (Fig. 3d), predominantly equant to tabular in volcanic rocks and the granitic cupola, and equant to prismatic in the syenites.

### Modeling textures based on crystallinity-ordering patterns

Our textural results, including similar CSD slopes with lower population densities in the volcanic record, fanning pattern in the plutonic realm, and complementary SDPs trends, indicate progressive cumulate formation through crystal clustering driven by (hindered)-settling, compaction, and/or repacking of Afs (Fig. 3; See Conceptualization and Methodology and Supplementary Notes 3). These observations reinforce previous interpretations that the CAC-VIPS represents a single, tiered volcano-plutonic system that underwent crystal accumulation and melt extraction^9,28^, in which the syenites of the Corupá Pluton formed as crystal cumulates, while rhyolitic and granitic rocks represent extracted residual melts. This framework allows us to address a key question: Do silicic plutons preserve a record of crystal accumulation and melt extraction, and how can it be recognized? Central to this approach is distinguishing early primocrysts from late overgrowth rims and interstitial crystals, which record different rheological regimes. Primocrysts crystallize in mobile, crystal-poor magma and record melt composition and dynamics prior to lock-up, while late interstitial crystals and rims form from trapped melt after lock-up, passively filling pore space and reflecting local melt composition. Failure to separate these populations can obscure textural signals of crystal accumulation, particularly in silicic systems with significant interstitial melt fractions. Accordingly, we manually separated primocrysts from overgrowths and small, anhedral late crystals using optical images and compositional maps (Fig. 2).

**Fig. 4:**
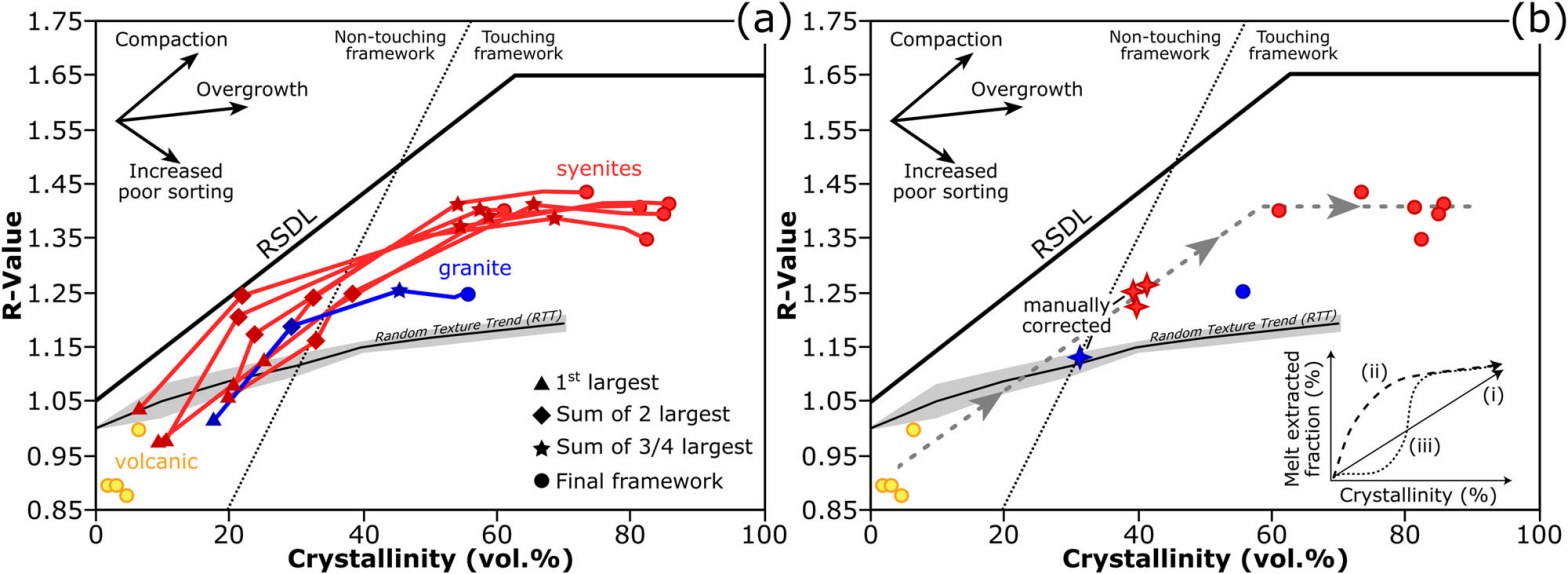
Patterns of R-Values versus crystallinities (vol.%) obtained for the volcanic and plutonic occurrences from the Campo Alegre-Corupá volcanic and igneous plumbing system (CAC-VIPS). **a**,**b** Composite spatial distribution patterns (SDP) lines and final bulk frameworks together with “manually corrected” textures. See text for a description of how the patterns were constructed. Trends of compaction, overgrowth, and increased poor sorting are after ref. ^36^ and touching/non-touching frameworks after ref. ^30^ RSDL stands for Random Sphere Distribution Line. Random Texture Trend (RTT) from ref. ^17^. illustrates the textural evolution of magmas crystallizing without mechanical modification, and the dashed arrows indicate the paths of ‘compaction’ and ‘crystal overgrowth’. The inset in (**b**) illustrates three schematic trends of cumulative melt extraction at contrasting crystallinities, with the melt extracted fraction being associated with crystal clustering through the R-values (see Fig. 4) and all trends plotted above the RTT. Case (i) represents a system in which the fraction of melt extracted increases linearly to higher crystallinities; in case (ii), the fraction of melt extracted is larger at relatively lower crystal contents, reaching an early plateau; and in case (iii), there is an increase in melt extraction at intermediate crystallinities, also reaching a plateau, but at a later point, commonly evoked by the classical mush model (modified after ref. ^7^).

Because larger crystal populations probably represent primocrysts, we reconstructed crystallinity-ordering patterns related to crystal-melt segregation by analyzing each size population separately (Fig. 4a). Modal abundances and clustering were determined from the straight segments of the CSDs (Fig. 3a, b). We adopted a stepped protocol, considering a progressive increase in Afs modal abundances starting with the larger crystal size populations, progressively adding smaller size populations to construct composite SDP lines that track textural evolution (see Supplementary Fig. 4). The largest Afs population ranges from 7 to 25 vol.%, and, based on their respective R-values, most fall within the Random Texture Trend (RTT^17^), except for two medium-grained syenites and the granite (Fig. 4a). When the second-largest crystal size population is included, most samples exhibit textures above the RTT, near the boundary between touching and non-touching crystal frameworks. After the integration of the three or four largest populations, the textures reach a plateau of around ~55 vol.%. They achieve R-values of *ca*. 1.40 in the syenites, after which crystallinity continues to increase until ~85 vol.%. Conversely, the granite reaches its maximum R-value of ~1.20 at *ca*. 45 vol.%, maintaining a nearly constant clustering index to ~55 vol.%. Maximum clustering values occur at 45–55 vol.% crystallinity, likely corresponding to the formation of a rigid framework at the Rigid Percolation Threshold^40,41^ (Fig. 4a). Importantly, crystallinity-ordering patterns in the volcanic record also suggest mechanical modification, probably owing to liquid gain or crystal loss (i.e. populations of crystals can be modified by compaction, sorting, mixing, etc. resulting in mechanically modified textures^34^; see Supplementary Notes 3). This is supported by the overall decrease in crystal clustering (R-values) when compared to the trend of static crystallization defined by the RTT.

Overall, the plutonic rocks describe multiple, yet similar, paths of textural evolution that correspond to a ‘compaction’ trend, marked by an increase in crystal clustering starting at low initial crystal contents (~10–20 vol.%) with a late, relatively minor influence of ‘crystal overgrowth’^36^ (i.e., flat segments in Fig. 4a), reaching high final crystallinities (~55–90 vol.%). This qualitative textural evolution is supported by the textural and compositional aspects of syenites, including closely packed Afs crystal clots, as well as K-rich crystal rims and small specimens with more evolved composition (Fig. 2). Manual separation of primocrysts yields “manually corrected” textures that likely represent the final rigid framework (see ref. ^22^. and Supplementary Notes 3-4), thus constraining the extent of mechanical modification. This correction accounts for post-cumulus crystallization and textural maturation reflected in the ‘crystal overgrowth’ segments of the composite SDP lines.

We applied the manual correction to four samples and, as expected, corrected textures show lower clustering and crystallinities (Fig. 4b). When combining both natural and manually corrected textures, we observe the same overall pattern of textural evolution registered in the composite SDP lines (Fig. 4a). Crucially, both patterns are consistent with relatively high amounts of extracted melts occurring during early stages of crystallization at low crystal contents [Fig. 4, (ii)-inset], differing from cumulates formed at a constant accumulation/melt-extraction rate [(i)-inset] and those formed in the extraction window at high crystallinities predicted by the mush model [~50-70 vol.%, (iii)-inset]. Moreover, the manually corrected textures exhibit modal abundances and clustering indexes that are similar, within uncertainties, to the average between the origin of the composite SDP lines (triangles in Fig. 4a) and the final population that define the ‘compaction’ trend (stars in Fig. 4a). Therefore, we applied this alternative simplified approach of averaging the trend of ‘compaction’ to our remaining samples from which we do not have compositional maps, to at least estimate their corrected textures to further check for mechanical modification^17^.

**Fig. 5:**
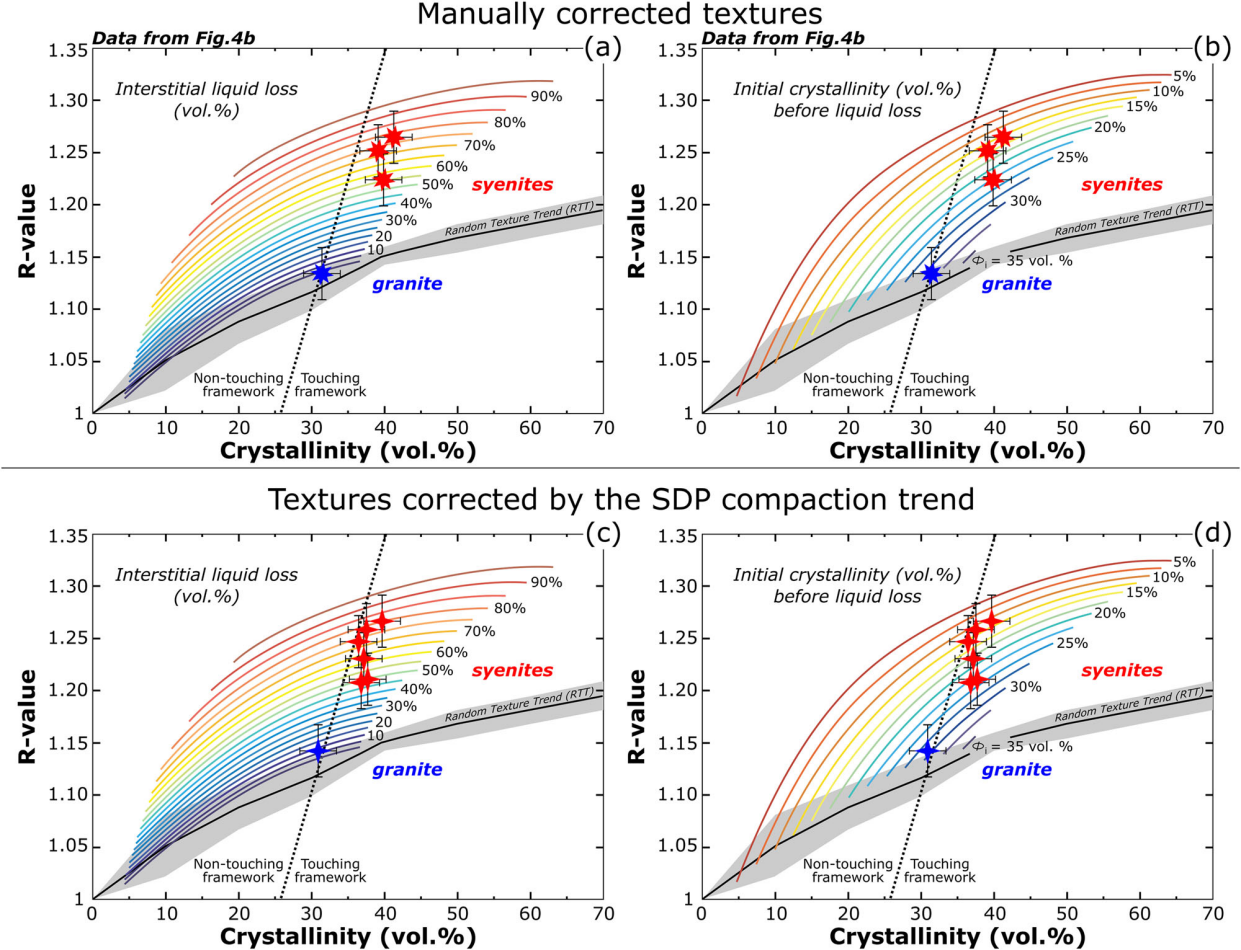
Estimation of interstitial liquid loss percentage and initial crystallinity before liquid loss. **a–c** Interstitial Liquid Loss (vol.%) and **b–d** Initial Crystallinity (vol.%) before liquid loss (diagrams from Špillar & Dolejš^17^). Samples in (**a–b**) are from Fig. 4b and were manually corrected for interstitial and rimmed alkali-feldspar, whereas textures in **c-d** were corrected according to the simplified method of mean R-values based on the composite SDP lines (see text for details). Note that, for both cases, the corrected textures align with the division between the non-touching and touching crystal frameworks of ref. ^36^. Error bars are 1σ SD computed for four equal-area subsections of the overall analyzed area.

Three-dimensional numerical simulations^17^ show that melt extraction from crystal mushes produces systematic increases in crystal spatial ordering that record compaction and rheological locking. By calibrating crystallinity-clustering relationships using their VoxelTex code, Špillar & Dolejš^17^ provide a quantitative framework to estimate the timing (*i.e*., initial crystallinity) and magnitude of melt extraction in natural magmatic systems. Applying this model to natural rocks requires prior textural filtering to isolate primocrysts, mostly because post-extraction overgrowths and late interstitial crystals overprint the original SDPs, leading to overestimation of melt loss (see Conceptualization and Methodology). Thus, based on the textural results obtained from both correction approaches described above, we can tentatively estimate the melt extraction percentage (MEP) and initial crystallinity before compaction in the analyzed samples using the diagrams of ref. ^17^. In the granitic cupola, Afs crystals reached the rheological lock-up at ~30.8 and 31.4 ( ± 2.5) vol.%, corresponding to R-values of 1.143 and 1.134 ( ± 0.025), considering the manual correction (Fig. 5a, b) and the simplified estimation (Fig. 5c, d), respectively. In the syenites, the same correction methods result in modal abundances varying from 39 to 41 ( ± 2.5) vol.% and R-values ranging from 1.225 to 1.265 ( ± 0.025) (manual correction, three samples), and crystallinities of 36 to 40 ( ± 2.5) vol.% with R-values ranging from 1.207 to 1.266 ( ± 0.025) (simplified correction, six samples), respectively. These results suggest a MEP varying from 0 to 8 ( ± 10) vol.% in the granitic cupola, and *ca*. 47 to 80 ( ± 10) vol.% in the syenites (Fig. 5). The initial Afs crystallinity before liquid loss varied from 29 to 32 ( ± 5) vol.% in the case of the granitic cupola, while in the syenites it ranged from 12 to 24 ( ± 5) vol.%. Note that all the corrected textures plot along the line separating non-touching from touching crystal frameworks (Fig. 5^30^).

Previous studies assuming static crystallization estimate that the primary rigid percolation threshold (rheological lock-up) varies between ~35–40 vol.%^16,17,30,36^, and 50–55 vol.% for more equant crystal shapes^40–42^. For crystallinities above this threshold, mechanical compaction is inhibited and demands mechanical failure, which was not observed (Fig. 1−2), or another mechanism to induce more effective mechanisms of crystal clustering and repacking^43^ (see next sections). Based on our textural measurements, all analyzed samples appear to have undergone mechanical compaction/crystal rearrangement until reaching the primary rigid percolation threshold (~35–40 vol.%). Furthermore, our results align with the predicted relationship between initial crystallinity and the attainment of the secondary rigid percolation threshold (see Supplementary Notes 4). Thus, possibly lower initial crystallinities allow a greater crystal-liquid segregation efficiency, mostly because crystals are free to move and rearrange their positions.

### Modeling compositional signatures associated with crystal-liquid segregation

**Fig. 6:**
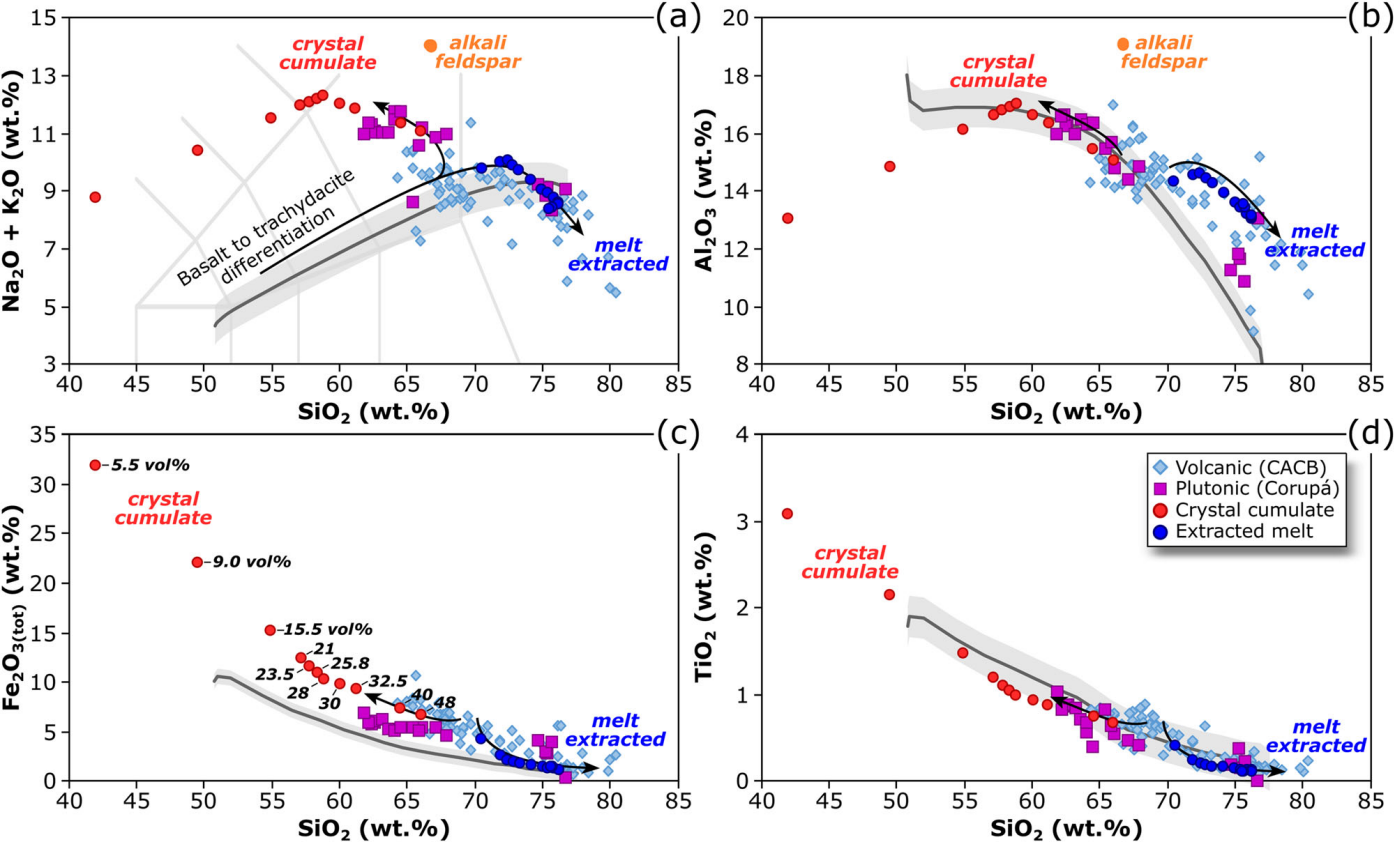
Natural composition patterns of major elements based on bulk rock compositions from the silicic plutonic and volcanic rocks of the Campo Alegre-Corupá volcanic and igneous plumbing system (CAC-VIPS). **a** Na_2_O + K_2_O wt.% as a function of SiO_2_ wt.%. **b** Al_2_O_3_ wt.% as a function of SiO_2_ wt.%. **c** Fe_2_O_3_ wt.% as a function of SiO_2_ wt.%, and volumes correspond to bulk solids in equilibrium with rhyolitic melts. **d** TiO_2_ wt.% as a function of SiO_2_ wt.%. Liquid line of descent (LLDs) from transitional basalt to trachydacite (continuous line) were modeled considering fractional crystallization at 100 MPa, with uncertainties as 1σ SD in the quantification of each oxide^9^. The modeled compositions for bulk solids (crystal cumulate) and residual liquids (melt extracted) were obtained by equilibrium crystallization at 200 MPa. In (**a**,**b**), compositions of alkali-feldspar obtained in the thermodynamic modeling demonstrate the influence of Afs in the overall LLDs, driving residual magmas towards alkali-poor compositions.

To evaluate the textural results, thermodynamic-based models^44^ were conducted to simulate the crystallization conditions leading to rhyolitic/granitic compositions, as well as their complementary silicic counterparts (see Conceptualization and Methodology). Following refs. ^9^^,^^28^, we adopt a trachydacitic parental magma (Supplementary Table 2) extracted from lower crustal levels as the best candidate to generate both the syenitic cumulate and the high-silica rhyolites/granite. This assumption is further supported by the presence of scarce fine-grained magmatic enclaves of similar composition (65–70 SiO_2_ wt.%) in the Corupá Pluton and the abundance of this composition in the volcanic record (Fig. 6a). Bulk-rock trends of the paired volcano-plutonic sequences, combined with modeling results, indicate that Afs exerts primary control on the LLD, with subordinate contributions from mafic phases. Modeled extracted melts evolve in a direction opposite to Afs composition (Fig. 6a, b), implying that Afs crystallization modifies bulk partitioning of K, Na, and Al and produces Y-shaped LLDs.

High-silica endmembers from both volcanic and plutonic domains of the CAC-VIPS are compositionally indistinguishable from magmas generated by basalt-to-rhyolite fractionation in the upper crust (Fig. 6). In contrast, complementary crystal-rich cumulates overlap compositions of basic-to-intermediate liquids along this modeled LLD. Deviations from a single LLD are restricted to oxides and elements that saturate during crystallization, particularly those forming Afs, as seen in the TAS diagram (Fig. 6a). Modeled bulk-solids (crystal cumulate) and residual liquids (melt extracted) with a silicic primary magma submitted to equilibrium crystallization at 200 MPa yield average compositions slightly less silicic than natural syenites (~55–60 SiO_2_ wt.%) and equivalent to the high-silica granites and rhyolites (~75–77 SiO_2_ wt.%), respectively. This model replicates the observed decline in Na_2_O + K_2_O and Al_2_O_3_ associated with Afs saturation registered by the silicic compositions. Thermodynamic modeling and mass-balance calculations indicate that syenites formed mainly through Afs accumulation, with minor olivine, clinopyroxene, and other accessory mafic phases. The syenites are interpreted as crystal cumulates consisting of variable proportions of modeled bulk solids (~50–75 wt.%) plus trapped residual melt (~25–50 wt.%), explaining their slightly higher silica contents relative to the model predictions (see Supplementary Fig. 5). These results corroborate the textural modeling and suggest that crystal accumulation and melt extraction probably started when the crystal fraction reached values between ~16–33 vol.% and proceeded until melt extraction attained a percentage of ~46–86 vol.% (±4; 1σ) of liquid, corresponding to a loss of ~47–96 wt.% of the residual melt.

### Where are the silicic crystal graveyards? Introducing the “*Dynamic Mush Model*”

Cumulates formed by crystal accumulation are commonly characterized by modal and textural layering^45,46^, and are typically associated with mafic to ultramafic systems. Silicic cumulates, in contrast, often exhibit subtler textures^18,19^ and are closely linked to melt extraction in the light of the mush model, particularly in the context of large caldera-forming eruptions^8,15,47^. This concept assumes that crystals remain suspended at low crystallinities but does not fully account for variations in crystal size distribution and buoyancy. Although some dynamic behavior is considered at <50 vol.% crystallinity due to convection^48^, the classical mush model predicts limited crystal-melt segregation efficiency, restricting melt extraction to a window of ~50–70 vol.%. Consequently, large crystal-poor silicic eruptions (100–1000 km³) would require proportionally larger silicic cumulate bodies in the upper crust to balance extracted melt volumes^1–4^. Solving this apparent imbalance is critical to understanding the generation of eruptible silicic magma beneath major caldera systems worldwide, including Taupō, Yellowstone, and Campi Flegrei. This motivates the search for alternative mechanisms capable of increasing the efficiency of crystal-melt segregation.

This study provides direct evidence for silicic cumulate formation during high-silica melt extraction (see next sections), leading to caldera development in the CAC-VIPS^9,27^. However, the relatively small volume of the Corupá Pluton (~50 km^2^ and ~600 m height, *ca*. 15–30 km^3^) compared with the volcanic sequences from the CAC-VIPS ( ~ 300 km^2^ and ~100–150 m thick, *ca*. 30–34 km^3^) implies that more efficient mechanisms of crystal-melt segregation might have operated at low crystallinities, consistent with our textural and compositional models. These results suggest that the melt-extraction window may be broadened and/or shifted toward lower crystallinities relative to classical predictions, reflecting dynamically evolving magma reservoirs. Textural features of the CAC-VIPS silicic cumulates, including randomly arranged, subhedral to euhedral Afs clots and synneusis pairs, absence of intracrystalline deformation, and downward increases in crystal size and sorting, indicate fluid-dynamic processes. Freely suspended faceted crystals must collide and adhere to form clots^29^, while crystal growth patterns indicate minimal solidification between injections, implying reservoir assembly under high growth rates. We therefore propose that the CAC-VIPS experienced recurrent influxes of under-accreted silicic magma pulses, promoting repacking-driven compaction through convective stirring^49^. The next sections propose a simple mathematical formulation for how these mechanisms lead to a more efficient melt extraction and discuss their implications.

### Crystal-melt segregation induced by upwards-moving magmas: the principle of elutriation

Cumulates require significant melt loss, but the mechanisms driving crystal clustering, such as settling and compaction, are fundamentally distinct. Here, crystal-clustering/compaction refers to repacking in a melt-dominated system, resulting from crystal settling and/or mechanical squeezing of interstitial melt (e.g., filter pressing)^30^. In contrast, viscous compaction^5,50^ results from the deformation of rigid crystal frameworks due to the own weight of crystal mushes, induced by magma flux^51^, or deformation-assisted segregation due to tectonics^23^, features not observed in our silicic cumulates. Thus, “compaction” here denotes crystal rearrangement within a mobile, melt-rich suspension.

Crystal-melt segregation is commonly attributed to settling and viscous compaction, which are often viewed from static and deformation-induced perspectives, respectively^1,5^. Although implicitly considered, these models often explicitly disregard, for simplicity, the roles of different magma emplacement and replenishment mechanisms and of reservoir growth dynamics, as active differentiation processes^52–54^. Here, we propose a conceptual dynamic model that combines melt-flush^55^, increased crystal clustering through either crystal attachment^29^, settling and/or mechanical compaction^30^, crystal coarsening^56^, and elutriation as key cumulate-forming mechanisms in rapidly growing magma reservoirs. Elutriation states that particles suspended in an upward-moving fluid either float or settle depending on their terminal velocities (*V*_*set*_ from Stokes’ law). Additionally, the destabilization of crystallization fronts can also result in sidewall crystal-laden density currents and/or crystal plumes within the magma reservoir^48,57–59^, and such a process can enhance the separation of particles from a moving suspension by elutriation. Crystal plumes within magma reservoirs are dynamic, convective features that play a critical role in crystal transport and magma evolution, particularly within mush-dominated systems^48^. These structures, often occurring as either downward-moving, crystal-laden magmas or as upward-moving, buoyant, melt-rich plumes, are key to understanding mush reorganization and the mechanisms by which melt can be extracted and ultimately erupt^57–59^.

We interpret the CAC-VIPS as a dynamic magma reservoir/chamber, subjected to nearly regular arrivals of new under-accreted, silicic magma pulses at relatively high growth rates, inducing repacking-driven compaction by convective stirring^49^. In the plutonic counterpart, crystal agglomerates are randomly arranged and mainly composed of large, subhedral to euhedral Afs clots and synneusis pairs. Intracrystalline deformation is absent, and internal textural variations show a downward increase in crystal size and sorting. Together, these features probably record multiple fluid-dynamic mechanisms. Thus, these textural records require magma motion, allowing freely suspended faceted crystals to collide and stick together^29^, while evidence of crystal growth suggests minimal solidification between successive injections, indicating a magma reservoir assembled at high growth rates and/or relatively slow cooling rates.

The same hydrodynamic mechanisms responsible for bringing and binding crystals together at lower-energy boundaries (i.e., dynamic/turbulent fluid-rich environment) can also disaggregate these clusters^60^. However, the perspective of a dynamic/turbulent, melt-dominated system in the development of crystal clusters and mushes has gained much attention and become a key point in the understanding of magmatic processes^51,61–64^. Supported by Stoke’s law, the terminal velocity of sedimentation at static conditions can be obtained by:

2 $${V}_{{set}}=\frac{g({\rho }_{{crystal}}-{\rho }_{{melt}}){d}^{2}}{18{{{\rm{\mu }}}}}$$

where *V*_*set*_ is the terminal settling velocity, *g* is the acceleration owing to gravity (9.81 m/s^2^), *ρ* is the density of bulk crystals (2700 kg/m^3^) and melt (2200 kg/m^3^), *d* is the particle diameter (assuming spherical shapes), and *µ* is the dynamic viscosity of the melt (for rhyolitic melts at ~3.0 H_2_O wt.%, *µ* can vary from 10^6^, 10^6.5^, up to 10^7^ Pa s). In non-diluted regimes (crystal fraction *c* > 0.1), the interaction between crystals is responsible for the decrease in settling velocities, as given by the equation for hindered settling: ${V}_{{hs}}={V}_{{set}}*f(c)$, where:

3 $$f\left(c\right)=\frac{{(1-c)}^{2}}{{({1+c}^{1/3})}^{[5c/3(1-c)]}}\,$$

see ref. ^65^. A limitation of hindered settling resides in the interaction between same-sized particles, which is inherited from Stokes’ law. Particles sinking can interact with each other, but if they all have the same diameter, they will all move downwards uniformly. However, if we consider a distribution of sizes following the log-normal behavior of crystals, as predicted by CSD theory, differences in the settling velocity of crystals of contrasting sizes will cause sinking crystals to mechanically interact with those that are floating, which can cause a decrease in their final settling velocities. This means that smaller crystals can be in suspension or even moving upwards, depending on the presence of an ascending magma flux.

Here, we derive the relationship between a critical particle size and the terminal velocity from Stokes’ Law to quantify the effects of upward-moving magma fluxes on crystal settling. For a given upward fluid velocity (*V*_*flu*_), there is a critical particle size at which *V*_*set*_ = *V*_*flu*_. Particles smaller than this threshold are entrained and carried upwards, while larger ones can settle (see Supplementary Notes 5). Thus, the net velocity of a crystal due to settling or buoyancy by elutriation can be expressed as: *V*_*elut*_ = *V*_*set*_ – *V*_*flu*_. Depending on the new crystal fraction, modified during magma replenishment, it can be obtained by:

4 $${V}_{{elut}}=\left[{V}_{{set}}*\left(1-\frac{{d}_{0}^{2}}{{d}^{2}}\right)\right]*f\left({c}^{{\prime} }\right)$$

where *V*_*elu*t_ is the elutriation velocity, *d*_*0*_ is the critical particle diameter, and *f(c’)* is the correction parameter computed for the new crystal fraction due to liquid gain or crystal loss.

**Fig. 7:**
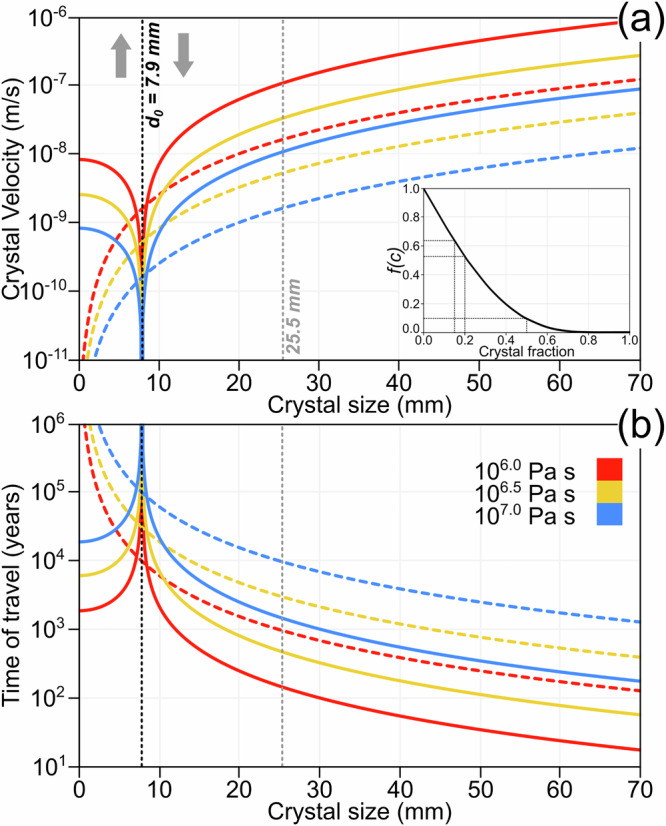
Absolute values of settling and elutriation velocities, and time of travel for varying crystal sizes. **a** Settling (*V*_*set*_, dashed lines) and elutriation (*V*_*elut*_, continuous lines) velocities (m/s) computed based on Stokes’ Law and considering an upward-moving magma flux in equilibrium with a critical particle size *d*_*0*_ = 7.9 mm. The velocities are a function of crystal size/diameter (m) and color-coded for calculations with varying magma viscosities (10^6.0; 6.5; 7.0^ Pa s). In the inset, the values of *f(c)* for crystallinities > 0.5 will reduce velocities to less than 10% of the terminal values computed from Stokes’ Law (*V*_*set*_) for highly diluted regimes (c « 1 vol.%). **b** Time scales (years) required for crystals with variable sizes/diameters to move over 500 m in a silicic suspension/mush, following ref. ^1^, considering the *V*_*set*_ (dashed lines) and *V*_*elut*_ (continuous lines). The vertical dashed lines in (**a**,**b**) represent the maximum crystal sizes recorded in the volcanic (7.9 mm, i.e., critical particle size for elutriation) and plutonic (25.5 mm) samples, respectively. Note that, for particles smaller than the critical value assumed in this study, crystal velocities are in fact ascending velocities, as indicated by the sense of motion recorded by the arrows, and the time of travel is the time to move 500 m, see text for discussion.

To test this formulation, we use maximum crystal sizes recorded in the volcanic sequences (7.9 mm) as an estimate of the critical particle diameter, assuming that magma fluxes were able to keep them suspended (Fig. 7). Terminal velocities of particles settling under ‘static’ conditions were also computed using the same variables, but assuming *c* = 0.5 to establish a comparison with the mechanism of hindered-settling^1^. The elutriation of smaller crystals depends on the velocity of the ascending magma flux, which results in the dilution of the crystal mush either through liquid gain or crystal loss, or a combination of both processes. Owing to the arrival of a new magma batch corresponding to ~40 vol.% of a restricted region of the bulk system for which *c* = 0.5, we estimate that the overall crystal fraction of both the upward-moving plume (*c’* ≤ 0.15) and the resident mush (*c’* ≤ 0.20) will result in relatively more diluted suspensions, compatible with our natural record. Thus, recharge can cause considerable changes in local crystal fractions, decreasing the *f(c)* and increasing the absolute velocities of particles.

Whether a particle will sink or float depends on the crystal size, with *V*_*elut*_ = 0 m/s at the critical particle size (Fig. 7a; Supplementary Notes 5). Importantly, smaller and larger crystals will result in rapidly ascending and settling velocities, respectively. Changing the velocity of the upwards-moving magma flux to values compatible with other critical particle sizes (see Supplementary Notes 5) will cause major variations for the elutriation velocities of the smaller crystals, with minor influence for large particles. Conversely, elutriation velocities for basalts are four orders of magnitude greater because viscosities are four orders of magnitude smaller when compared with rhyolites. In our example, for maximum crystal sizes recorded by the plutonic samples (~25.5 mm), *V*_*set*_ is in the range of 1.6 × 10−^9^ to 1.6 × 10−^8^ m/s, and *V*_*elut*_ is one order of magnitude greater, varying between 10^−8^ and 10^−7^ m/s. Following ref. ^1^, we also estimate the time required for a given crystal size to sink/ascend for over 500 m (Fig. 7b). The time of traveling varies from 1000 to 10,000 years using *V*_*set*_, and between 150 and 1500 years for *V*_*elut*_. Notably, the formation of glomerocrysts suggests that the effective particle sizes can be larger, which might exert a significant role in the terminal settling velocities. Assuming that crystals with the maximum sizes can constitute pairs formed via synneusis, resulting in effective crystal sizes of ~50 mm, *V*_*set*_ are in the range of 6.7 × 10−^9^ to 6.7 × 10−^8^ m/s, and *V*_*elut*_ are varying between 4.7 × 10−^8^ and 4.7 × 10−^7^ m/s. Conversely, their traveling times will vary from 240 to 2400 years using *V*_*set*_, and between 34 and 340 years for *V*_*elut*_. This suggests that considering the size distribution of particles is mandatory to establish terminal velocities and traveling times.

In summary, upwards-moving melt flow can act as a natural elutriation force, modifying crystal settling rates and counteracting Stokes’ law of sedimentation. It favors size/density-selective fractionation and may explain textural gaps in CSDs of erupted magmas. In silicic systems, elutriation might enhance crystal-melt segregation efficiency by selectively entraining smaller or less dense crystals in the ascending melt, while allowing larger crystals to settle more rapidly owing to a combined decrease in the local pressure (Bernoulli’s principle) and crystal fraction, critically influencing cumulate formation, melt differentiation, and the textural evolution of eruptible magmas. Additionally, the combined mechanisms of elutriation and crystal clustering/repacking can play a fundamental role in crystal-liquid segregation. However, the contribution of each one is difficult to estimate. In our case study, we hypothesize that crystal repacking might have played a more significant role in the bulk liquid extraction of syenites that register a more significant MEP, associated with particularly low initial crystallinities (~12 vol.%). Conversely, elutriation and hindered settling possibly have a more significant impact on the syenites registering lower MEP values at higher initial crystallinities.

### Assembly and growth mechanisms of large, silicic reservoirs that fed caldera-forming eruptions

**Fig. 8:**
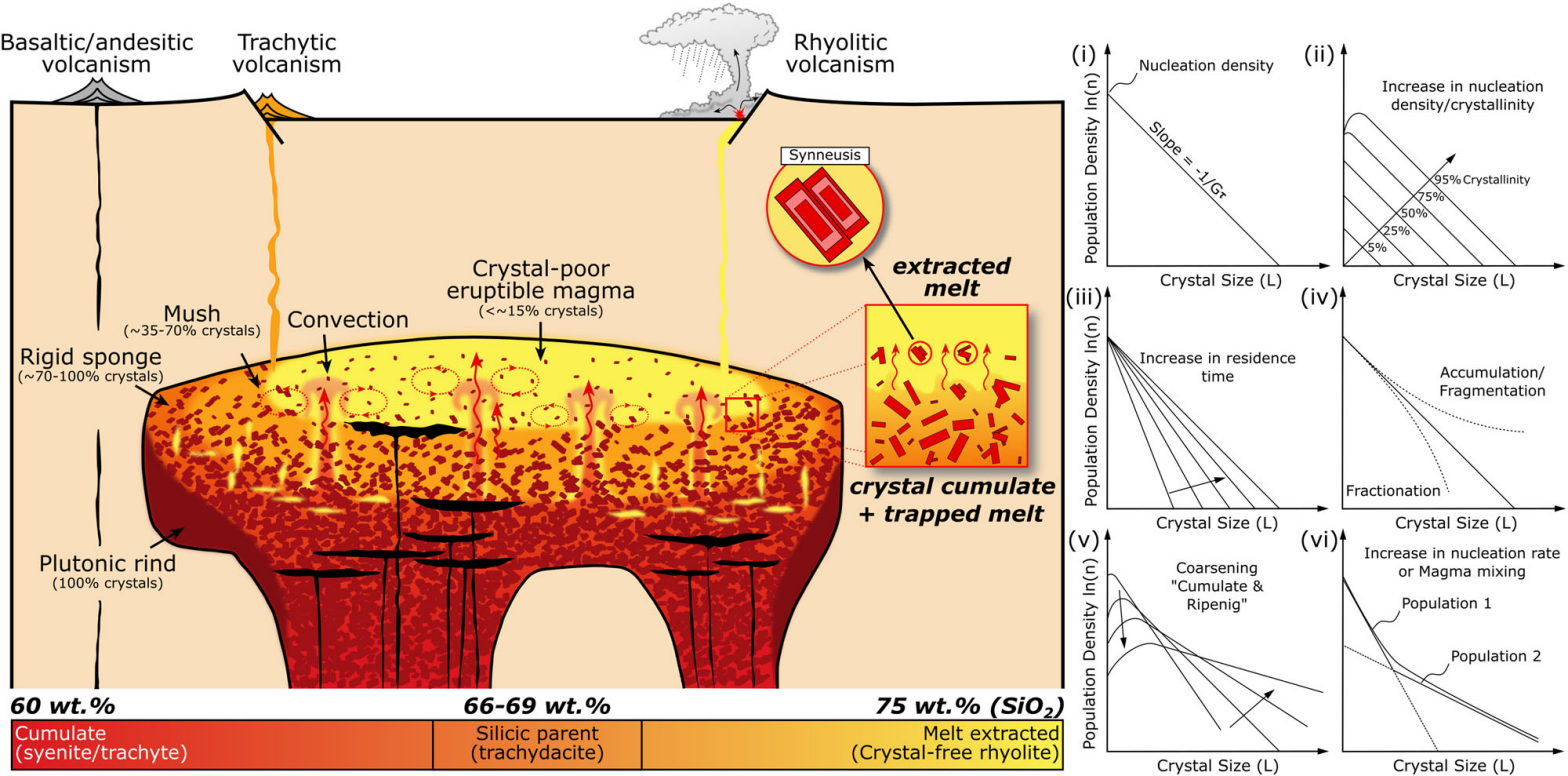
Schematic representation for the dynamic mush model. Representation of the compositional and textural evolution of a silicic reservoir built under dynamic conditions, and illustrations of the effect of different processes in the reservoir in schematic Crystal Size Distribution (CSD) patterns (i-vi). The inset demonstrates the formation of a crystal cumulate containing variable amounts of trapped melt associated with the formation of an upper cap constituted of the residual extracted melt. At the interface between the crystal-rich and crystal-poor domains, suspended crystals can attach to each other supposedly via synneusis (adapted from ref. ^47^, with permission from Elsevier). In such a magma reservoir/chamber, (i) crystals will nucleate and grow according to kinetics, following the log-linear distribution of sizes predicted by the CSD theory. Based on the internal distribution of crystallinities, (ii) the increase in the number of crystals will be accompanied by an overall increase in their relative population densities (n^0^), while (iii) the increase in their residence times will result in a fanning distribution of CSDs with a progressive decrease in the slopes of the CSD lines. If (iv) fractionation and crystal accumulation occur, the extracted melt will be able to carry most of the smallest crystals, whereas the cumulus will be constituted of a greater amount of the coarse-grained populations, leading to complementary bending of the CSD patterns. As soon as the rheological locking is achieved, and mechanically modified textures are no longer feasible, the textural maturation will be controlled by (v) equilibrium textures, such as coarsening. Finally, in a locally stirred/agitated environment, it is expected that different crystal populations will interact by mixing, resulting in (vi) mixed crystal-size populations.

The results above illustrate how the dynamic mush model has significant implications for the formation of silicic plutons that fed large caldera-forming eruptions (Fig. 8). It provides a mechanism for enhanced crystal-melt segregation, reducing the need for voluminous plutonic counterparts to balance massive eruptions, and explains how such reservoirs can evolve as a response to assembling mechanisms, such as under-accretion in the relatively cold upper-crust. Intermediate to high chamber growth rates (10^−3^ to 10^-^² km^3^/yr) and injection rates exceeding relaxation times^66^ likely promote heat accumulation, enabling rapid reservoir growth while suppressing nucleation of major mineral phases. Although mechanical compaction or crystal repacking accounts for lower crystal population densities in the syenites estimated by the CSDs, they do not explain the large size and equant shapes of Afs in structurally lower levels of the Corupá Pluton. These features likely reflect prolonged growth under low undercoolings^34,67^ and support reservoir growth through under-accretion of magma batches, maintaining higher temperatures in lower regions through advected heat, while Afs growth buffers temperature via latent heat release^68^.

Recurrent magma influx also governs chamber dynamics, best illustrated by comparing crystallization and settling times. Afs crystallization times can be approximated using CSD theory:

5 $${{{\rm{\tau }}}}=-1/({{{\rm{G}}}}\cdot {{{\rm{S}}}})$$

where G is the growth rate, and S is the slope of CSD lines^33,34^ (S = −1/C_L_ from Fig. 3; see Supplementary Notes 1). Using growth rates of 10^−10^, 10^−12^, and 10^−14^ m/s^69^, crystallization times for the volcanic samples range from ~0.17–0.40 years, 17–40 years, and up to 1700-4000 years; plutonic samples yield 0.2–1.0 year, 20–100 years, extending up to 2000-10,000 years. Because C_L_ reflects only the dominant sizes from linear CSD segments, these values exclude the time needed to reach maximum crystal lengths. Estimated growth to maximum sizes spans ~1.2–2.5 years, 120–250 years, and up to ~12,000-25,000 years in the volcanic record, and ~2–8 years, 200–800 years, and up to ~20,000-80,000 years in the plutonic samples.

Crystallization times therefore vary with emplacement mode, yet Afs growth durations for crystals with *d* > 7.9 mm (~2000–80,000 years) are of the same order of magnitude as settling times at their lower limit but exceed them by up to one order of magnitude at the upper limit (~100–10,000 years; Fig. 7). Undercooling drives crystallization but exerts contrasting control on nucleation and growth rates^34^, while residence time modulates kinetics under prolonged low cooling rates^67^. Thus, Afs crystals likely nucleated and grew under higher undercooling, probably in the upper magma chamber, as supported by their shapes and CSDs from the high-silica volcanic and plutonic occurrences. Alternatively, they may have crystallized in other low-crystallinity regions and been subjected to the constant arrival of new batches of magma, allowing them to attach via synneusis, for instance, and then settle into lower regions with prolonged low undercooling, resulting in large and more equant Afs crystals. In either scenario, the lower crystallization front may have acted as a compositional and mechanical filter, allowing small, buoyant crystals to float while promoting the growth of larger Afs crystals at lower growth rates. The resulting evolved melts can then migrate upwards and assemble the crystal-poor, eruptible “magma-pool” in the uppermost region of the magma reservoir (Fig. 8). In summary, based on crystal growth and segregation timescales, the CAC-VIPS magma reservoir likely assembled at intermediate to high chamber growth rates (10^−3^ to 10⁻² km^3^/yr^66^). As the CAC-VIPS was not emplaced into a thermally mature trans-crustal magmatic system^68^, the heat accumulation necessary for maintaining low undercooling must have been attained through advection by relatively high growth rates. In this way, it might have persisted as an eruptible magma pool for a short span, possibly between 10^4^−10^5^ years, and/or it was reactivated by a more mafic magma batch before eruption^9^.

Reservoir replenishment inevitably modifies compositional and textural signatures, especially in long-lived trans-crustal systems. Under such conditions, quantitative textural analysis should be integrated with in situ isotopic methods^25,52^ to resolve added complexities. Although our study supports the extraction of eruptible melt pockets from a shallow crystal mush^9,27,28^, the methodology can also quantify liquid gain or loss in holocrystalline rocks, refining estimates of volcanic-plutonic ratios in the geological record. Our findings imply rapid mobilization of large volumes of melt-dominated silicic magmas in the upper crust, possibly over ~10^2^−10^3^ to 10^3^−10^4^ years (Fig. 7). Crystal size, shape, and fraction strongly control permeability properties within mush zones. Thus, we show that elutriation and crystal attachment/repacking can be key mechanisms in the formation of crystal clusters during recharge-induced dilation and compaction of the crystal mush, driven by liquid gain and loss during magma replenishment. Based on these insights, we propose that large, silicic magma chambers are inherently dynamic systems in which differentiation occurs from their inception. It is likely a result of an interplay between emplacement mechanisms and fluid dynamics, driven by the feedback from crystallization kinetics, melt segregation, and reservoir growth. Finally, the studied volcano-plutonic system has been submitted to an efficient crystal-melt segregation process, ultimately resulting in a caldera infilled with crystal-poor, high-silica pyroclastic rocks^9,27^.

## Methods

### Conceptualization

**Fig. 9:**
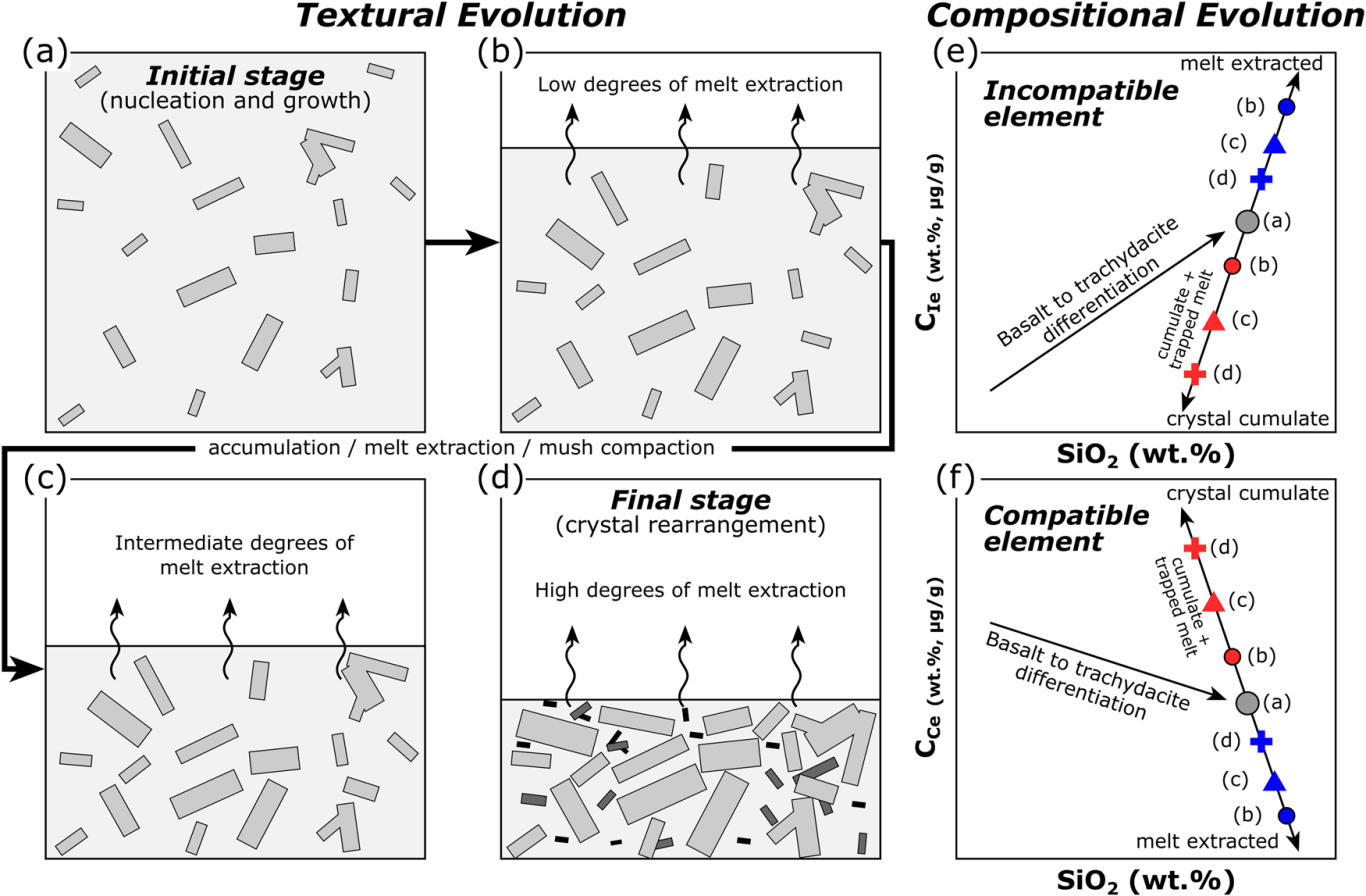
Schematic representation of paired textural and compositional evolution resulting from crystal-melt segregation. **a** Illustration of kinetically controlled (nucleation and growth) magma crystallization, leading to randomly arranged crystals of a single mineral phase. The population of crystals lacks a preferential orientation, and size distributions follow an overall negative logarithmic pattern as predicted by the crystal size distribution theory. **b–d** Low- to high-degrees of melt extraction can be associated with the mechanical expulsion or crystal rearrangement/repacking either at the initial or final stages of solidification. However, the residual liquids produced in the final stages of crystallization will represent more evolved compositions (enriched in incompatible elements). Note in **d** that crystal repacking can be accompanied by the nucleation and growth of new mineral phases if crystal-melt segregation occurs on similar timescales. **a–d** modified after ref. ^17^. **e-f** Illustration of compositional trends resulting from crystal accumulation and residual liquid extraction, as recorded by incompatible and compatible elements, respectively (modified after refs. ^13, 21^.). Note that, at low degrees of melt extraction and high crystallinities (case **b**), the extracted melts represent compositions that are relatively more evolved than the parental silicic magma (case **a**), whereas their complementary crystal cumulate retains a composition similar to the initial magma. On the other hand, cases of high degrees of melt extraction (case **d**) exhibit the opposite compositional pattern. Trends of differentiation in (**e**,**f**) simulate the evolution of transitional basalts towards the parental trachydacitic composition.

Identifying crystal-melt segregation in silicic rocks through igneous textures is challenging owing to limited quantitative methods, partly constrained by computer processing capacity^17^ and the scarcity of unambiguous, monomineralic silicic cumulate examples. Melt extraction from a mush yields two distinct products: a buoyant crystal-poor magma and a crystal-rich cumulate with variable trapped residual melt (Fig. 9a–d). This alters the spatial distribution patterns of crystals, increasing clustering and shifting textures from random to clustered crystal frameworks^17,36^, which can have varying degrees of clustering at different textural scales^30,70^. This process, driven by mechanisms, such as (hindered)-crystal settling, grain repacking (or mechanical compaction), viscous compaction, and crystal attachment^20,29,30,50,71,72^, is indicated by magmatic foliation, intracrystalline deformation, and the presence of crystal clusters, respectively.

In contrast, the crystal size distributions (CSDs) of paired extracted melts and cumulates will describe complementary paths^33,34^ (see Supplementary Notes 1–3). Normal CSDs of in situ crystallizing magma batches describe continuous log-linear patterns resulting from simple nucleation and growth mechanisms (kinetic textures), whereas the cumulates and extracted residual liquids show contrasting but complementary CSD patterns (mechanically modified textures). The extracted liquid will contain the smallest crystals kept in suspension, while the crystal cumulate will consist of the largest populations, resulting in contrasting size distributions in which CSDs exhibit different slopes^33,34^. Conversely, compaction (*sensu latu*) will increase the overall population densities of straight CSD lines, without affecting the size populations^73^.

3D numerical simulations by Špillar & Dolejš^17^ (see Supplementary Notes 4) demonstrate how melt extraction affects textural evolution in crystal suspensions with log-normal size distributions. Their models show that progressive melt loss systematically increases crystal spatial ordering (clustering/R-values), departing from textures formed by closed-system nucleation and growth, and thus allowing quantitative estimates of both the timing and magnitude of melt extraction. The simulations further show that melt segregation can occur over a wide range of crystallinities and may drive crystal mushes toward rheological locking at variable solid fractions, depending on their prior crystallization history. However, the approach relies on simplifying assumptions, notably the use of spherical, collision-equivalent crystals, which result in lower terminal porosities than natural systems and fail to account for post-cumulus crystal growth that gives rise to anhedral to subhedral crystal habits. As this model is designed specifically for systems with abundant phenocrysts, this raises the question: how can these theoretical models be applied to plutonic occurrences lacking porphyritic textures? Combining detailed textural and compositional analyses of cumulates to isolate primocrysts from overgrown rims and late interstitial crystals may provide the key (see Fig. 2).

### Textural analysis

Igneous textures from rocks constituting the Campo Alegre-Corupá Volcanic and Igneous Plumbing System (CAC-VIPS) were initially assessed through the qualitative, optical evaluation of rock slabs and regular (26 × 46 mm) to large (52 × 76 mm) thin-sections (Fig. 2). This was complemented by Scanning Electron Microscopy (SEM) and Micro-X-Ray Fluorescence (µ-XRF) compositional maps, performed at the *Laboratório de Caracterização Tecnológica*, USP, Brazil. SEM images were acquired using a QUANTA FEG 650 instrument, and compositional µ-XRF maps were produced with a Bruker M4 TORNADO Plus at 35 kV and 800 µA. Quantitative textural analyses were conducted for 12 samples, including five volcanic and seven plutonic occurrences. Within the plutonic record, we focused on syenites due to their wide compositional variability compared to the granitic cupola and because they possibly represent the silicic cumulates of the CAC-VIPS. In this sense, only one granitic sample was submitted to the detailed quantitative textural analysis, serving as a control sample. Rock slabs and thin sections were scanned at resolutions of 600 dpi and 2400 dpi, respectively. Crystal size distribution (CSD) analysis relies on stereological assumptions that allow 3D crystal populations to be inferred from 2D sections^34,73^, including random sectioning, simple crystal shapes, and textural homogeneity. Natural igneous systems commonly violate these assumptions through crystal clustering, fabric development, and post-crystallization reworking, requiring careful selection of primocrysts and cautious interpretation of CSD slopes.

Alkali-feldspar crystals were manually traced using InkScape® software (Figs. S2–3), with 300–1000 crystals outlined per sample to ensure statistical robustness of both shape and CSDs (> 250 crystals, see refs. ^37,38,74^). Crystals with less than ten pixels in area were discarded from the analysis, establishing the minimum measurable crystal size based on image resolution^34^, corresponding to approximately 0.05 ± 0.02 mm in equivalent crystal dimension. The smallest crystals retained in the dataset measured 0.34 ± 0.17 mm and 0.20 ± 0.10 mm along their major and minor axes, respectively. Furthermore, “manually corrected” textures were obtained through redrawing alkali-feldspar crystals by combining optical images and compositional maps with the aim of excluding overgrowths and late-grown crystals in the plutonic samples, which resulted in smaller populations (~200-400 crystals). The digitized images were analyzed using ImageJ^75^, and stereological corrections were performed with CSDslice^37^ to find the closest-matching shape curve to estimate 3D crystal habits. Further, the program CSDCorrections^76^ was used to compute Crystal Size Distributions (CSDs) and their respective uncertainties, and the Spatial Distribution Patterns (SDPs). Although our samples exhibit visible equant to tabular/prismatic 3D crystal shapes, as supported by petrography (Figs. 1–2), the accurate determination of three-dimensional habits is essential to stereological correction of 2D crystal size measurements into 3D crystal size distributions. As it was not possible to observe or measure any relevant crystal fabric using the Alignment Factor (AF, see Supplementary Table 1), stereological corrections were made considering no shape-preferred orientation.

For Afs crystals exhibiting nearly equant shapes, such as seen in the Cathedral Peak granodiorite, the 3D shape estimates made by the CSDslice^37^ spreadsheet are in good agreement with direct measurements of whole megacrysts (see ref. ^69^). Thus, using the five best-matched curves of 2D crystal size compared to the 2D measurements, uncertainties in crystal habits were estimated through the standard error (SE), computed from the standard deviation (SD) as: SE = SD/$\sqrt{n}$ (*n* = 5). The errors associated with the crystal shape ratios were propagated assuming SEs = σ_I_ and σ_L_, for intermediate (I) and large (L) axis. The SEs of the small (S) axis σ_S_ = 0 because there is no variation, since it is used as a normalization parameter. Uncertainties associated with I/L and S/I ratios were computed according to the following equations for error propagation:

6 $${{{{\rm{\sigma }}}}}_{\left(\frac{{{{\rm{I}}}}}{{{{\rm{L}}}}}\right)}=\sqrt{{\left(\frac{{{{\rm{\delta }}}}\left(\frac{{{{\rm{I}}}}}{{{{\rm{L}}}}}\right)}{{{{\rm{\delta }}}}{{{\rm{I}}}}}{{{{\rm{\sigma }}}}}_{{{{\rm{I}}}}}\right)}^{2}+{\left(\frac{{{{\rm{\delta }}}}\left(\frac{{{{\rm{I}}}}}{{{{\rm{L}}}}}\right)}{{{{\rm{\delta }}}}{{{\rm{L}}}}}{{{{\rm{\sigma }}}}}_{{{{\rm{L}}}}}\right)}^{2}}$$

*and*

7 $${{{{\rm{\sigma }}}}}_{({{{\rm{S}}}}/{{{\rm{I}}}})}=\sqrt{{\left(\frac{{{{\rm{\delta }}}}({{{\rm{S}}}}/{{{\rm{I}}}})}{{{{\rm{\delta }}}}{{{\rm{S}}}}}{{{{\rm{\sigma }}}}}_{{{{\rm{I}}}}}\right)}^{2}+{\left(\frac{{{{\rm{\delta }}}}({{{\rm{S}}}}/{{{\rm{I}}}})}{{{{\rm{\delta }}}}{{{\rm{I}}}}}{{{{\rm{\sigma }}}}}_{{{{\rm{L}}}}}\right)}^{2}}$$

assuming that the errors are not correlated. In this case, the partial derivatives are:

8 $$\frac{{{{\rm{\delta }}}}\left(\frac{{{{\rm{I}}}}}{{{{\rm{L}}}}}\right)}{{{{\rm{\delta }}}}{{{\rm{I}}}}}=\frac{1}{{{{\rm{L}}}}}$$

and

9 $$\frac{{{{\rm{\delta }}}}\left(\frac{{{{\rm{S}}}}}{{{{\rm{I}}}}}\right)}{{{{\rm{\delta }}}}{{{\rm{S}}}}}=\frac{1}{I}$$

10 $$\frac{{{{\rm{\delta }}}}\left(\frac{{{{\rm{I}}}}}{{{{\rm{L}}}}}\right)}{{{{\rm{\delta }}}}{{{\rm{L}}}}}=-\frac{{{{\rm{I}}}}}{{L}^{2}}$$

and

11 $$\,\frac{{{{\rm{\delta }}}}\left(\frac{{{{\rm{S}}}}}{{{{\rm{I}}}}}\right)}{{{{\rm{\delta }}}}{{{\rm{I}}}}}=-\frac{{{{\rm{S}}}}}{{I}^{2}}$$

Thus, the general formula for both estimations is as follows:

12 $${{{{\rm{\sigma }}}}}_{\left(\frac{I}{L}\right)}=\sqrt{{\left(\frac{{{{{\rm{\sigma }}}}}_{{{{\rm{I}}}}}}{{{{\rm{L}}}}}\right)}^{2}+{\left(-\frac{{{{\rm{I}}}}}{{L}^{2}}{{{{\rm{\sigma }}}}}_{{{{\rm{L}}}}}\right)}^{2}}$$

and

13 $${{{{\rm{\sigma }}}}}_{({{{\rm{S}}}}/{{{\rm{I}}}})}=\sqrt{{\left(\frac{{{{{\rm{\sigma }}}}}_{{{{\rm{S}}}}}}{{{{\rm{I}}}}}\right)}^{2}+{\left(-\frac{{{{\rm{S}}}}}{{I}^{2}}{{{{\rm{\sigma }}}}}_{{{{\rm{L}}}}}\right)}^{2}}$$

In the CSDCorrections^76^ program, the mutual Spatial Distribution Pattern (SDP) of crystals is based essentially on the nearest-neighbor statistics, which was implemented in a way that is consistent with how CSDs are measured on 2D sections and corrected to 3D population densities. CSDCorrections^76^ follows the ref. ^77^. nearest-neighbor formulation:

14 $$R=\,\frac{{r}_{A}}{{r}_{E}}$$

where r_A_ is the mean observed nearest-neighbor distance and r_E_ is the expected mean nearest-neighbor distance for a random (Poisson) distribution with the same number density. It results in a simple distribution of relative values for which R < 1 suggests a clustered distribution, meaning that crystals are closer than expected for a random spatial arrangement, R ≈ 1 suggests a random distribution, and R > 1 indicates that crystals are relatively more distant from one another than expected at a purely random framework. However, a more accurate interpretation depends on the distribution of varying crystal sizes, as this formulation was defined for same-sized particles. For computing r_A_, the software uses the centroids of all measured crystals (*i*) in the 2D section, and for each crystal it calculates the nearest neighboring centroid, which is further averaged:

15 $${r}_{A}=\,\frac{1}{N}{\sum }_{i=1}^{N}{r}_{i}$$

where N is the number of centroids considered in the measurement. Edge effects are minimized by using the counting frame defined by the user to exclude centroids of crystals that intersect forbidden boundaries. Conversely, for a random Poisson distribution of 2D particles, the expected mean nearest-neighbor distance can be obtained by the following:

16 $${r}_{E}=\,\frac{1}{2\sqrt{{N}_{A}}}$$

where *N*_*A*_ is the number of centroids used in the calculation per area.

Although the *r*_*E*_ values are computed considering CSDs to avoid the superposition of particles, the R-value is independent of crystal size as it only depends on centroid positions instead of lengths or areas. Because CSDCorrections^76^ links R-values to number density (n) and CSD slopes, the clustering index is often interpreted together with CSD curvature, not in isolation. Following ref. ^17^, values of R varying from 1 to ~1.2 at 0-70 vol.% crystallinities, respectively, suggest a steady-state random nucleation as seen in the Random Texture Trend (RTT) of Figs. 4–5. R-values below this threshold can be associated with crystal aggregation, synneusis, or heterogeneous nucleation, whereas greater values suggest mechanisms such as inhibited nucleation, textural equilibration through Ostwald ripening/coarsening, or compaction effects. There are some limitations to be considered, which include the fact that the R-values are 2D and not fully 3D, sensitivity of the results to image size, edge treatment, low counts of crystals (N <~50)^36^, and the necessity of correcting for crystal shape anisotropy. Finally, uncertainties were computed using the theoretical approach of ref. ^77^, who empirically derived the standard error of R-values for large N (N ≥ 100) as:

17 $${\sigma }_{R}=\,\frac{0.26136}{\sqrt{N}}$$

### Thermodynamic modeling

Tracking the compositional patterns described by the extracted melts and their complementary crystal cumulates depends on the compatibility of the elements considered and the extent of residual liquids trapped in the remaining crystal-rich counterpart^7,13^ (Fig. 9e, f). Thus, modeling the compositional evolution of silicic magma reservoirs requires some assumptions, such as starting composition, confining pressure, and oxygen fugacity (*f*O_2_). However, thermodynamic tools, such as the rhyolite-MELTS algorithm^44^ (v.1.1.x) can be useful to model the composition of paired residual liquids and solids crystallized/fractionated from any combination of initial conditions. In this study, we follow results from several recent investigations of the CAC-VIPS^9,27,28,31,68,78^, based on which we considered for modeling a parental silicic melt generated by the differentiation of transitional basaltic magmas at the Moho, which is further submitted to isobaric equilibrium crystallization in the upper-crust (200 MPa). The chosen trachydacitic starting composition likely represents the final product of the fractionation of a parental basaltic magma in depth (900 MPa) with relatively higher initial water contents (~0.9 H_2_O wt.%), which aligns with such compositions in the haplogranitic ternary^28^. The resulting silicic magmas are assumed to have 2.9 H_2_O wt.% and to have undergone isobaric crystallization under the Fayalite-Magnetite-Quartz *f*O_2_ buffer.

## Supplementary information

Supplementary Information

Transparent Peer Review file

## Data Availability

The authors confirm that the data supporting the findings of this study are available within the article and its supplementary materials.
