# Supplementary material for: Enhanced crystal-melt segregation within dynamic mush systems links silicic cumulates with caldera-forming eruptions
Source: Nat Commun. 2026 Jun 27;17:8060. doi: 10.1038/s41467-026-74812-4 (PMC13454170; doi:10.1038/s41467-026-74812-4)
Supplement: Supplementary file 1 [file 41467_2026_74812_MOESM1_ESM.pdf]

Supplementary Information for

Enhanced crystal-melt segregation within dynamic mush systems links silicic cumulates with caldera-forming eruptions

Lucas M. Lino[1*]; Francy R. Quiroz-Valle[1]; Silvio R.F. Vlach[1]; Olivier Bachmann[2]; Dougal A. Jerram[3,4]; Valdecir de A. Janasi[1]; Mathias Hueck[5]; Antomat Avelino Macêdo-Filho[1]; Miguel Â.S. Basei[1]

[1]Instituto de Geociências, Universidade de São Paulo – USP, Rua do Lago, 562, 05508-080, São Paulo, SP, Brasil
[2] Department of Earth and Planetary Sciences, ETH Zurich, Clausiusstrasse 25, CH-8092, Zürich, Switzerland
[3]NJORD Centre, Department of Geosciences, University of Oslo, PO Box 1028 Blindern, 0315 Oslo, Norway
[4]DougalEARTH Ltd., 31 Whitefields Crescent, Solihull B91 3NU, UK
[5]Institute of Geosciences, Ruhr-Universität, Bochum, Germany
*Corresponding author: lucas.martins.santos@usp.br

This PDF includes:

Supplementary Figures

Supplementary Figure 1 - Simplified regional geological map

Supplementary Figure 2 - Crystals outlined from high-resolution images obtained on rock slabs

Supplementary Figure 3 - Crystals outlined from high-resolution images obtained on a thin section

Supplementary Figure 4 - Representation of the method to obtain the composite SDP lines

Supplementary Figure 5 - Compositions of solids and melts from rhyolite-MELTs simulations

Supplementary Notes

Supplementary Note 1 - Crystal Size Distribution (CSD) theory and interpretation

Supplementary Note 2 - Spatial Distribution Patterns (SDP) theory and interpretation

Supplementary Note 3 - Assessing igneous textures in terms of crystal-melt segregation

Supplementary Note 4 - Melt extraction percentages based on crystallinity-ordering relationships

Supplementary Note 5 - Deduction of the elutriation velocity based on a critical particle size

Supplementary Note 6 - Supplementary 8-bit processed images used for CSD analysis

Supplementary Tables

Supplementary Table 1 - Crystal size distribution parameters

Supplementary Table 2 - Bulk-rock compositions of trachydacitic samples

Supplementary Figures to 'Enhanced crystal-melt segregation within dynamic mush systems links silicic cumulates with caldera-forming eruptions'

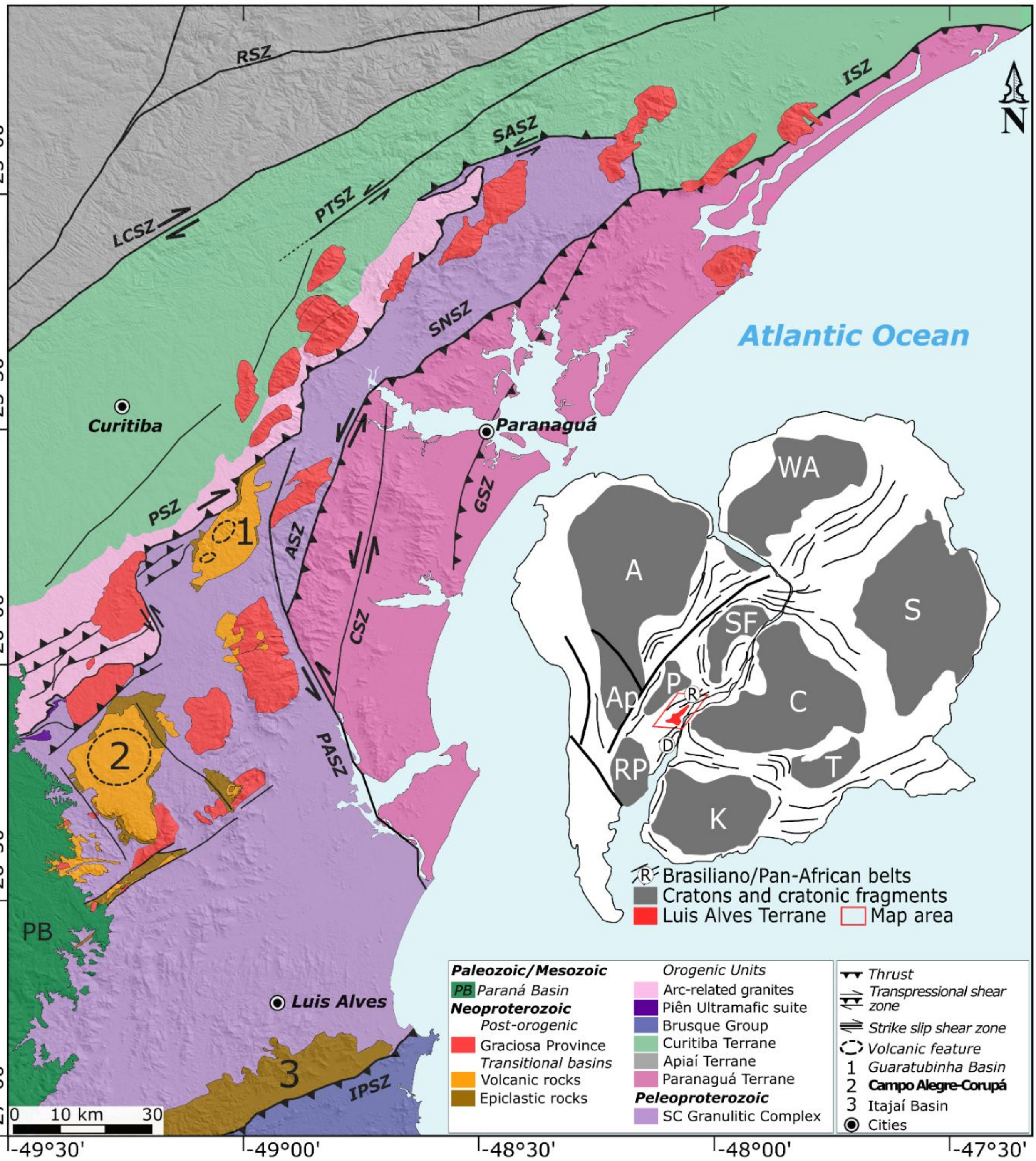

Supplementary Figure 1: Simplified regional geological/geotectonic map of southern Brazil, highlighting Neoproterozoic geological units and main structures. (Modified from Lino *et al.*[16], with permission from Elsevier). Shear-Zones (SZ): RSZ - Ribeira; LCSZ - Lancinha-Cubatão; PTSZ - Putunã; SASZ - Serra do Azeite; ISZ - Icapara; SNSZ - Serra Negra; PSZ - Piên; ASZ - Alexandra; GSZ - Guaratuba; CSZ - Cubatãozinho; PASZ - Palmital; IPSZ - Itajaí-Perimbó. Cratons: A - Amazonia; Ap - Rio Apa; C - Congo; K - Kalahari; Luis Alves (red); P - Paranapanema; RP - Rio de la Plata; S - Sahara; SF - São Francisco; T - Tanzania; WA - West-Africa. Orogenic Belts: R - Ribeira and D - Dom Feliciano. SCGC - Santa Catarina Granulitic Complex, the basement of the Luis Alves Terrane.

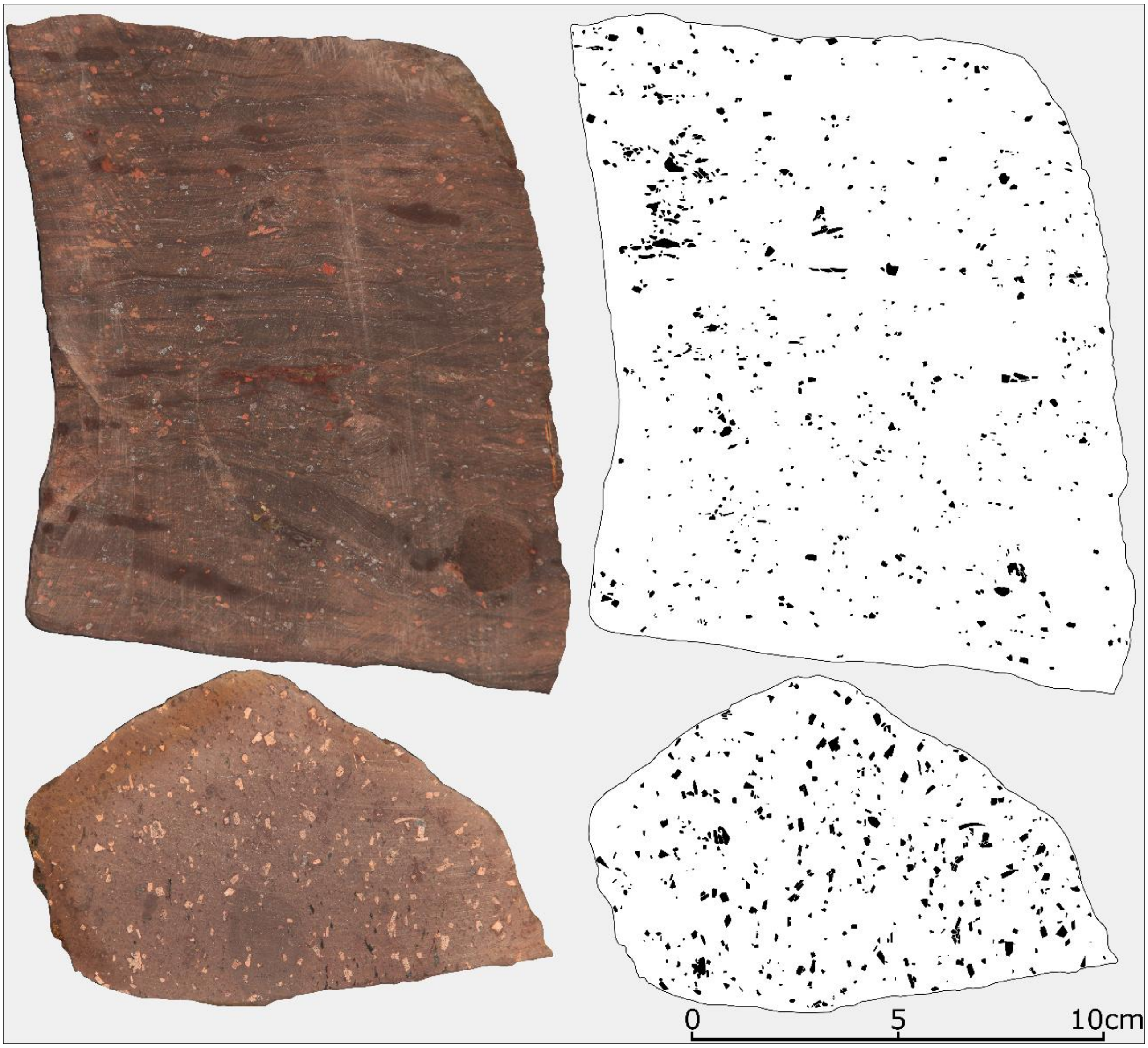

Supplementary Figure 2: Examples of alkali feldspar crystals outlined from high-resolution images obtained on rock slabs from a pumiceous pyroclastic flow and a trachytic, autoclastic lava flow, respectively. Both samples constitute the volcanogenic sequences associated with the caldera-forming eruptions registered in the Campo Alegre-Corupá volcano-plutonic system.

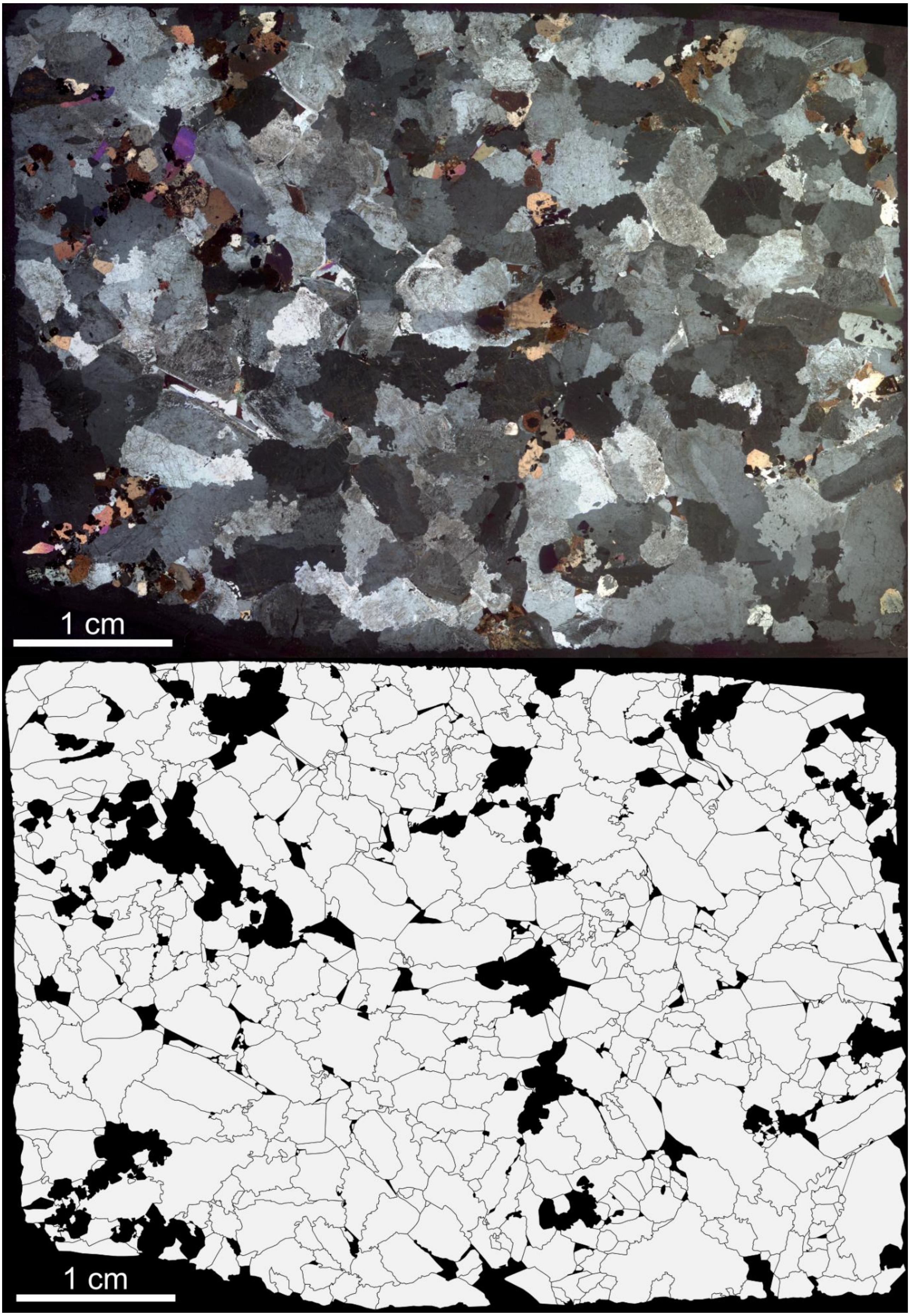

Supplementary Figure 3: Example of alkali feldspar crystals outlined from high-resolution images obtained on a thin section from a coarse-grained syenite from the Campo Alegre-Corupá volcano-plutonic system.

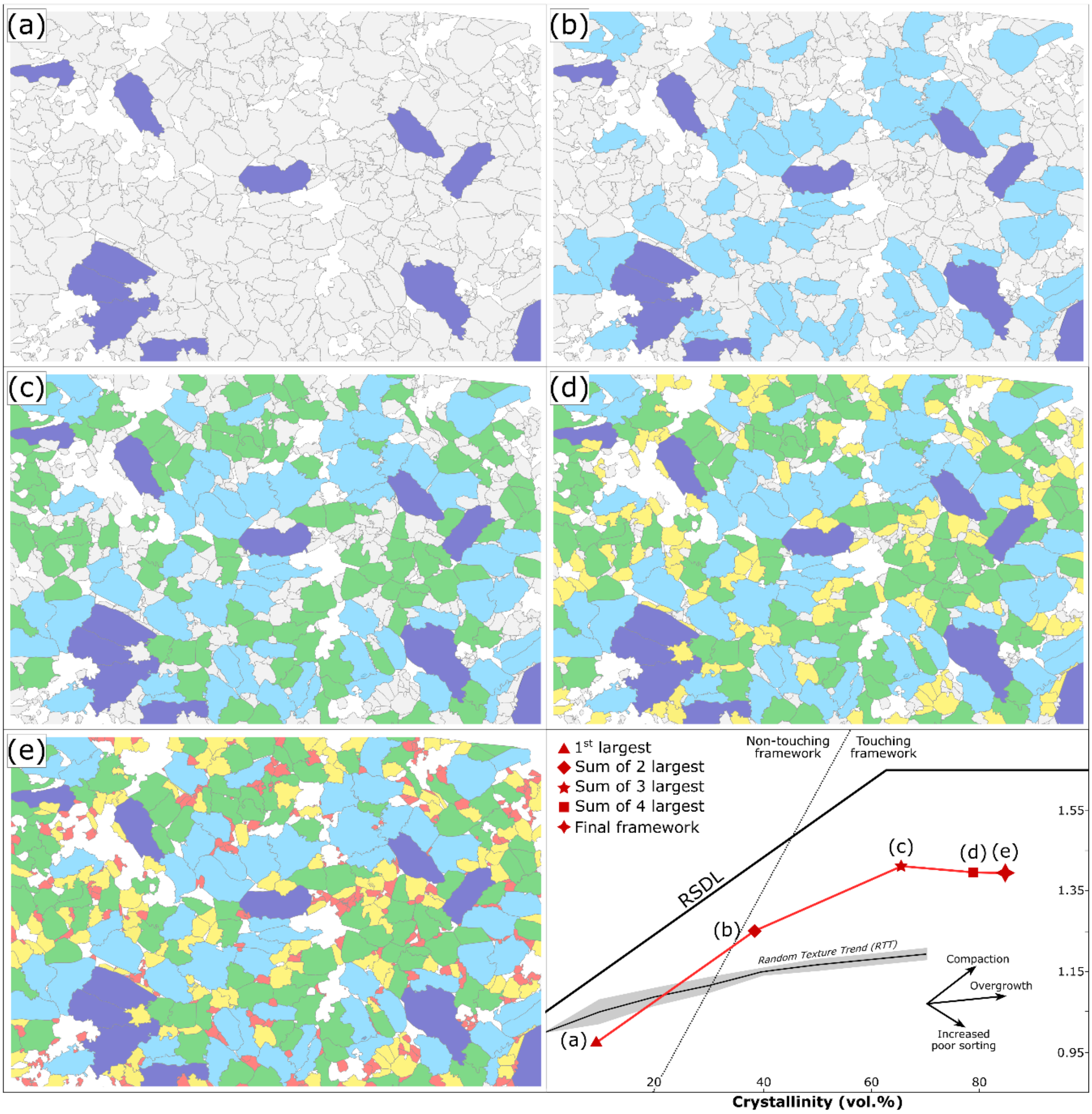

Supplementary Figure 4: Schematic representation of the method to obtain the composite SDP line of a medium-grained syenite submitted to a detailed quantitative textural analysis. Different colors represent each crystal size population, and they are included in the textural analysis progressively, starting from the largest one and including the following population. The represented composite SDP line was calculated for crystal size populations corresponding solely to the straight segments of its respective CSD.

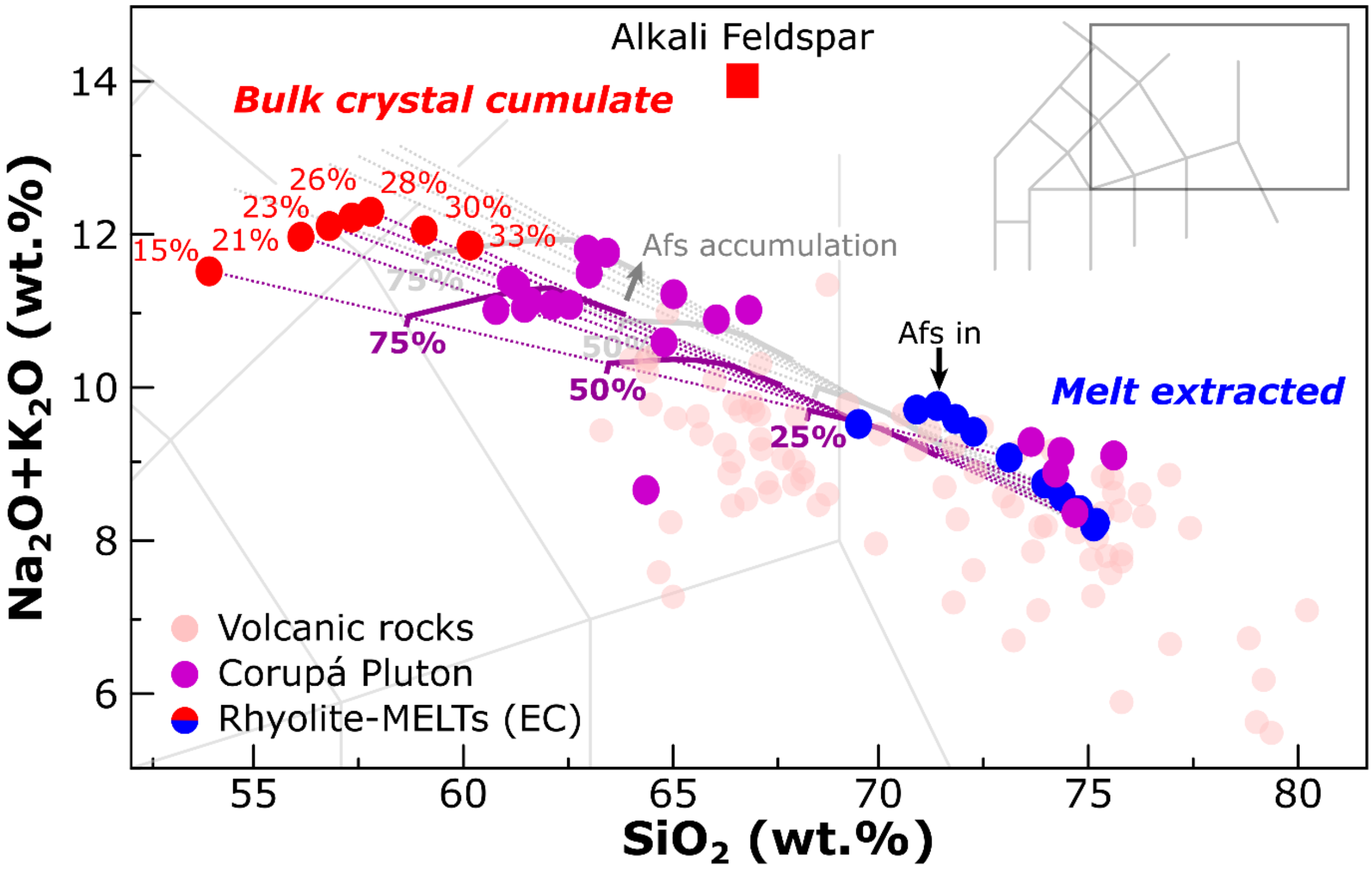

Supplementary Figure 5: Total alkalis versus silica diagram illustrating the resulting compositions of ***Bulk crystal cumulate*** and ***Melt extracted*** simulated with the rhyolite-MELTs algorithm for the equilibrium crystallization of a silicic, trachydacitic parental magma. Dotted tie-lines connect compositional pairs calculated assuming different fractions of melt extraction corresponding to varying initial crystallinities. Note that most of the bulk compositions of syenites from the Corupá Pluton can be generated by the mass balance between ~75% of bulk solids and ~25% of residual rhyolitic magmas, as represented by the purple dotted lines, for initial crystallinities varying from 15% up to 33%. Some exceptions can reach values close to ~50% of solids. For more alkaline syenitic bulk compositions, alkali feldspar (Afs) probably represents the main cumulative phase, as indicated by the light gray mass balance models and the dark gray arrow pointing to a preferential Afs accumulation. Conversely, trachytes can represent suspensions that also accumulated Afs crystals when compared with the residual rhyolitic magmas. Finally, for a crystal suspension containing 30% of crystals, the cumulate represented by 75% of solids and 25% of trapped residual liquid can be generated after the extraction of 60% of the residual melt, resulting in a Melt Extraction Percentage (MEP) of ca. 86%.

Supplementary Notes to 'Enhanced crystal-melt segregation within dynamic mush systems links silicic cumulates with caldera-forming eruptions'

1. Crystal Size Distribution (CSD) theory and interpretation

The Crystal Size Distribution (CSD) theory provides a quantitative framework to link crystal textures to the kinetics and dynamics of crystallization in magmatic systems. Supported by the previous work of Randolph and Larson[1] describing the industrial processes of crystal production, Marsh[2] and Cashman and Marsh[3] introduced and applied the CSD theory to interpret growth rates and crystallization times in steady-state magmatic systems. A CSD trend describes the population density of crystals as a function of crystal size, and it is commonly expressed as the number of crystals per unit volume per unit size interval, such that $n(L) = dN/dL$, where L represents a characteristic crystal dimension equivalent to their long axis or spherical diameter. In igneous systems, CSDs are typically plotted as $\ln[n(L)]$ versus L, which allows direct comparison with theoretical predictions derived from population balance equations. Thus, a linear correlation was recognized between the natural logarithm of the crystal population densities (n) and the size of the crystals (L). For a system undergoing continuous nucleation and growth under steady-state conditions, with constant effective crystal growth rate ($G^0 = dL/dt$) and constant nucleation rate (J), the solution to the population balance equation predicts an exponential size distribution, as expressed by the following:

$$n(L) = n_0 \, e^{\left(-\frac{L}{G^0 \tau}\right)} \qquad (Eq.1.1)$$

where τ is the effective residence time or crystallization interval of crystals in the magma, and $\ln(n^0) = J/G^0$ represents the Y-axis intercept and the final nucleation density extrapolated at $L = 0$. When plotted in logarithmic form, this relationship yields a straight line (Supplementary Figure 6a) described as:

$$\ln(n) = \ln(n_0) - \left(\frac{1}{G^0 \tau}\right) L \qquad (Eq.1.2)$$

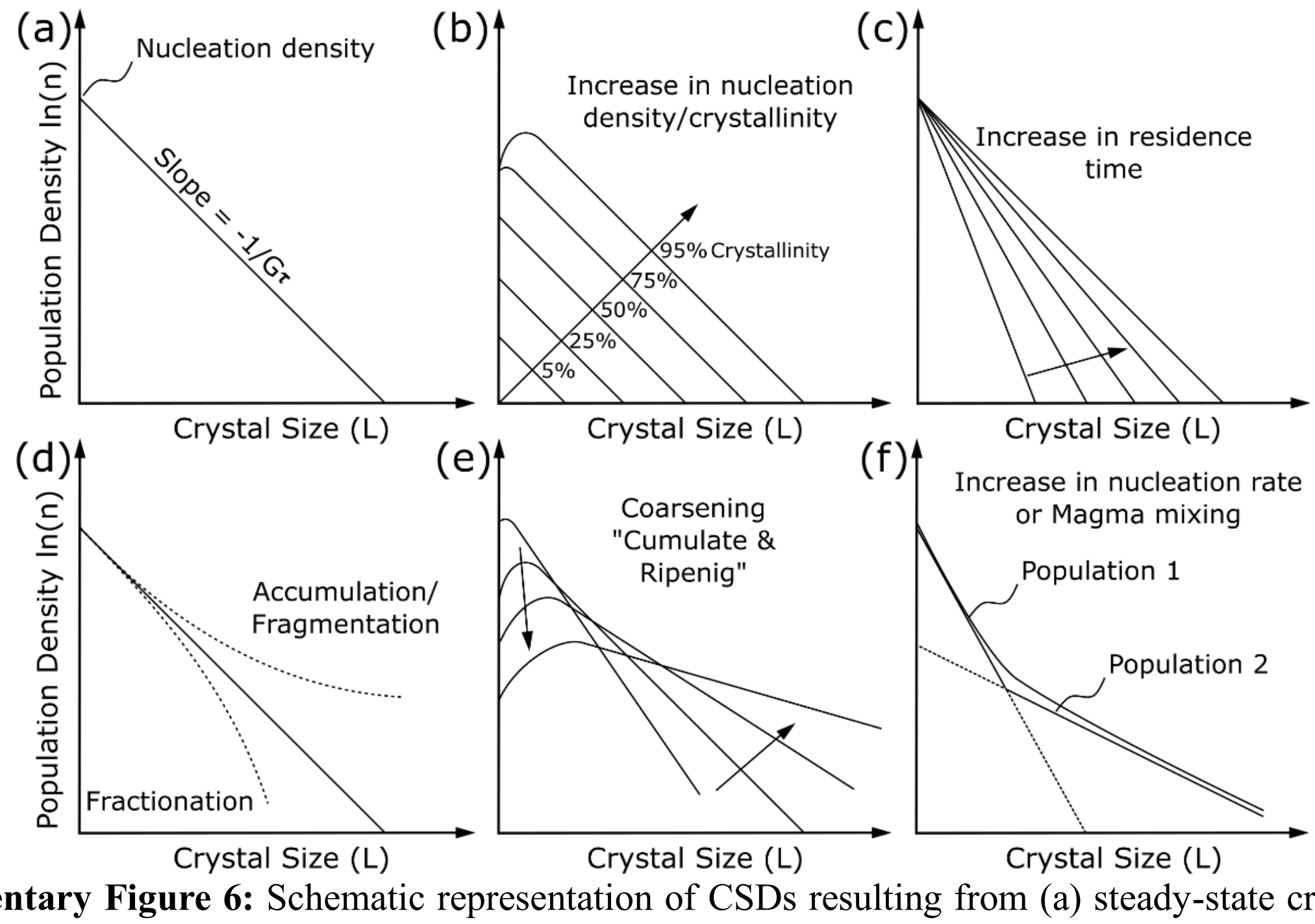

Supplementary Figure 6: Schematic representation of CSDs resulting from (a) steady-state crystallization, (b) increase in crystallinity as solidification proceeds in steady-state, (c) increase in residence time under constant nucleation rates, (d) accumulation of large crystals and fractionation smaller ones, (e) progressive equilibration textures resulting from cumulate formation and ripening, and (f) increase in nucleation rates of magma mixing resulting in two populations (see Higgins[4]).

Linear CSDs are therefore diagnostic of crystallization under approximately steady-state conditions and form the basis for quantitative interpretation of magmatic textures. Based on this relationship, the slope of a linear CSD ($-1/G^0\tau$) reflects the combined effects of crystal growth rate and residence time and corresponds to the characteristic length of crystals ($\bar{L}$). In short, steep slopes correspond to short residence times and/or slow growth rates, whereas shallow slopes indicate prolonged crystal residence or enhanced growth rates. As the growth rates are typically constrained within a limited range for a given mineral phase and magma composition, variations in CSD slope are commonly interpreted primarily in terms of differences in crystallization or storage timescales. In a log-linear plot of $\ln(n_0)$ versus L, the intercept [$\ln(n_0)$] represents the ratio between nucleation and growth rates and is therefore sensitive to the efficiency of crystal nucleation. Greater intercept values indicate high nucleation densities relative to growth, commonly associated with strong undercooling, decompression, or rapid changes in intensive variables. Lower intercepts reflect suppressed nucleation, annealing, or post-nucleation textural modification. Although ideal steady-state systems predict strictly log-linear size distributions (Supplementary Figure 6), many natural CSDs exhibit a systematic downturn or roll-off at small crystal sizes, characterized by lower population densities than predicted by steady-state nucleation and growth models[4-5]. This feature is generally not considered a primary crystallization signal, as continuous nucleation would otherwise produce high abundances of small crystals. Instead, depletion of small size classes is commonly attributed to post-nucleation processes, including Ostwald ripening, textural annealing during thermal relaxation, dissolution-reprecipitation, and crystal coalescence or clustering. Analytical and stereological limitations, such as sectioning effects and resolution thresholds, may further enhance apparent small-size depletion if not properly corrected[4-6].

Natural magmatic systems are certainly more complex than those predicted by the steady-state CSD theory because crystals often experience heterogeneous thermal and chemical environments, variable diffusion fields, and episodic recharge or reheating, all of which introduce departures from ideal steady-state behavior (Supplementary Figure 6d-f). As a result, log-normal CSDs are widely interpreted as the time-integrated outcome of stochastic crystal growth rather than as indicators of textural equilibrium. Departures from linearity, including curvature or hump-shaped CSD profiles, indicate non-steady-state crystallization. Such features reflect temporal variations in nucleation rate, growth rate, or residence time, and are commonly associated with magma recharge, reheating, crystal recycling, or prolonged storage in crystal-rich mushes[2,6,7]. Examples of non-linear CSD lines thus indicate extensive accumulation of large crystals (Supplementary Figure 6d), multiple cooling stages, and/or magma mixing[2,3,8,9]. Hump-shaped CSDs are widely interpreted as evidence for textural maturation processes, and they can include mechanisms such as ripening or partial dissolution, whereby intermediate crystal sizes are preferentially preserved while both small and large size classes are modified (Supplementary Figure 6e). In this sense, CSD analysis provides a direct link between crystal textures and the physical evolution of magmatic systems. For instance, CSD line inflections could indicate system reheating through the emplacement of a new magma batch, leading to more than one cooling stage, or magma mixing (Supplementary Figure 6f). As CSDs are controlled mainly by kinetic processes rather than by equilibrium thermodynamics, they can offer independent constraints on crystallization timescales, magma residence times, and post-crystallization modifications. When combined with careful stereological corrections and complementary geochemical data, CSDs represent a powerful tool for reconstructing the temporal and dynamic evolution of igneous rocks.

2. Spatial Distribution Patterns (SDP) theory and interpretation

The quantitative textural analysis of igneous rocks can also include the characterization of spatial distribution patterns and crystal packing, in addition to crystal sizes, shapes, sorting, and fabrics. Thus, it is able to disclose significant information concerning the mechanical processes that magma undergoes during solidification[4,10]. The spatial distribution pattern (SDP) of crystals describes how crystal centres are arranged relative to one another within a rock, and provides information that is complementary to crystal size distribution (CSD) analysis. Whereas CSDs quantify crystal population densities as a function of size, SDP analysis characterises the degree of ordering, randomness, or clustering in the spatial arrangement of crystals and, therefore, it is sensitive to processes such as crystal accumulation, clustering, compaction, and post-crystallization modification[9,10]. SDP analysis is based on spatial statistics originally developed to characterise point patterns in ecological and physical systems[11] and later adapted for petrographic applications[4,10]. In rock textures, individual crystals are represented by their geometric centres in a two-dimensional thin section, and the spatial relationships between these centres are used to describe the packing and arrangement of the crystal population (Supplementary Figure 7). The fundamental assumption of the method is that different geological processes produce distinct spatial arrangements of crystals. Ordered packings arise from efficient mechanical re-arrangement or sedimentation of individual grains, whereas clustered or chained distributions reflect aggregation of crystals, synneusis, heterogeneous nucleation, or accumulation of crystal clusters rather than single grains[10].

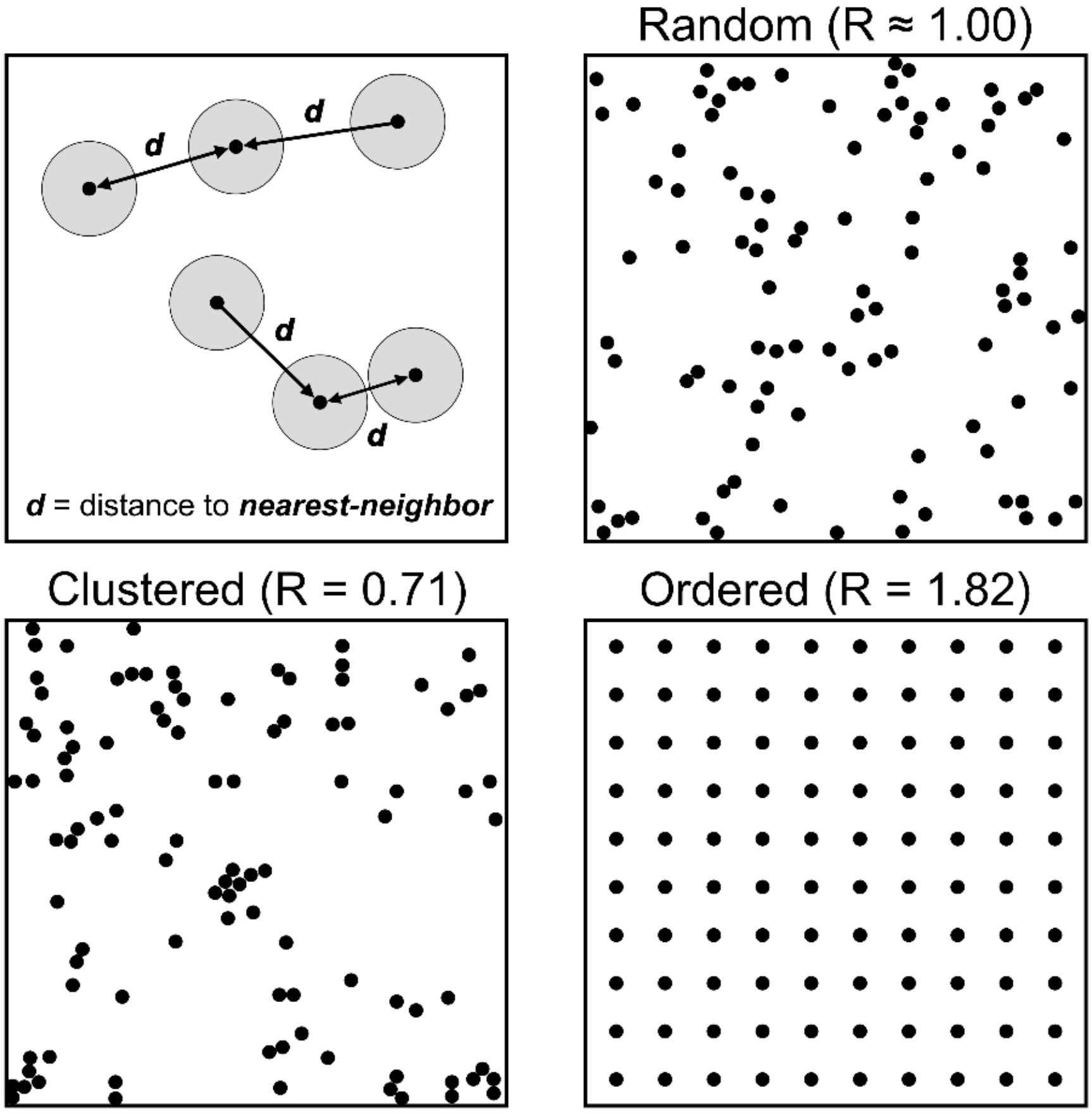

Supplementary Figure 7: Schematic representation of the nearest-neighbor distance (*d*) between crystal centers as defined in digitalized thin sections. Based on the average distance between nearest-neighbor crystals (r_A), three examples of distributions of points in 2D sections can be found based on the R-values. A random distribution is represented by R-values close to the unit, whereas a clustered and ordered arrangement of points results in R-values smaller and greater than 1, respectively. Modified after Jerram *et al.*[10].

The SDP of a crystal population is quantified using the nearest-neighbor method of Clark and Evans[11], adapted for petrographic textures by Jerram *et al.*[10]. For each crystal in a thin section, the distance between its center and the center of its closest neighboring crystal is measured (Supplementary Figure 7). The mean nearest-neighbor distance for the population (r_A) is then calculated as:

$$r_A = \frac{1}{N}\sum_{i=1}^{N} r_i \qquad \textit{(Eq.2.1)}$$

where r_i is the nearest-neighbor distance for crystal *i,* and N is the number of crystals measured. This observed mean distance is compared to the expected mean nearest-neighbor distance (r_E) for a random Poisson distribution of points with the same number density:

$$r_E = \frac{1}{2\sqrt{N_A}} \qquad \textit{(Eq.2.2)}$$

where N_A is the areal number density of crystals. The ratio between the observed and expected mean distances defines the dimensionless clustering-index parameter R:

$$R = \frac{r_A}{r_E} \qquad \textit{(Eq.2.3)}$$

The R-value provides a quantitative measure of spatial ordering: values of $R > 1$ indicate ordering, values close to the random reference indicate random packing, and values $R < 1$ indicate clustering or chaining of crystals.

In contrast to idealized point distributions, crystal centers in thin sections cannot occupy arbitrarily close positions because crystals exhibit finite sizes. As a result, a random distribution of grains does not correspond to $R = 1$, and instead, R-values yield systematically greater than unity. To account for this effect, Jerram *et al.*[10] defined a reference framework based on two-dimensional sections through three-dimensional randomly packed distributions of equal-sized spheres. The R-values obtained from such models vary systematically, with the non-crystal fraction (= 100 vol.% - crystal fraction, referred to as porosity by Jerram *et al.*[10] defining a reference curve known as the *Random Sphere Distribution Line* (RSDL). This line represents the expected SDP for random packing of solid grains at different crystal fractions and provides a baseline against which natural rock textures can be compared. Textures that plot above the RSDL are interpreted as having an ordered spatial distribution, whereas textures plotting below the RSDL are interpreted as clustered. This approach allows the SDP to be evaluated independently of crystal abundance and avoids misinterpretation of R-values based solely on absolute magnitude[10]. It is worth mentioning that these distributions are relative to a structure composed of solid particles of the same size, and the division between clustered and ordered frameworks at $R = 1$ corresponds to a crystal fraction that tends to zero. More accurate analysis of these distributions can be made by comparing crystal frameworks with those resulting from a steady-state random nucleation and constant growth of crystals, as seen in the *Random Texture Trend* of Špillar and Dolejš[12].

Ordered SDPs are characteristic of systems in which crystals are deposited or rearranged as individual grains, such as gravitational settling, efficient mechanical re-ordering, or high-energy accumulation processes. In contrast, clustered SDPs indicate aggregation of crystals into chains or clusters before or during accumulation, synneusis, heterogeneous nucleation, or limited mechanical reworking of the crystal framework[10]. Importantly, SDP analysis is sensitive to processes occurring after the crystal mush achieves the rheological lock-up. Mechanical compaction tends to increase R-values by promoting more efficient packing, whereas deformation-induced fabrics may reduce R-values in sections parallel to the principal stress direction.

Crystal overgrowth can also modify R-values by shifting the geometric centers of crystals as grain morphology evolves, typically leading to increasing R-values with increasing crystallinity[9,10]. SDP analysis is most powerful when combined with CSDs and modal data. Whereas CSD slopes constrain crystallization timescales and kinetic histories, SDP analysis provides independent constraints on the spatial organization of crystals and the physical mechanisms responsible for crystal accumulation and modification. The combined use of CSD and SDP, therefore, permits discrimination between processes such as crystal settling, clustering, compaction, and in situ overgrowth, all of which may produce similar modal or size distributions but distinct spatial arrangements.

3. Assessing igneous textures in terms of crystal-melt segregation

In the study of igneous textures, three main categories emerge from the most frequent mechanisms that modulate them (Supplementary Figure 8). These are (1) kinetic textures, which result from nucleation and growth processes and are controlled by the undercooling and saturation of the crystallizing phases; (2) mechanically-modified textures, resulting mostly from sorting and compaction of the existing crystalline phases; and (3) equilibrium textures, which are mostly modulated by coarsening, overprinting the preexisting textures[4]. In this sense, both kinetic and mechanically-modified textures may later undergo some degree of equilibration for example, through coarsening, and the resulting textures can be indistinguishable from each other. Thus, recovering the crystallization path of igneous rocks is crucial to identifying the mechanisms responsible for the textural and compositional evolution of magmatic suites.

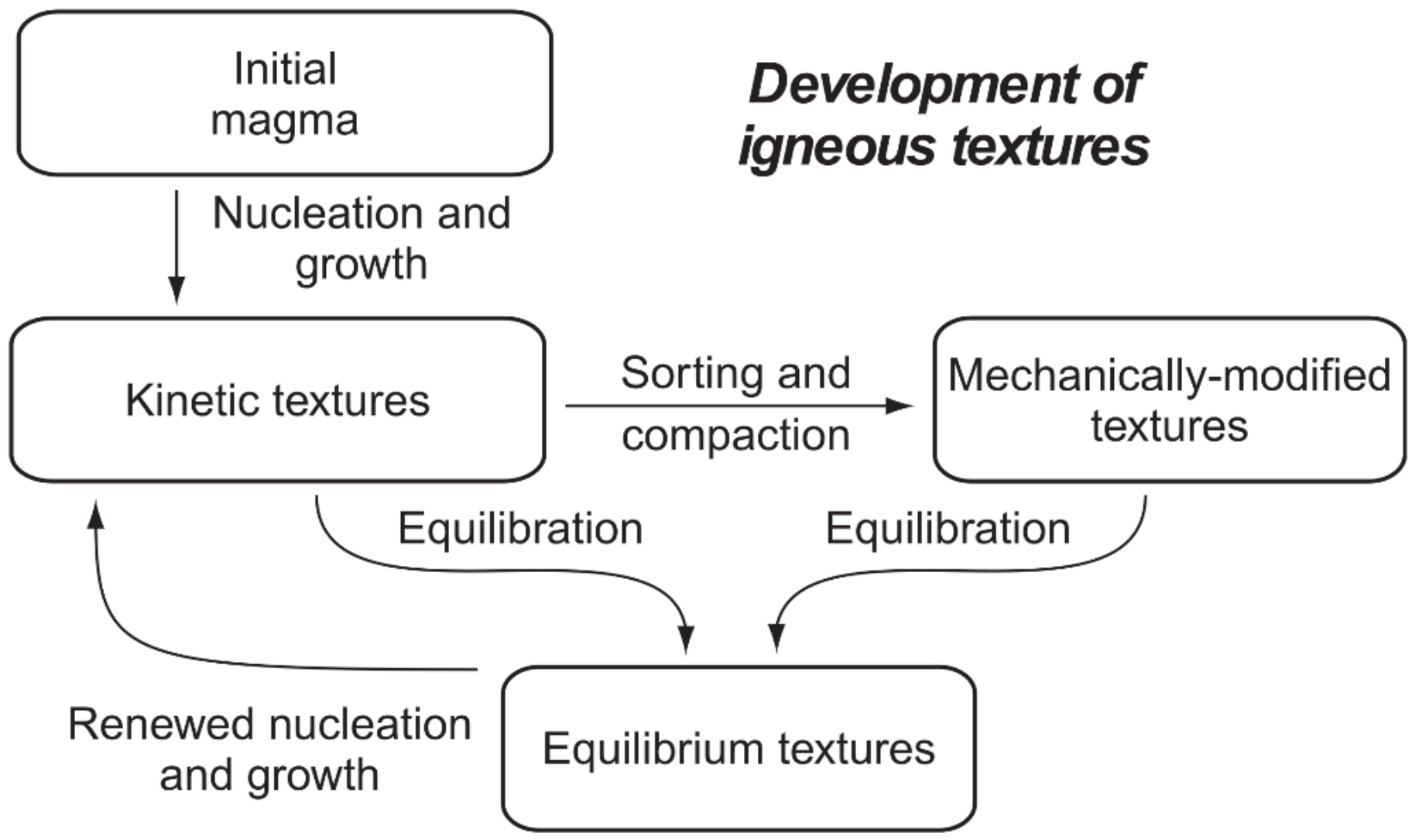

Supplementary Figure 8: Schematic representation of the steps for the development of igneous textures in terms of kinetic, mechanical, and equilibrium processes (see Higgins[4]).

Aiming to compare a natural, silicic cumulate system with the sphere-based model of compacting crystal mushes developed by Špillar and Dolejš[12], we produced a schematic model by considering the process of crystal-melt segregation in terms of the textural record. Such a model requires a consistent conceptualization, and involves (*i*) a system at a random stage of crystallization before reaching the first rheological locking at *ca.* 35-40 vol.% of crystals (*i.e.* touching framework) with logarithmic crystal size distribution (CSD) and random to clustered spatial distribution patterns (SDP); (*ii*) segregation might occur by gravitational mechanisms such as crystal settling and/or elutriation; (*iii*) nucleation continues in the "pores" between the cumulative large crystals, sustaining the main crystal framework; (*iv*) post-cumulus coarsening marks the final stages of the crystallization process (Supplementary Figure 9). The latter mechanism explains

the apparent increase in the number of smaller crystals in 2D sections compared to the 3D model. These 'new crystals' are segments of larger crystals that extend into the analyzed 2D section.

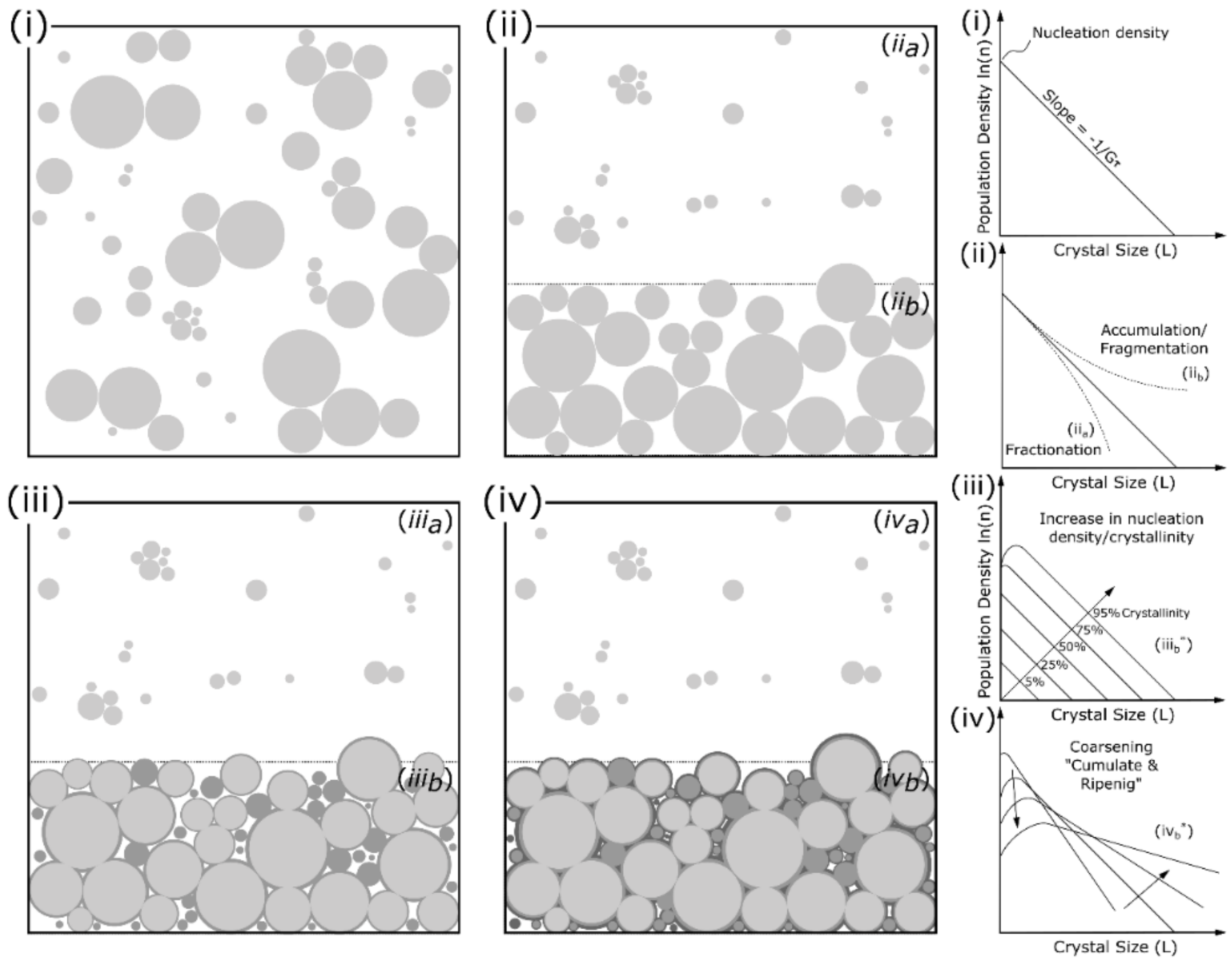

Supplementary Figure 9: Schematic representation of the textural evolution and associated CSDs resulting from crystal-melt segregation and post-cumulus coarsening.

To reconstruct how such an evolution would be reflected in the resulting texture, we simulate the textural evolution of a magmatic system (Supplementary Figure 10). In the proposed initial stage (*i*), *ca.* 30 vol.% of crystals within a magma suspension (diluted to semi-diluted regime following Frontoni *et al*.[13] constitute a clustered and non-touching crystal framework (porosity/non-crystals volume = 30 vol.%; R-value = 1.195) with log-normal CSD. To achieve a "natural-like" CSD and SDP corresponding to this stage, we used a natural syenitic sample with ~700 crystals and reduced the database to a total of 100, resulting in a CSD pattern with a similar slope but with a reduced intercept, *i.e.*, with a decreased apparent final nucleation rate. The initial magmatic suspension is then submitted to a segregation of the larger crystals and the residual, crystal-bearing interstitial liquid (*ii*). This process increases the overall sorting by keeping the smaller crystals in a suspension (ii_a), representing *ca.* 60 vol.% of the system, and accumulating the larger ones in the base of the system (ii_b), accounting for *ca.* 40 vol.%. The extracted liquid contains *ca.* 4.5 vol.% crystals at a clustered but non-touching framework (porosity = 95.5 vol.%; R-value = 0.970). Conversely, the cumulus counterpart is constituted by *ca.* 44.5 vol.% of crystals at a clustered and touching framework (porosity = 55.5 vol.%; R-value = 1.321). In the classical and modern studies of igneous rocks, most attention has been given to the "crystal-poor/magma-rich" part of the system, as it experiences the most significant magmatic processes. Instead, this conceptual model focuses on the textural evolution of the residual, crystal-rich cumulate.

After the formation of a rigid, touching crystal framework, the residual liquid can either crystallize new phases or promote the coarsening of existing ones (*iii*). In both cases, there will be an increase in the crystal population density in the 2D sections analyzed, accompanied by a corresponding increase in R-values.

The crystallization of the residual entrapped liquid can result in a maximum compaction configuration during continued solidification, and in the example, it occurs at 56.4 vol.% and R-value = 1.421 (porosity = 43.6 vol.%). Following the post-cumulus evolution, overgrowth/coarsening dominates the crystallization process (*iv*), which also includes newly nucleated crystals that in fact might apparently present as the rim of larger crystals occurring in the upper or lower position relative to the analyzed 2D section. This results in an increase in crystallinity that is not accompanied by compaction. The resulting cumulate will be constituted by *ca.* 83.2 vol.% of crystals exhibiting an R-value = 1.420 (porosity = 16.8 vol.%;), which represents a clustered crystal distribution in a touching framework.

As can be seen in Supplementary Figure 10, the model based on lognormal CSD reproduces the same pattern described by the Random Sphere Distribution Line (RSDL) in the R-value versus Porosity (vol.%) diagram[10] and straddles the fields for non-touching to touching crystal frameworks[9], except for a decrease in the overall sorting that diminishes the R-values. By comparing the model to the natural data from the Campo Alegre-Corupá volcano-plutonic system, we can assess the textural evolution of the whole system, assuming that the syenites represent rocks with mechanically modified textures. In contrast, the volcanic rocks represent the extracted residual liquids containing the smaller crystals in suspension that have been separated from the initial crystal-rich magma. Overgrowth/coarsening can account for as much as *ca.* 25 vol.% increase in the modal volume of the main mineral phase, suggesting that the granitic sample evolved from a non-touching to a touching framework and then equilibrated owing to overgrowth/coarsening of the main mineral phases. Note that these textural results are consistent with the topology of the composite SDP patterns, resulting in two nearly linear segments of '*compaction*' and '*crystal overgrowth*' (see main text).

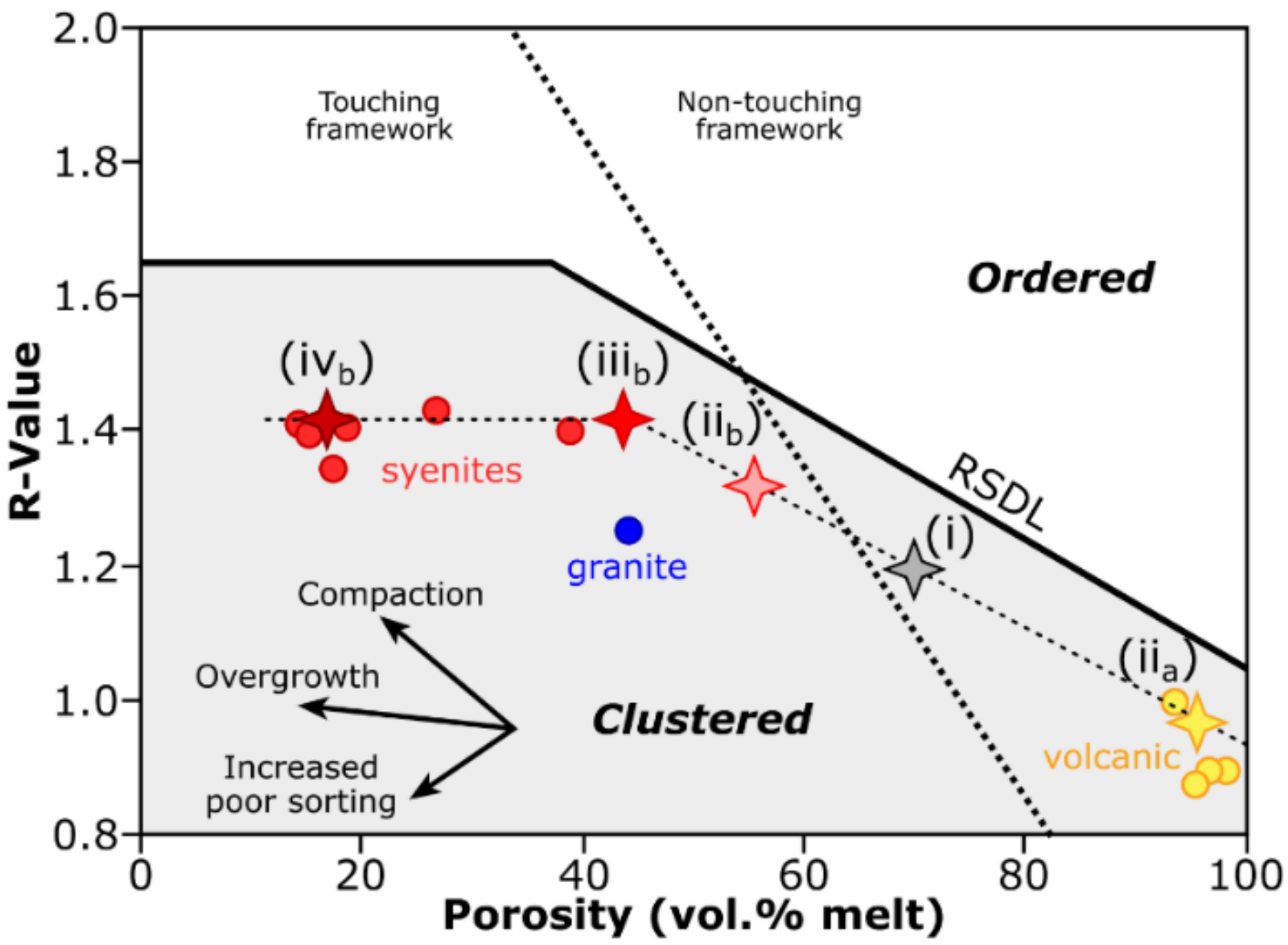

Supplementary Figure 10: Natural and synthetic evolution of igneous textures resulting from crystal-liquid segregation processes. (i) represents the initial state of a given magmatic suspension before reaching the rheological locking, which seems absent in the studied volcano-plutonic system, possibly owing to textural equilibration. (ii_a) and (ii_b) are, respectively, the complementary magma suspension and cumulate formed by crystal-melt segregation. Note the similar modal and textural correspondence between (ii_a) and the volcanic record. Finally, (iii_b) and (iv_b) represent the further steps of textural equilibration that the silicic cumulates undergo until reaching complete crystallization. The initial (iii_b) and final (iv_b) stages of textural equilibration share significant similarities with the syenites from the studied plutonic record.

4. Melt extraction percentages based on crystallinity-ordering relationships

Špillar and Dolejš[12] developed a 3D numerical model to investigate how melt extraction or crystal accumulation modifies igneous textures, and how these textural changes can be used to quantify when melt extraction occurred and how much melt was removed from a crystal mush. For this, they followed a *step-by-step* approach, first creating realistic crystal mushes by simulating *in situ* crystallization using the VoxelTex code, which generates 3D crystal populations with straight log-linear CSDs, forming realistic crystal aggregates and crystal suspensions of known initial crystallinity (Φ_i). These textures represent magmas that crystallize without the extraction of interstitial melt, being controlled solely by crystallization kinetics (Supplementary Figure 11a). The next step is to "freeze" crystallization and compact the suspension/mush. Crystal growth and nucleation are halted, and the existing crystals and aggregates are progressively packed into smaller volumes (*e.g.*, 10 vol.%; Supplementary Figure 11b), which is mathematically equivalent to extracting interstitial melt. This process is implemented using a Monte Carlo packing algorithm, independent of the specific physical mechanism involved (*e.g.*, crystal settling, mush compaction, filter-pressing, etc.).

Using this approach, the authors track how textures evolve during melt extraction. At each compaction step, they measure the crystallinity (Φ) and the clustering index (R-values), which quantifies the degree of spatial ordering or clustering of crystal centers in 2D sections extracted from the 3D models (Supplementary Figure 11b). They measure such variations in 2D sections with the aim of calibrating the relationship between Φ and R-values for the application to conventional quantitative textural analysis of igneous rocks. In the model, melt-extraction systematically increases crystallinity, enhances spatial ordering (higher R-values), and produces textures distinct from those generated by simple *in situ* static crystallization (*Random Texture Trend - RTT*, Supplementary Figure 11c), as expected. They further demonstrate a universal relationship between R-values and Φ during compaction. For textures with straight CSDs, this relationship is independent of CSD slope and absolute crystal size. This universality allows the construction of calibration curves linking Φ and R-values to the initial crystallinity and the Melt Extraction Percentage (MEP; Supplementary Figure 11d). By comparing these curves with measured Φ and R-values, it is possible to infer when melt extraction began, how much melt was removed, and whether the system behaved as open to mechanical extraction or nearly closed.

Importantly, the rheological implications of their method include the prediction of two percolation thresholds, which is consistent with classical models of igneous petrology (see Vigneresse *et al.*[14]; Frontoni *et al.*[13], and references therein). The first one, at ~35 vol.% crystals for *in situ* crystallization, marks the formation of a rigid framework, whereas the second, up to ~65-75 vol.% crystals, defines compacted mushes starting from low Φ_i. These results highlight that mush rheology strongly depends on crystal spatial organization, not solely on crystal fraction. In this sense, the method provides a quantitative, texture-based tool to diagnose melt extraction in fossil magma chambers using only crystallinity and crystal spatial ordering. This is particularly useful in systems in which geochemical signatures become ambiguous, especially in crystal-rich, multiply saturated systems such as silicic occurrences. However, the application of this method remains limited to occurrences containing phenocrysts, as it cannot predict crystal accumulation/melt-extraction for samples submitted to textural maturation during post-cumulus crystallization. In our manuscript, we present an alternative approach to standard detailed textural characterization, which excludes overgrown rims and late interstitial crystals (see Fiedrich *et al.*[15]), by using CSDs and SPDs.

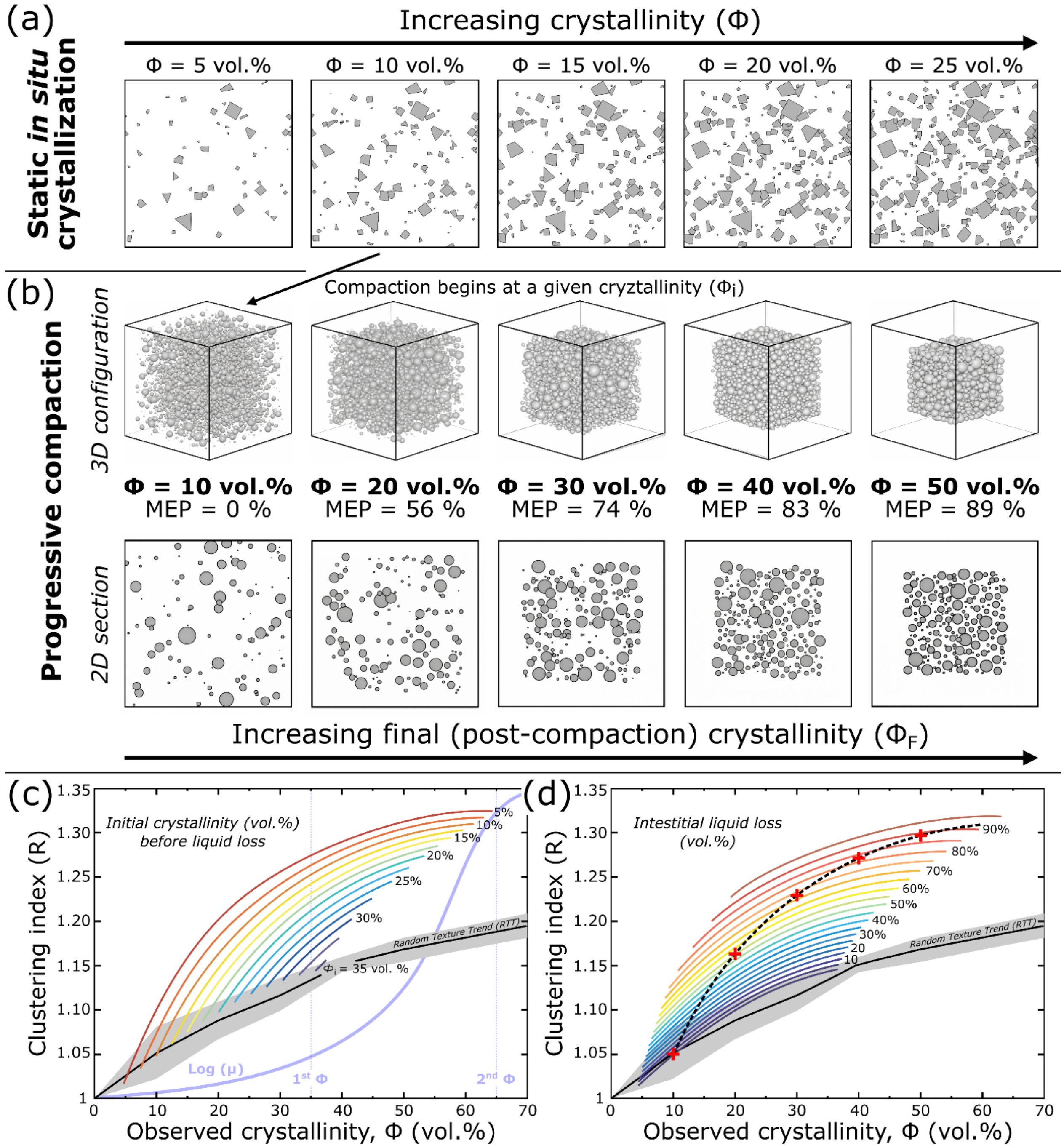

Supplementary Figure 11: (a) Schematic evolution of textures submitted to *in situ*, static crystallization using the VoxelTex code of Špillar and Dolejš[12]. (b) When the system reaches a given crystallinity (in this example 10 vol.%), three-dimensional numerical compaction occurs in discrete steps until the rheological locking and crystallinity-ordering relationships are measured from 2D sections obtained. (c-d) Evolutive patterns for increasing compaction based on initial crystallinity and the percentage of interstitial liquid loss (Melt Extraction Percentage - MEP) are then estimated, resulting in isolines derived from empirical polynomial fits (Modified after Špillar and Dolejš[12]). The light-blue line in (c) schematically illustrates the relative variation of viscosity (Log μ) as a function of crystallinity after Frontoni *et al.*[13], evidencing the first and second percolation thresholds of magma viscosity. For values of $\Phi < \sim 35$ vol.%, the rheological regime exhibits a Newtonian (diluted and semi-diluted regimes) behavior, and beyond this threshold, it exhibits a non-Newtonian (concentrated regime) behavior, until a plateau-like viscosity region at high crystal fraction is attained for values of $\Phi > \sim 65$ vol.%, at which point the magma exhibits a non-Newtonian (hyper-concentrated regime) behavior. The dashed-black line in (d) shows the increase in ordering (R-values) and crystallinity resulting from the melt-extraction of a crystal suspension with 10 vol.% solids submitted to compaction, as illustrated in (b). The red crosses mark the initial stage, at 10 vol.% and MEP = 0, followed by crystallinities of 20, 30, 40, and 50 vol.%, for MEPs of 56, 74, 83, and 89%, respectively.

5. Deduction of the elutriation velocity based on a critical particle size

We define the elutriation velocity (V_{elut}) as the relative velocity between solid particles settling under gravity and an upward-moving fluid. Thus, V_{elut} corresponds to the difference between the settling (sedimentation) velocity of particles (V_{set}) and the upward fluid velocity (V_{flu}), both defined in the same reference frame. For a given upward fluid flux, there exists a critical particle diameter (d_0) for which V_{set} = V_{elut}, resulting in V_{elut} = 0 and dynamic suspension of the particle in place. Under these assumptions, the elutriation velocity can be written as:

$$V_{elut} = V_{set} - V_{flu} \qquad \textit{(Eq.5.1)}$$

For solid particles smaller than the critical diameter d_0 to which $V_{set} = V_{flu}$, the ascending fluid flux will be able to carry them upwards (Supplementary Figure 12a). Conversely, there is no motion for particles with sizes equal to the critical diameter (Supplementary Figure 12b), whereas they can settle if their sizes exceed the critical diameter (Supplementary Figure 12c) with velocities computed by *Eq.5.1*.

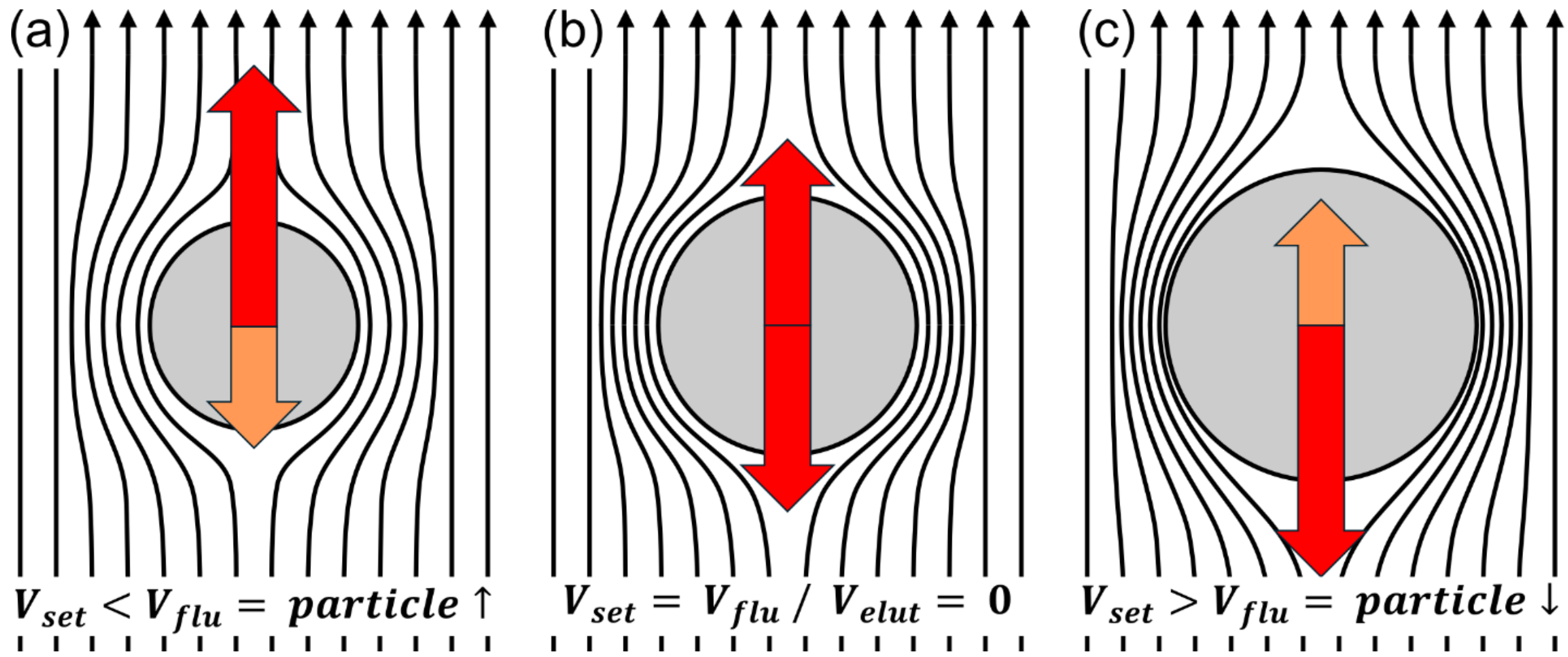

Supplementary Figure 12: Examples in which the upwards-moving fluid flux exhibits velocities that are (a) greater, (b) equal, or (c) smaller than the settling velocities of solid particles.

Under diluted conditions (crystal fraction << 0.1), the settling velocities of solid particles V_{set} can be computed using Stokes' Law:

$$V_{set} = \frac{g(\rho_{crystal} - \rho_{melt})d^2}{18\mu} \qquad \textit{(Eq.5.2)}$$

The upward fluid velocity V_{flu} is defined here as the velocity required to keep a particle of critical diameter d_0 in dynamic equilibrium (*i.e.*, zero net vertical motion). Accordingly, V_{flu} can be expressed as the Stokes settling velocity of a particle with diameter d_0:

$$V_{flu} = \frac{g(\rho_{crystal} - \rho_{melt})d_0^2}{18\mu} \qquad \textit{(Eq5.3)}$$

where g is the gravitational force, $\rho_{crystal}$ and ρ_{melt} are the densities of crystals and melt, d is the particle diameter, and μ is the dynamic viscosity of the fluid. By substituting *Eq.5.2* and *Eq.5.3* into *Eq.5.1*, the elutriation velocity can be expressed as:

$$V_{elut} = \frac{g(\rho_{crystal} - \rho_{melt})d^2}{18\mu} - \frac{g(\rho_{crystal} - \rho_{melt})d_0^2}{18\mu} \qquad \textit{(Eq.5.4)}$$

This equation represents the complete form of the elutriation velocity as a function of particle diameter relative to a critical particle size d_0. As defined above, the upward fluid velocity corresponds to the settling velocity of the critical particle size, such that:

$$V_{flu} = V_{set} * \frac{d_0^2}{d^2} \quad (Eq.5.5)$$

This relation emphasizes that V_{flu} is uniquely determined by the condition of dynamic equilibrium imposed on particles of diameter d_0. Thus, as the V_{flu} depends on the diameter of a critical solid particle, the V_{elut} equation (*Eq.5.1*) can be written in a more elegant form, which is based on the V_{set} obtained for arbitrary diameters relative to the critical diameter d_0:

$$V_{elut} = \left[V_{set} * \left(1 - \frac{d_0^2}{d^2}\right)\right] \quad (Eq.5.6)$$

This equation results in a profile of absolute V_{elut} values as a function of particle sizes, exhibiting a rapid decrease in V_{elut} ('*sinkhole*') for diameters progressively equal to d_0, for which $V_{elut} = 0$ (Supplementary Figure 13). This formulation highlights the existence of a critical particle size for which the relative velocity is zero, separating upward transport from settling regimes. Solid particles with diameters approaching d_0 exhibit elutriation velocities that tend to zero, whereas smaller and larger particles experience upward and downward motion, respectively.

When compared with the results of settling velocities obtained solely through Stokes' Law, particles controlled by the mechanism of elutriation will exhibit contrasting directions of movement. These changes in the sense of motion result in velocities of settling that are still similar to those obtained through Stokes's Law for particle sizes larger than the critical d_0 value. However, the velocities of the upward-moving crystals are up to three orders of magnitude greater (Supplementary Figure 13a). Note that the comparison between elutriation and settling velocities changes when the mechanism of hindered settling (or hindered movement) is considered for solid-rich suspensions ($c > 0.1$) rather than highly diluted ($c << 0.1$), as discussed in the main text. Furthermore, the resulting equation *Eq.5.6* has a strong dependence on the velocity of an ascending magma flux, as supported by the assumption of a critical particle size d_0 for which $V_{set} = V_{flu}$. Thus, changing the velocity of the upward-moving magma flux can exert control on which crystal sizes will be carried up and/or settle. More importantly, it changes the particle size that will remain static and defines a range of dimensions that will move slowly either up or down. As seen in Supplementary Figure 13a, variations in the velocity of the upwards-moving magma flux for values corresponding to the settling velocities of particles with d = 5, 10, and 15 mm will change the position at which absolute values will tend to zero, as expected. In the example, variations in the values of crystal velocities are more expressive for particles smaller than the critical size. The resulting equation *Eq.5.6* can also be explored for other magma compositions, which rely upon the difference between densities of solids and the liquid phase ($\rho_{crystal}$ and ρ_{melt}), and it is ultimately controlled by their dynamic viscosities (μ). Supplementary Figure 13b demonstrates how magma viscosity of rhyolitic and basaltic magmas will influence the mechanisms of crystal settling and elutriation, and as expected, velocities for basalts are four orders of magnitude greater because viscosities are four orders of magnitude smaller when compared with rhyolites.

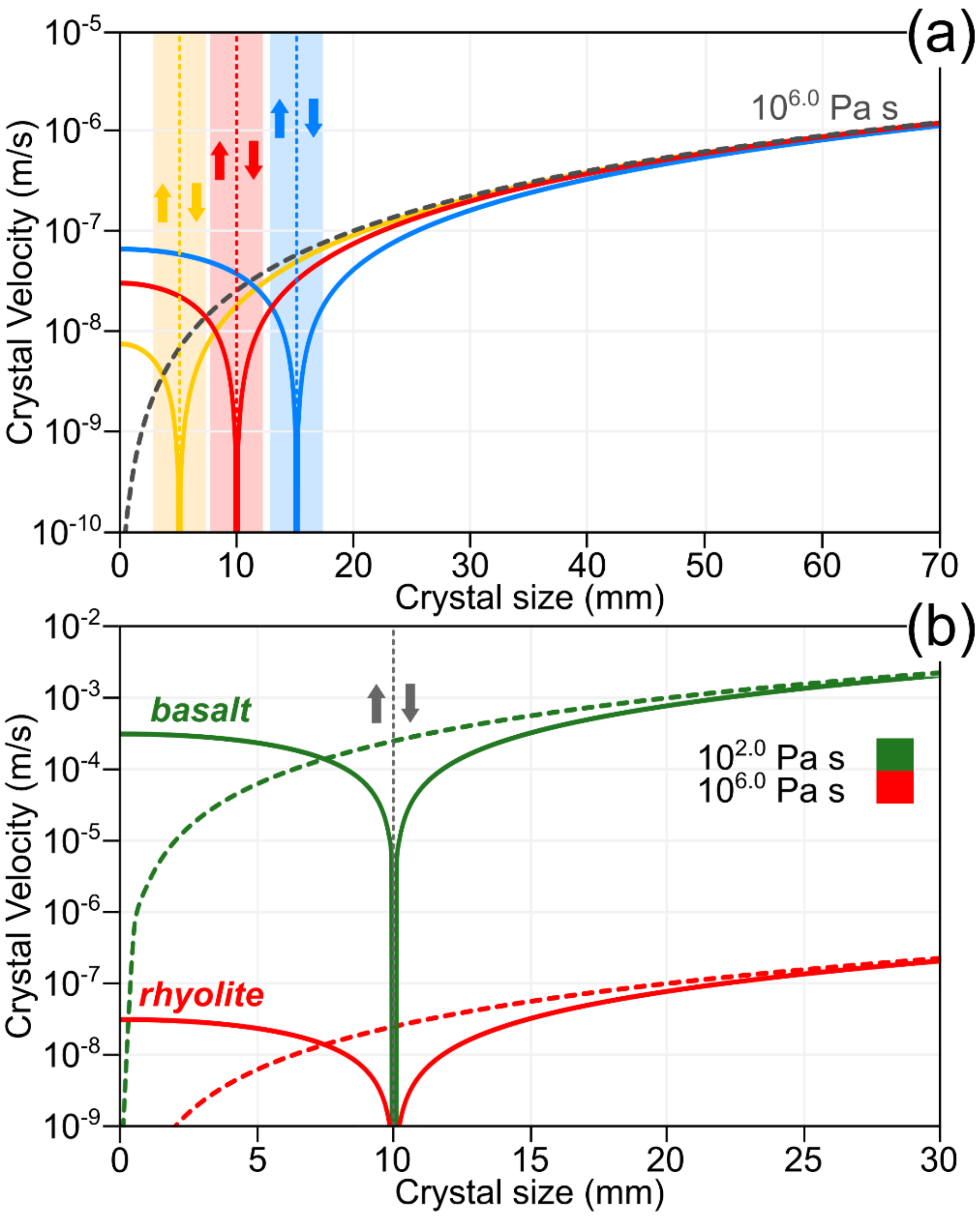

Supplementary Figure 13: (a) Examples of absolute values of settling (V_{set}, dashed lines) and elutriation (V_{elut}, continuous lines) crystal velocities (m/s) computed based on Stokes' Law and considering an upward-moving magma flux in equilibrium with a critical particle size d_0 = 5, 10, and 15 mm as a function of crystal sizes. (b) Examples of absolute values of V_{set} and V_{elut} crystal velocities computed for contrasting values of magma viscosity.

6. Supplementary 8-bit processed images used for CSD analysis

Supplementary 8-bit black-and-white images used for quantitative textural analysis of alkali feldspar crystals obtained from volcanic and plutonic samples from the Campo Alegre-Corupá Volcanic and Igneous Plumbing System. The rectangular sections were obtained from conventional and large thin-sections (see **Methods**) exhibiting irregular boundaries, following best practices to compensate for edge effects. The red scale bars correspond to one centimeter.

Sample BCA-2023-P23 - early erupted porphyritic trachyte

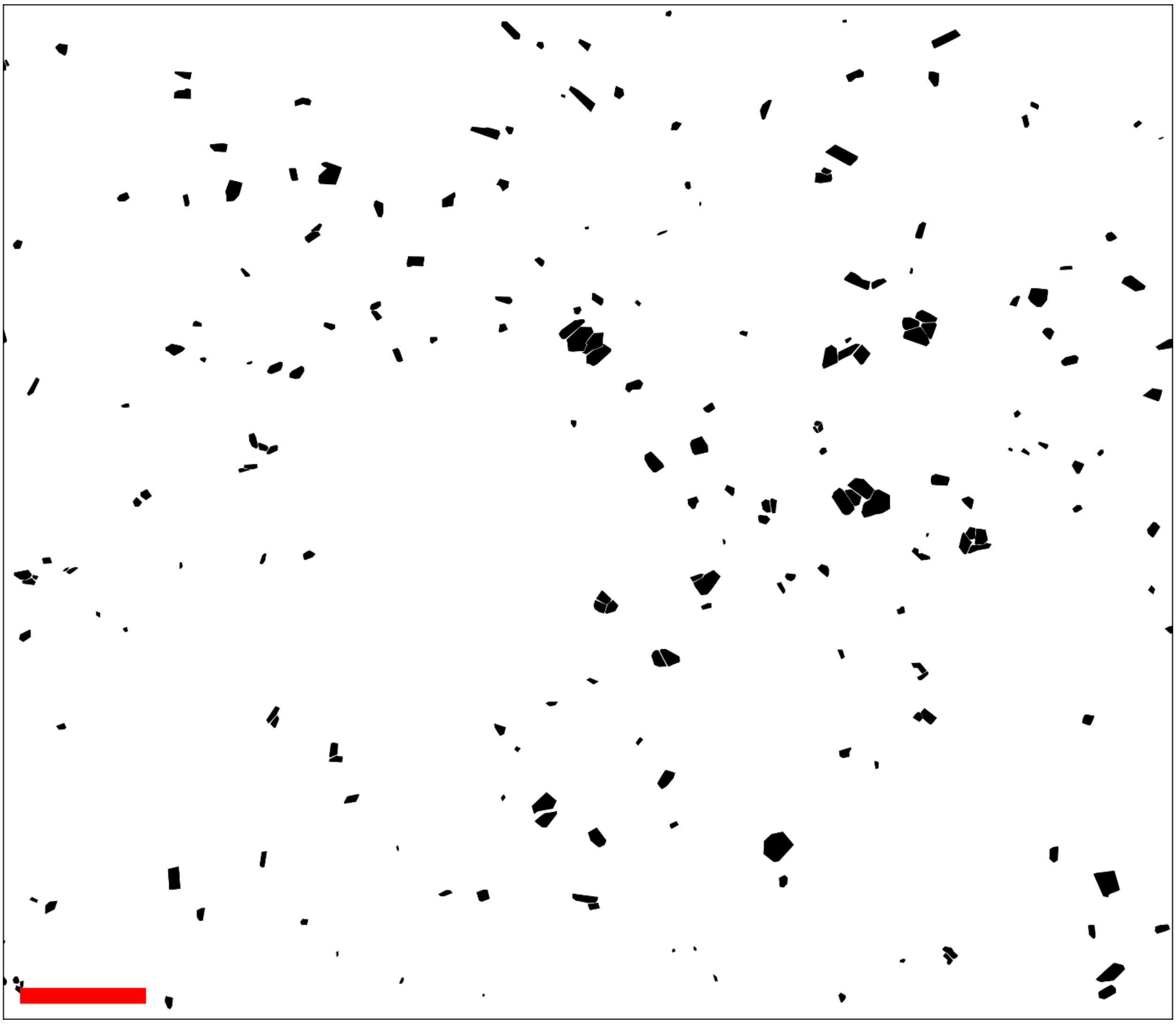

Sample BCA-2023-P18 - subvolcanic rhyolitic dike

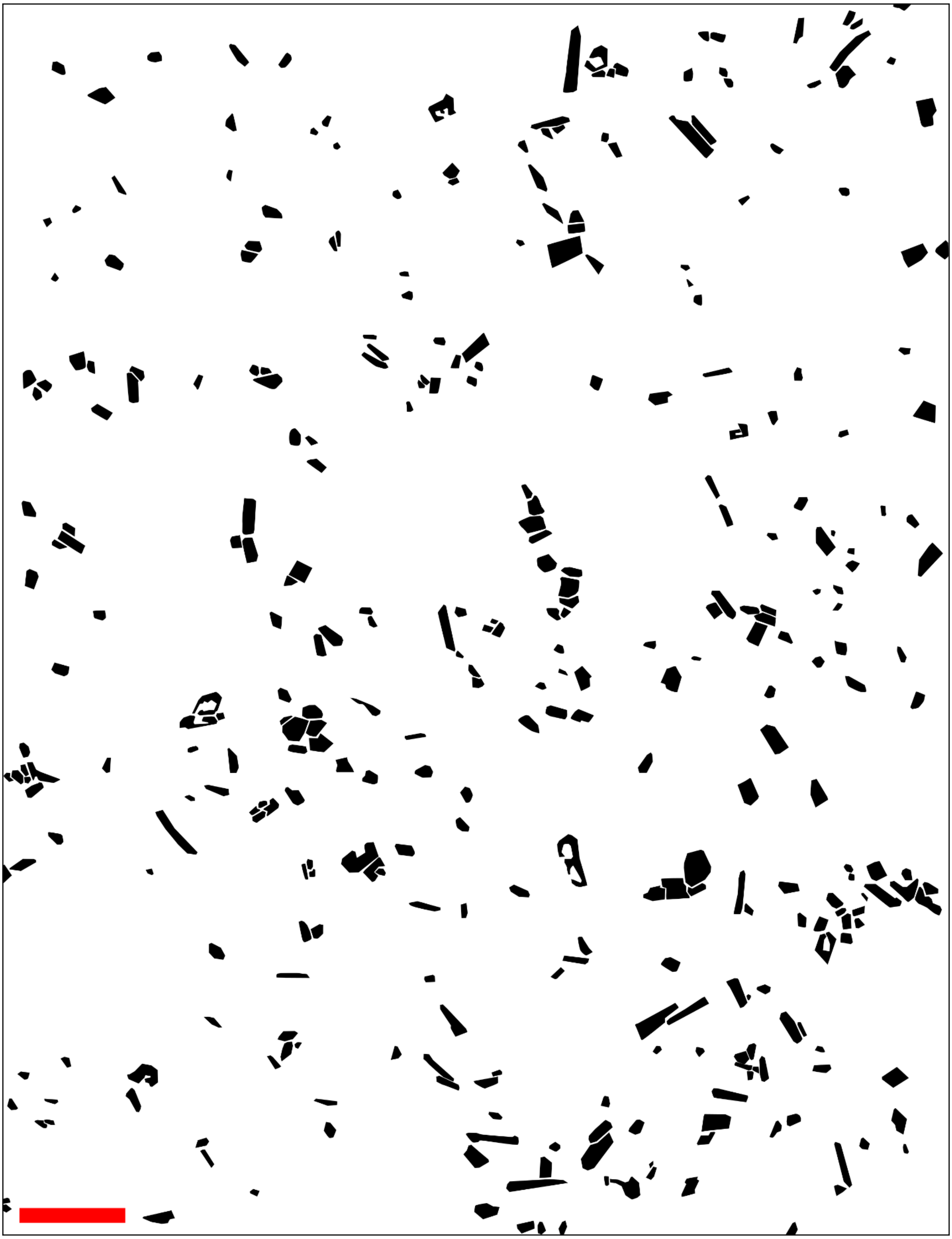

Sample CO-2a - medium-grained granite

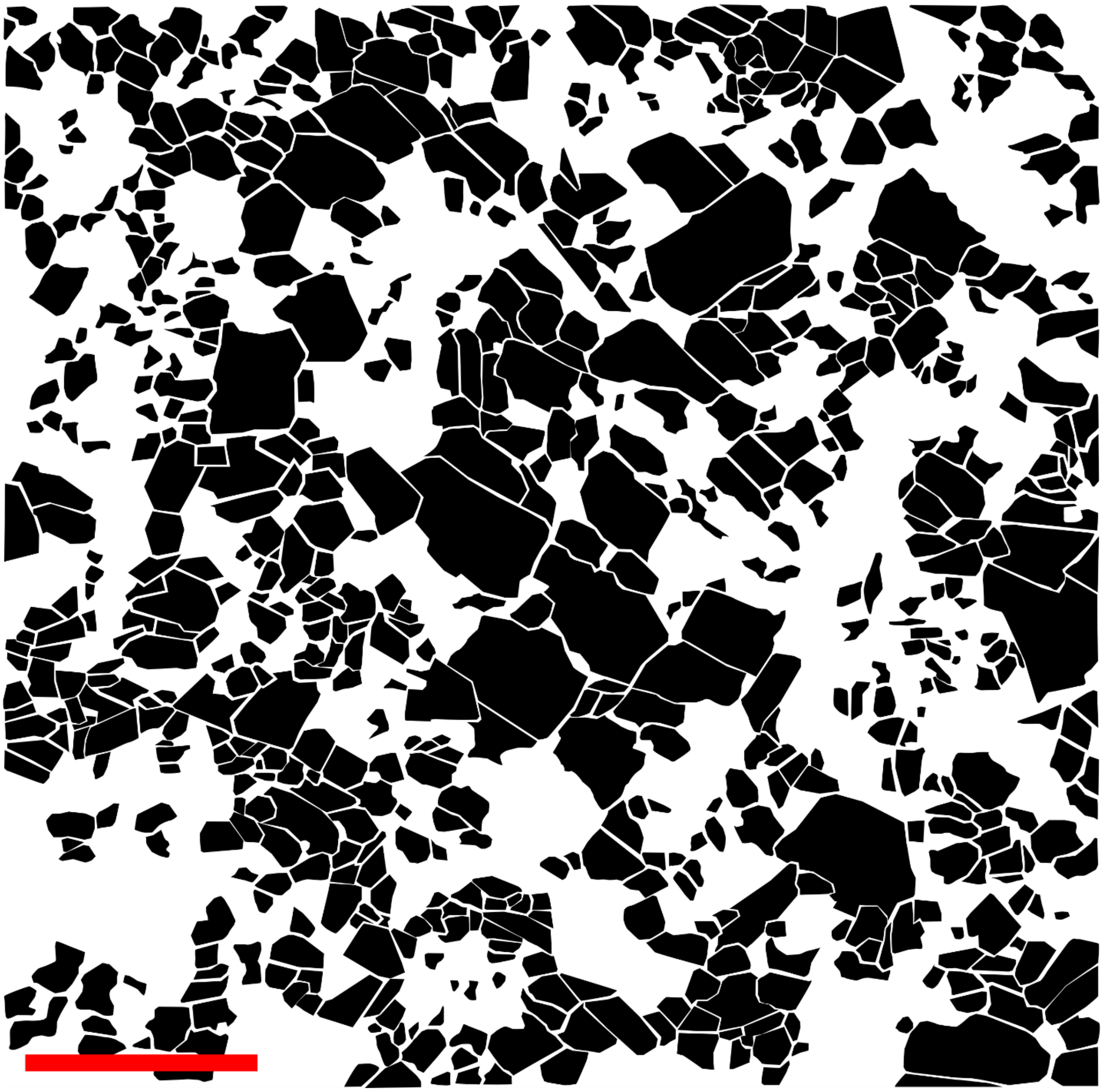

Sample CO-53 - coarse-grained syenite from pink facies

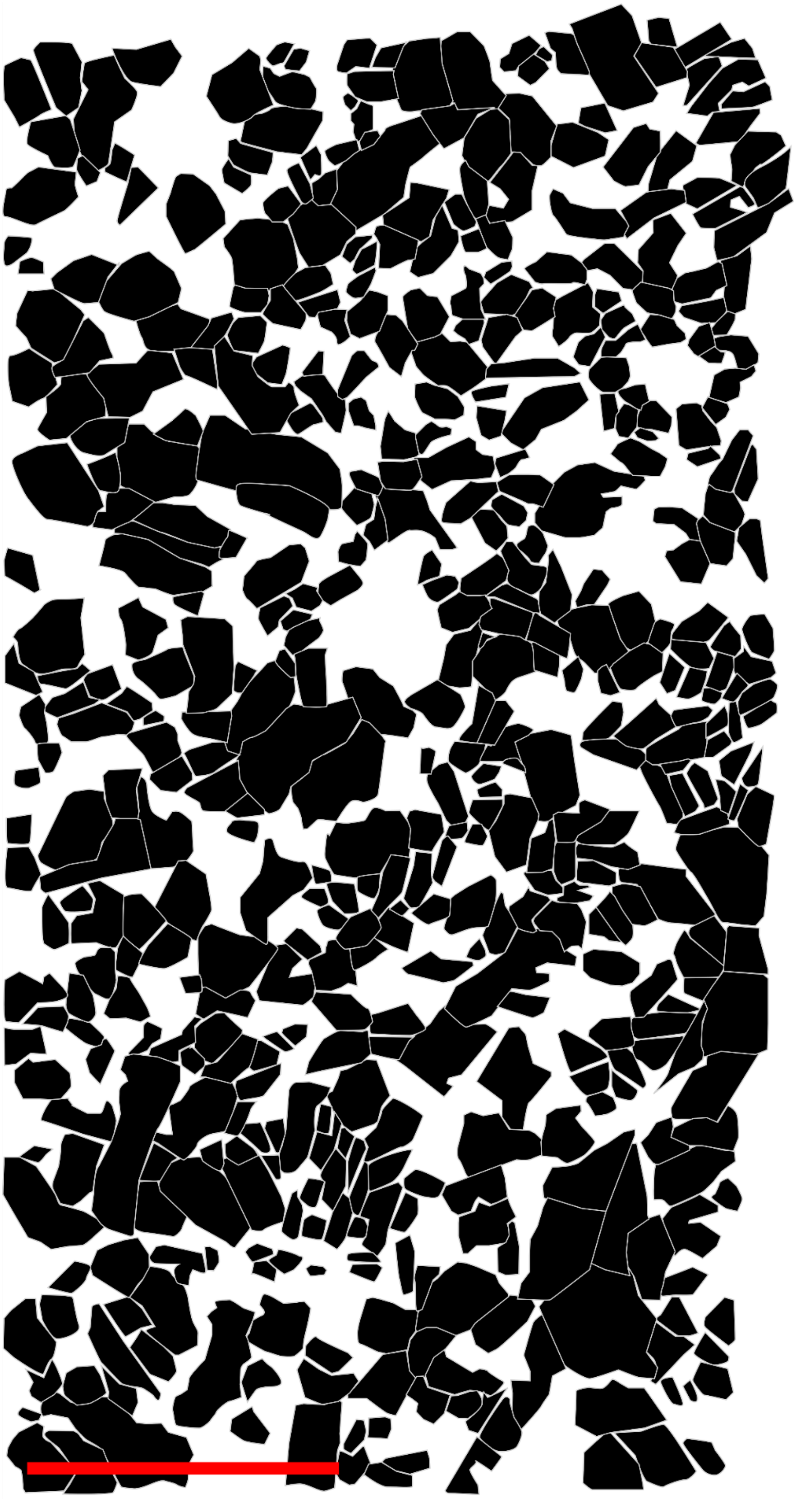

Sample CO-38 - medium-grained syenite from pink facies

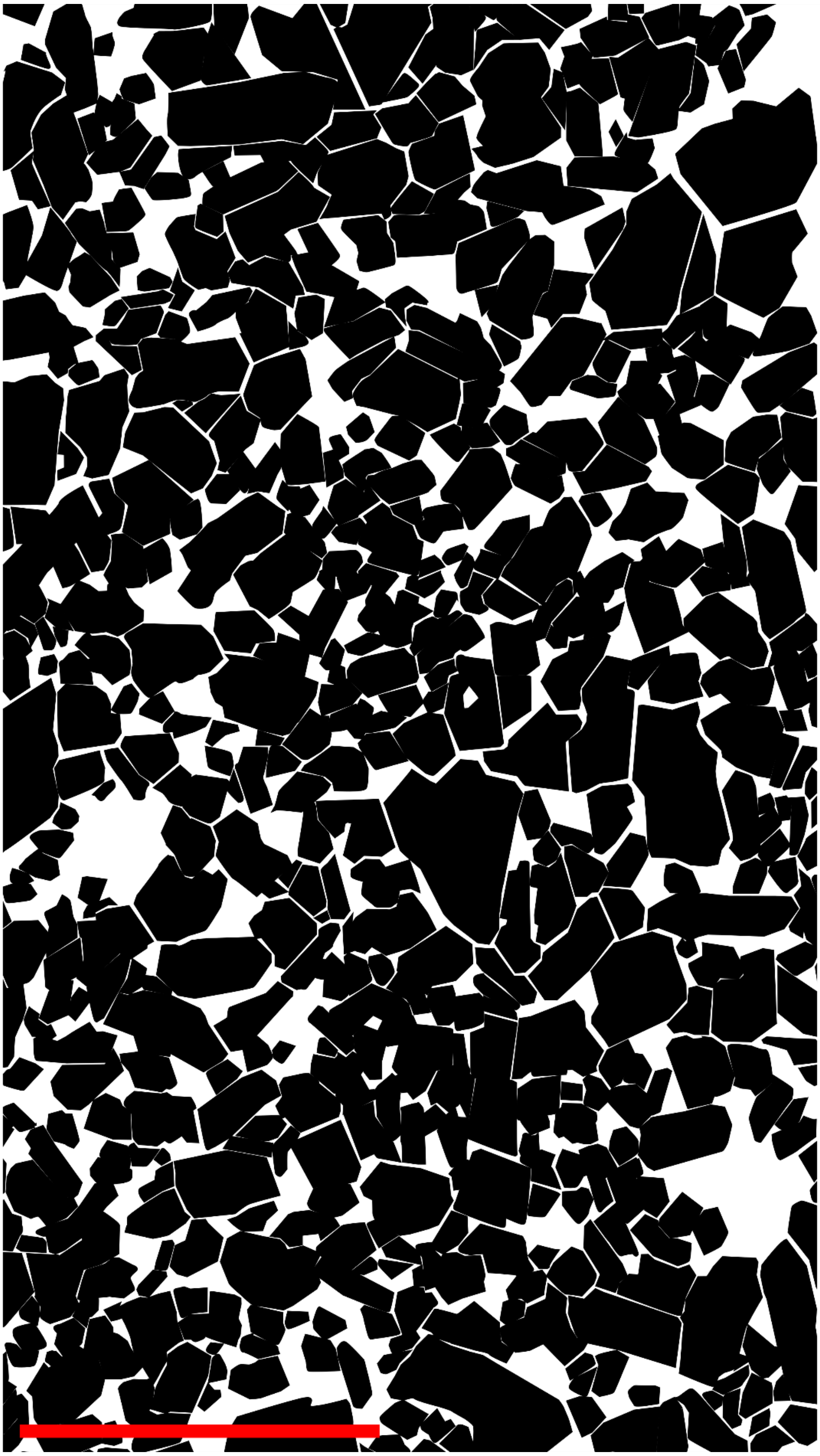

Sample CO-40b - very coarse-grained syenite from pink facies

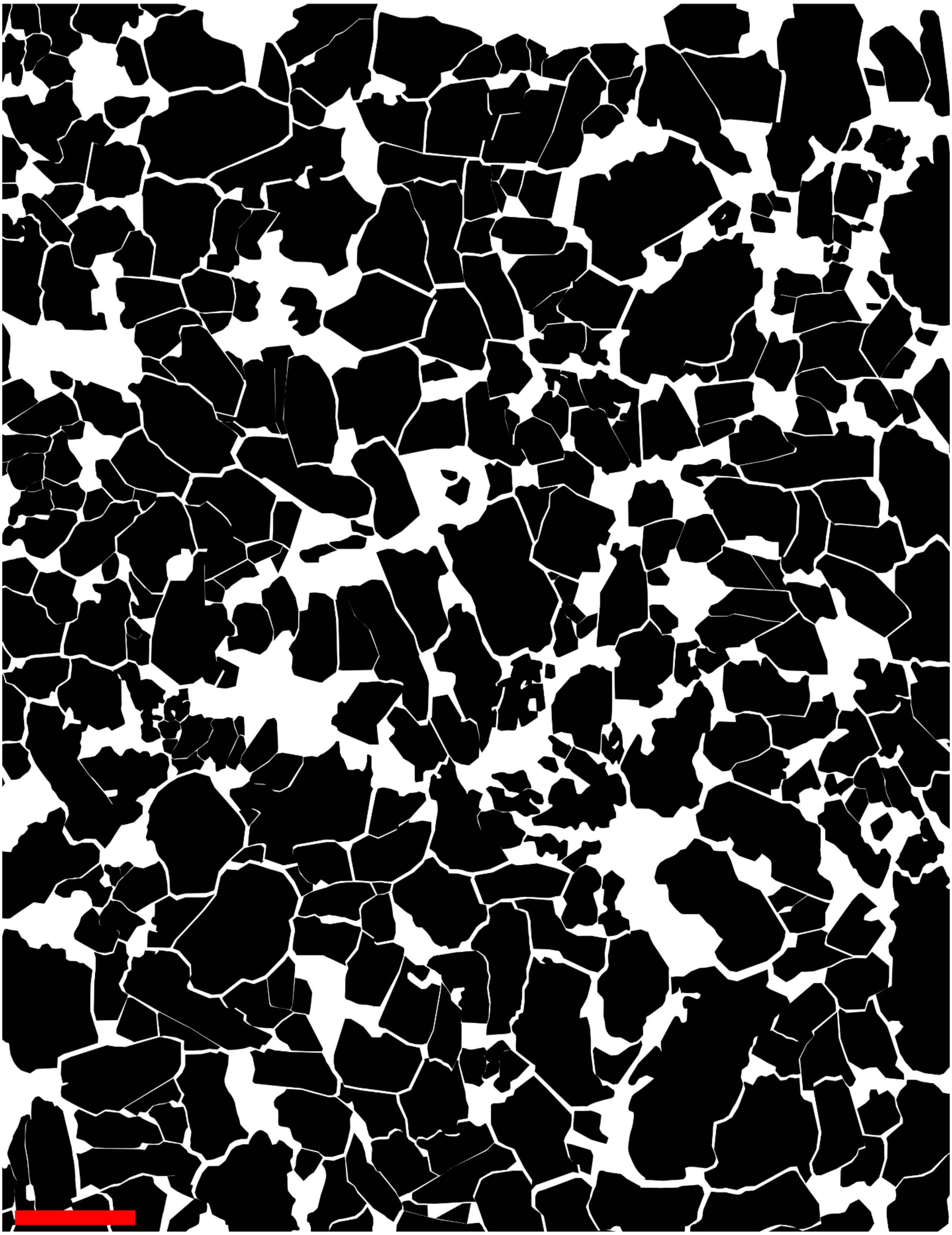

Sample CO-4a - coarse-grained syenite from pink facies

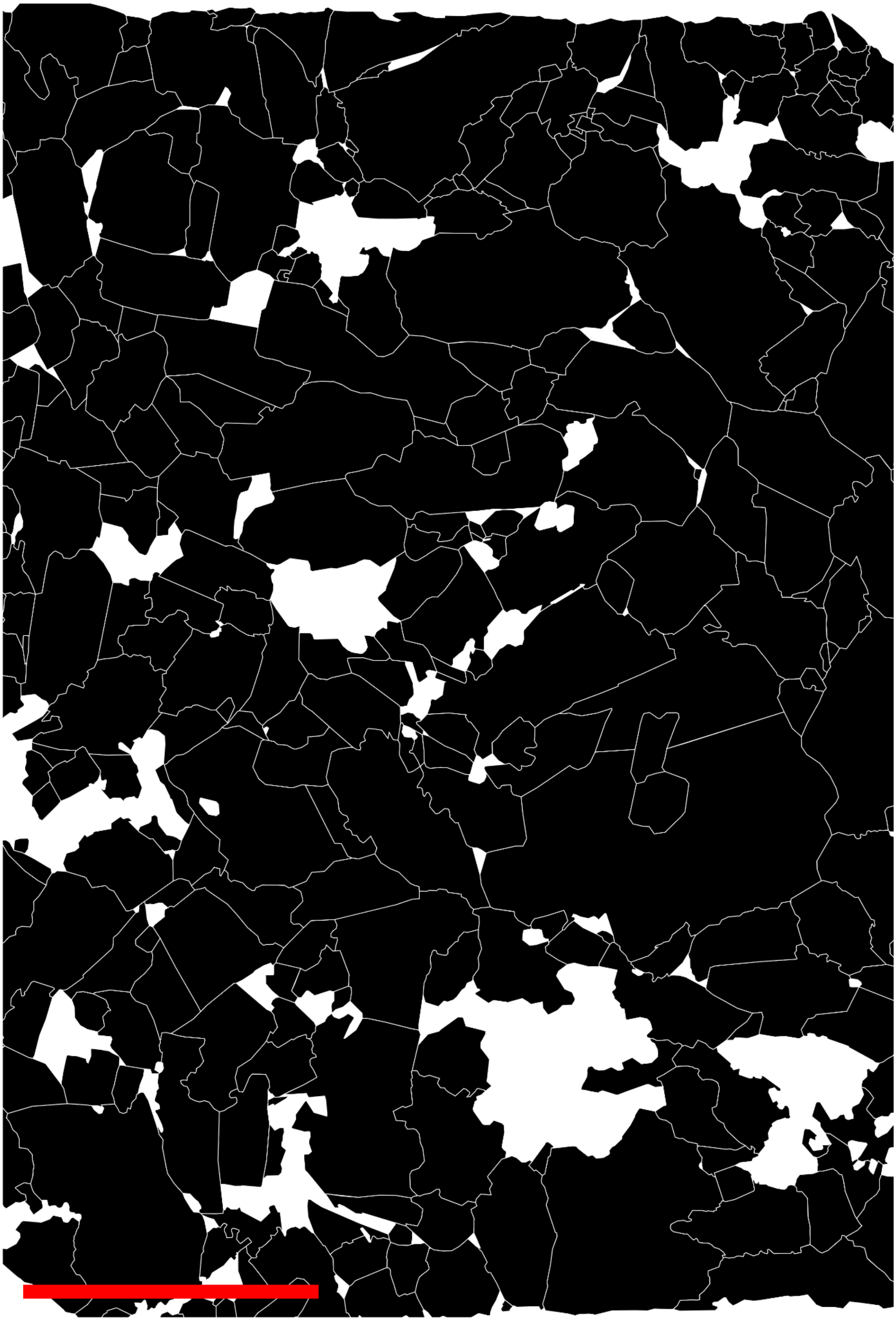

Sample CO-4c - coarse-grained syenite from green facies

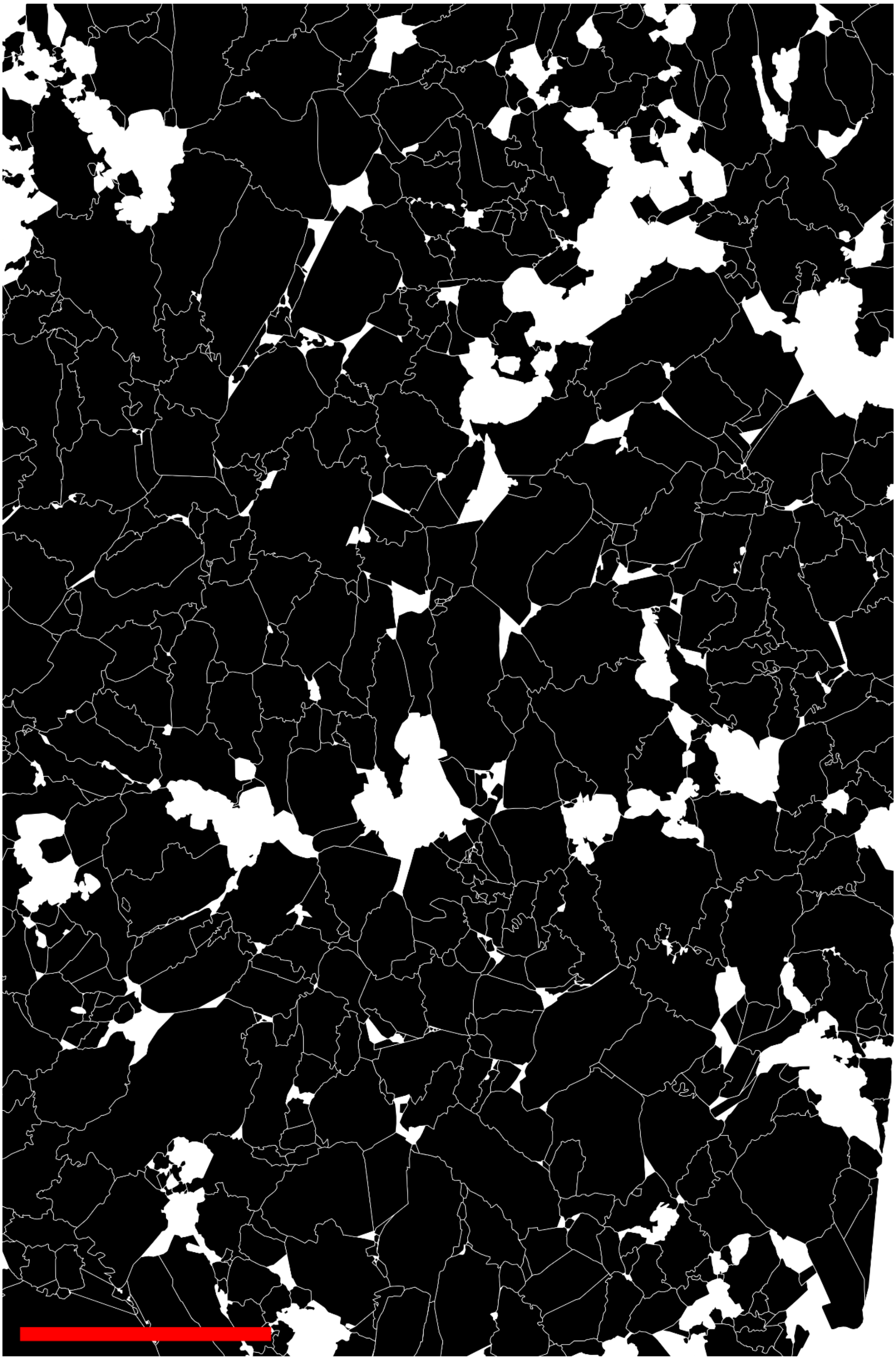

Sample S8 - very coarse-grained syenite from pink facies

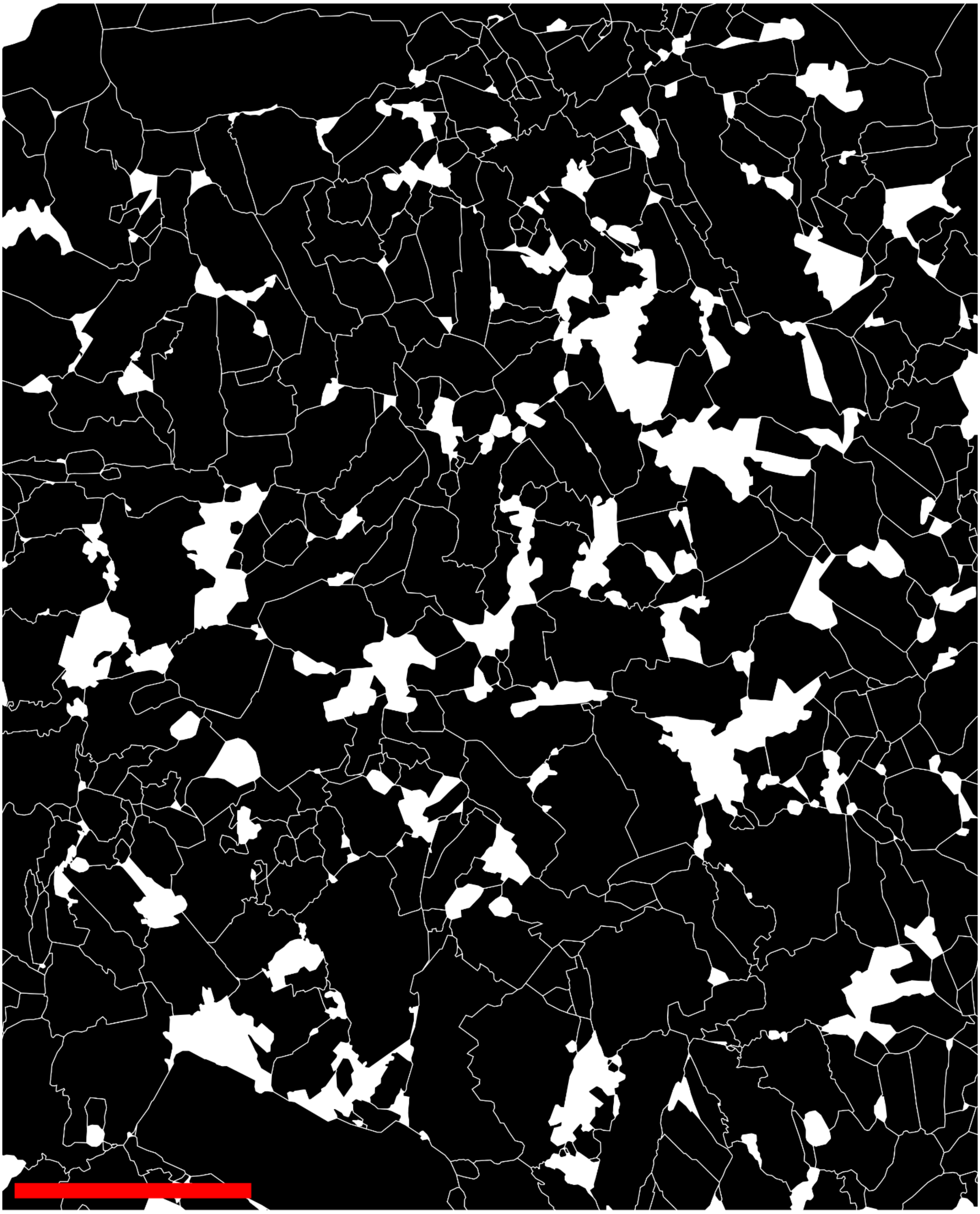

Supplementary Tables to 'Enhanced crystal-melt segregation within dynamic mush systems links silicic cumulates with caldera-forming eruptions'

Supplementary Table 1: Crystal size distribution parameters obtained for the studied samples from the Campo Alegre-Corupá Volcanic and Igneous Plumbing System (CAC-VIPS)

Sample*	#Fsp	Vol.%	n^0 (±2σ)	C_L (±2σ)	S (±2σ)	r^2	L_{Max} (mm)	R-value (±2σ)	AR (±2σ)	AF
					Volcanic Samples					
**BCA-2019-P23[1]**	296	4.7	-2.61 (0.19)	0.532 (0.033)	-1.879 (0.110)	0.998	3.75	0.896 (60)	1:1.7(0.1):2(0.1)	0.15
**CAL-IV-40[2]**	472	8.3	-3.26 (0.12)	1.256 (0.056)	-0.796 (0.034)	0.999	6.38	1.001 (48)	1:1.4(0.2):2.8(0.7)	0.23
**BCA-2019-P18[3]**	446	6.4	-3.65 (0.13)	1.006 (0.043)	-0.994 (0.041)	0.998	6.64	0.879 (48)	1:1.6(0.1):2.1(0.5)	0.20
**PPW-01[4]**	755	5.2	-2.98 (0.09)	0.875 (0.032)	-1.142 (0.040)	0.988	7.91	0.898 (40)	1:1.2(0.1):1.8(0.1)	0.29
**BCA-2019-P22[5]**	353	5.7	-5.74 (0.22)	1.894 (0.159)	-0.528 (0.041)	0.986	11.81	0.867 (56)	1:1.9(0.1):2.8(0.7)	0.24
					Plutonic Samples					
**CO-2a[6]**	478	55.7	0.33 (0.16)	0.698 (0.030)	-1.433 (0.060)	0.999	6.40	1.253 (48)	1:1.5(0.2):1.9(0.2)	0.09
**CO-4a[7]**	512	82.4	-3.32 (0.12)	2.409 (0.201)	-0.415 (0.032)	0.994	13.17	1.348 (46)	1:1.4(0.1):2.3(0.2)	0.06
**CO-4c[8]**	742	84.8	-2.74 (0.09)	2.298 (0.076)	-0.435 (0.014)	0.999	16.03	1.396 (38)	1:1.4(0.1):2.8(0.5)	0.13
**CO-38[9]**	967	73.3	-1.19 (0.08)	1.169 (0.035)	-0.855 (0.025)	0.999	8.76	1.435 (34)	1:1.5(0.1):2.6(0.2)	0.07
**CO-40b[10]**	411	81.4	-5.58 (0.14)	3.246 (0.154)	-0.308 (0.014)	0.999	25.50	1.409 (52)	1:1.5(0.2):1.9(0.2)	0.13
**CO-53[11]**	688	61.2	-1.83 (0.10)	1.492 (0.060)	-0.670 (0.026)	0.976	13.74	1.402 (40)	1:1.4(0.1):2.4(0.2)	0.23
**S8[12]**	431	85.8	-3.96 (0.12)	3.115 (0.142)	-0.321 (0.014)	0.997	25.40	1.414 (50)	1:1.5(0.1):3(0.1)	0.12

Supplementary Table 1 (Continuation): Crystal size distribution parameters obtained for the studied samples from the Campo Alegre-Corupá Volcanic and Igneous Plumbing System (CAC-VIPS)

Sample*	L (mm)	*ln n** (mm^{-4})	*ln* max n*	*ln* min n*	Vol. (%)	R-value
			Volcanic Samples			
**BCA-2019-P23[1]**	3.75	-9.67	-9.34	-10.15	-	-
	2.37	-7.09	-6.93	-7.27	-	-
	1.49	-5.27	-5.16	-5.38	-	-
	0.94	-4.48	-4.37	-4.62	-	-
	0.59	-5.25	-4.92	-5.79	-	-
**CAL-IV-40[2]**	6.38	-8.33	-8.19	-8.52	-	-
	4.03	-6.48	-6.39	-6.60	-	-
	2.54	-5.26	-5.18	-5.36	-	-
	1.60	-4.54	-4.44	-4.65	-	-
	1.01	-4.68	-4.48	-4.90	-	-
**BCA-2019-P18[3]**	6.64	-10.21	-9.99	-10.51	-	-
	4.19	-7.95	-7.83	-8.10	-	-
	2.64	-6.22	-6.14	-6.32	-	-
	1.67	-5.31	-5.23	-5.41	-	-
	1.05	-6.25	-5.90	-6.62	-	-
**PPW-01[4]**	7.91	-11.7	-11.32	-12.31	-	-
	4.99	-9.16	-8.97	-9.39	-	-
	3.15	-6.73	-6.64	-6.83	-	-
	1.99	-5.19	-5.12	-5.26	-	-
	1.25	-4.14	-4.07	-4.21	-	-
	0.79	-4.47	-4.32	-4.65	-	-
**BCA-2019-P22[5]**	11.81	-11.82	-11.51	-12.29	-	-
	7.45	-9.97	-9.76	-10.24	-	-
	4.70	-8.33	-8.17	-8.51	-	-
	2.97	-7.09	-6.95	-7.24	-	-
	1.87	-7.25	-6.99	-7.59	-	-

Sample*	L (mm)	*ln n** (mm^{-4})	*ln* max n*	*ln* min n*	Vol. (%)	R-value
			Plutonic Samples			
	13.74	-10.6	-10.20	-11.30	17.83	1.016
	8.67	-8.20	-7.98	-8.47	29.36	1.190
	5.47	-5.96	-5.84	-6.09	45.43	1.253
**CO-2a[6]**	3.45	-4.00	-3.92	-4.07	53.63	1.240
	2.18	-2.83	-2.77	-2.90	55.52	1.250
	1.37	-3.13	-2.99	-3.31	55.70	1.241
	0.87	-4.18	-3.76	-4.93	-	-
	13.17	-8.78	-8.54	-9.09	10.54	0.978
	8.31	-6.84	-6.69	-7.02	21.26	1.206
	5.24	-5.54	-5.41	-5.69	48.19	1.352
**CO-4a[7]**	3.31	-4.42	-4.30	-4.55	68.56	1.388
	2.09	-4.19	-4.00	-4.40	79.10	1.369
	1.32	-4.04	-3.76	-4.42	82.43	1.348
	0.83	-3.65	-3.30	-4.26	-	-
	0.52	-3.92	-3.27	-∞	-	-
	16.03	-9.70	-9.44	-10.06	9.48	0.975
	10.11	-7.12	-6.99	-7.26	38.37	1.249
	6.38	-5.61	-5.51	-5.71	65.53	1.411
	4.03	-4.50	-4.41	-4.60	79.02	1.396
	2.54	-3.92	-3.81	-4.04	82.93	1.355
**CO-4c[8]**	1.60	-3.32	-3.19	-3.47	84.82	1.396
	1.01	-2.54	-2.40	-2.70	-	-
	0.64	-2.04	-1.86	-2.26	-	-
	0.40	-1.64	-1.41	-1.95	-	-
	0.25	-1.22	-0.93	-1.62	-	-
	0.16	-1.46	-0.97	-2.45	-	-
	8.76	-8.62	-8.39	-8.90	25.34	1.122
	5.53	-6.09	-5.97	-6.20	32.74	1.240
	3.49	-4.11	-4.04	-4.18	54.31	1.413
**CO-38[9]**	2.20	-3.07	-3.00	-3.13	66.75	1.435
	1.39	-2.37	-2.29	-2.44	72.50	1.397
	0.87	-3.47	-3.20	-3.90	73.30	1.435
	0.55	-3.94	-3.51	-4.69	-	-
	25.50	-13.48	-13.40	-15.17	20.20	1.057
	16.10	-10.38	-10.09	-10.45	33.00	1.161
	10.15	-8.66	-8.42	-8.66	35.48	1.221
**CO-40b[10]**	6.40	-7.51	-7.20	-7.61	58.77	1.391
	4.04	-6.85	-6.54	-6.90	74.76	1.409
	2.55	-7.11	-6.38	-7.39	81.40	1.409
	1.61	-7.76	-6.97	-∞	-	-
	6.40	-8.89	-8.03	-9.35	20.73	1.091
	4.04	-5.36	-5.19	-5.50	23.67	1.173
**CO-53[11]**	2.55	-3.31	-3.21	-3.39	44.02	1.333
	1.61	-2.03	-1.95	-2.10	57.57	1.402
	1.02	-2.14	-1.99	-2.29	61.20	1.397
	25.40	-12.01	-11.61	-12.71	6.66	1.038
	16.03	-9.36	-9.17	-9.60	21.99	1.244
	10.11	-7.09	-6.99	-7.21	54.56	1.371
**S8[12]**	6.38	-5.89	-5.80	-6.00	75.87	1.414
	4.03	-5.38	-5.26	-5.51	82.50	1.402
	2.54	-4.73	-4.59	-4.88	85.78	1.414
	1.60	-5.02	-4.73	-5.38	-	-

Volcanic Samples:** 1) early erupted trachyte, 2) late erupted trachyte, 3) subvolcanic rhyolitic dike, 4) syn-eruptive densely welded lapilli tuff, 5) plagioclase from a transitional basalt. ***Plutonic Samples:** 6) medium-grained granite, 7) coarse-grained syenite from pink facies, 8) coarse-grained syenite from green facies, 9) medium-grained syenite from pink facies, 10) very coarse-grained syenite from pink facies, 11) coarse-grained syenite from pink facies, 12) very coarse-grained syenite from pink facies. **#Fsp**: Number of alkali feldspar or plagioclase crystals analyzed. **Vol.%**: modal abundance of alkali feldspar or plagioclase crystals; $\mathbf{n^0}$: population density (Y-intercept); $\mathbf{C_L}$: characteristic length (-1/S); **S**: slope of CSD lines; $\mathbf{L_{Max}}$: maximum crystal length; **R-value**: clustering index; **AR**: aspect ratio (small: intermediate: large crystal axis, S: I: L); **AF**: alignment factor (varying from 0 to 1 for isotropic to completely aligned textures). **L (mm):** Crystal sizes measured in millimeters and organized as size-bins; ***ln n** ($\mathbf{mm^{-4}}$):** Population densities of crystals at a given size (mm) per volume (mm^{-3}); ***ln max n* and ***ln min n*****:** Values of population densities computed for positive and negative uncertainties, respectively; **Vol.%**: modal abundance of feldspar crystals for each respective size-bin; **R-value:** Clustering indexes measured for incremental including size-bins starting from the larger size-populations.

Supplementary Table 2: Bulk-rock compositions of trachydacitic samples from the *Campo Alegre-Corupá Basin* used in the thermodynamic modeling at 200 MPa.

Sample ID	SiO_2	Al_2O_3	Fe_2O_{3Tot}	MnO	MgO	CaO	Na_2O	K_2O	TiO_2	P_2O_5	LOI	Sum
BE-02	67.63	14.66	5.36	0.03	0.28	0.92	4.54	4.60	0.83	0.41	1.49	100.75
BE-05	69.66	14.21	4.67	0.05	0.08	0.32	4.35	5.36	0.41	0.13	0.73	99.97

REFERENCES

1. Randolph, A. D., and Larson, M. A. *Theory of Particle Processes* (New York: Academic Press, 1971).
2. Marsh, B. D. Crystal size distribution (CSD) in rocks and the kinetics and dynamics of crystallization: Theory. *Contributions to Mineralogy and Petrology* **99**, 277-291 (1988).
3. Cashman, K. V., and Marsh, B. D. Crystal size distribution (CSD) in rocks and the kinetics and dynamics of crystallization II: Makaopuhi Lava Lake. *Contributions to Mineralogy and Petrology* **99**, 292-305 (1988).
4. Higgins, M. D. *Quantitative textural measurements in igneous and metamorphic petrology* (Cambridge university press, 2006).
5. Higgins, M. D. Measurement of crystal size distributions. *American Mineralogist* **85**, 1105-1116 (2000).
6. Marsh, B. D. On the interpretation of crystal size distributions in magmatic systems. Journal of Petrology **39**, 553-599 (1998).
7. Burkhart, L., Hoyt, R. C., and Oolman, T. *Control of particle size distribution and agglomeration in continuous precipitators* (Sintering Processes, 1980).
8. Higgins, M. D. Magma dynamics beneath Kameni volcano, Thera, Greece, as revealed by crystal size and shape measurements. *Journal of Volcanology and Geothermal Research* **70**, 37-48 (1996).
9. Jerram, D. A., Cheadle, M. J., and Philpotts, A. R. Quantifying the building blocks of igneous rocks: are clustered crystal frameworks the foundation?. *Journal of Petrology* **44**, 2033-2051 (2003).
10. Jerram, D. A., Cheadle, M. J., Hunter, R. H., and Elliott, M. T. The spatial distribution of grains and crystals in rocks. *Contributions to Mineralogy and Petrology* **125**, 60-74 (1996).
11. Clark, P. J., and Evans, F. C. Distance to nearest neighbor as a measure of spatial relationships in populations. *Ecology* **35**, 445-453 (1954).
12. Špillar, V., and Dolejš, D. Melt extraction from crystal mushes: numerical model of texture evolution and calibration of crystallinity-ordering relationships. *Lithos* **239**, 19-32 (2015).
13. Frontoni, A., Costa, A., Vona, A., and Romano, C. A comprehensive database of crystal-bearing magmas for the calibration of a rheological model. *Scientific Data* **9**, 247 (2022).
14. Vigneresse, J. L., Barbey, P., and Cuney, M. Rheological transitions during partial melting and crystallization with application to felsic magma segregation and transfer. *Journal of Petrology* **37**, 1579-1600 (1996).
15. Fiedrich, A. M., Bachmann, O., Ulmer, P., Deering, C. D., Kunze, K., and Leuthold, J. Mineralogical, geochemical, and textural indicators of crystal accumulation in the Adamello Batholith (Northern Italy). *American Mineralogist* **102**, 2467-2483 (2017).
16. Lino, L. M., Quiroz-Valle, F. R., Louro, V., Basei, M. A. S., Vlach, S. R. F., Hueck, M., Montecinos Muñoz, P. R., & Citroni, S. B. Structural architecture and the episodic evolution of the Ediacaran Campo Alegre Basin (southern Brazil): Implications for the development of a synorogenic foreland rift and a post-collisional caldera-volcano. *Journal of South American Earth Sciences* **107**, 103147 (2021).
