# Supplementary material for: Enhanced crystal-melt segregation within dynamic mush systems links silicic cumulates with caldera-forming eruptions
Source: Nat Commun. 2026 Jun 27;17:8060. doi: 10.1038/s41467-026-74812-4 (PMC13454170; doi:10.1038/s41467-026-74812-4)
Supplement: Supplementary file 2 [file 41467_2026_74812_MOESM2_ESM.pdf]

nature portfolio

Peer Review File

Enhanced crystal-melt segregation within dynamic mush systems links silicic cumulates with caldera-forming eruptions

Corresponding Author: Dr Lucas Lino

Parts of this Peer Review File have been redacted as indicated to remove third-party material.

This file contains all reviewer reports in order by version, followed by all author rebuttals in order by version.

Version 0:

Reviewer comments:

Reviewer #2

(Remarks to the Author)
I am pleased to provide my assessment of this manuscript, drawing on my expertise in igneous petrology, plutonic–volcanic connections, and quantitative petrography. From the outset, I would like to emphasize that the manuscript is clearly written, well organized, and appropriately contextualized within the current literature. The authors make effective use of recent work, and the results are presented in a way that is accessible to a broad readership. I recommend that this manuscript be accepted for publication in Nature Communications after minor revisions addressing the points outlined below.

The study emphasizes the dynamic nature of magmatic systems, in contrast to previous static models. By applying quantitative petrography techniques, particularly the analysis of crystal size distributions (CSD), the authors provide new insights into the mush paradigm. The manuscript contributes to the ongoing debate regarding the volcanic–plutonic connection and the elusive nature of silicic cumulates, issues also highlighted in recent studies such as Clemens et al. (2022). The originality and potential impact of this work are evident, and I believe it represents a meaningful step forward in our understanding of crustal magmatic processes. In addition, the study provides valuable evidence supporting the connection between rhyolites and silicic intrusive rocks, an issue that is rarely documented in detail and remains the subject of ongoing debate.

The methods are robust, based on established approaches but applied here in a novel and systematic way that emphasizes the dynamic character of mush systems. The quality of the petrographic data and its presentation are adequate for supporting the conclusions. One limitation lies in the CSD analyses of volcanic rocks, where some crystals show evidence of dissolution or partial regrowth. While this complicates interpretation, the authors mitigate the issue by focusing primarily on plutonic samples. This limitation does not undermine the manuscript's overall conclusions.

In general, the conclusions are well supported by the data. The key inference that compaction occurs at lower crystallinities derives directly from the petrographic analyses. However, the claim that the system must have assembled rapidly (lines 396–397) is less convincing. The connection between this conclusion and the presented data is not clearly articulated, and additional explanation or supporting evidence would strengthen the argument.

The most suggested improvements:
• Clarify the reasoning behind the inference of rapid system assembly.
• Provide brief additional explanation in the text regarding how some parameters are interpreted, to help readers follow the method more easily.
• Discuss briefly the implications of dissolution and partial growth in volcanic crystals for the robustness of the CSD results.

For further clarity, I have also attached a PDF of the manuscript with detailed comments and suggestions marked directly in the text.

In conclusion, this is a clear, original, and significant study that contributes to our understanding of magmatic mush systems

and the origin of high-silica rhyolites. With minor revisions, the manuscript will fully meet the standards of Nature Communications and will be of broad interest to the community.

Sincerely.

Reviewer #3

(Remarks to the Author)
This manuscript titled "'Mush in Motion: How dynamic crystal-melt segregation links silicic cumulates with caldera-forming eruptions" by Lino et al., aims to re-evaluate the mush model and introduce a dynamic mush model. The case study is Campo Alegre- Corupá system (Brazil). The study combines textural analysis with thermodynamic modelling. The main finding of the paper is that volumes of high SiO2 magma can be generated with low crystallinities via a dynamic reservoir where crystals are elutriated, creating crystal clusters which rapidly sink.
While this is a significant piece of work and I do think the findings and implications are interesting, I have come concerns about the many assumptions used that are not properly addressed. My major concerns are as follows (see line by line comments for further details):
- Utilizing 2D textural data for CSD, while a common technique, is one that comes with the major assumption that the 2D sections are an accurate representation of the 3D crystals. This is not addressed in the MS at all and there is no error presented with the measurements of the crystal axes.
- The SDP calculations make several assumptions that are not addressed (e.g. variable crystal growth rate, continued crystal growth of large crystals during growth of small crystals, etc.) and the manual correction of these measurements is inadequately explained.
- The modelling crystal settling speeds/timescales, while really cool and I think something that will be useful to many readers, only works with a major assumption of the critical crystal size. While this choice is rooted in natural samples, again, there are many assumptions that come with this decision (e.g. no crystal growth after separation from the original melt, it is actually the largest crystal found, etc). This model cannot be correct as at different viscosities we would expect the critical size to change. While of course models require some degree of simplification, the assumptions must be clearly stated. I suggest an additional supplementary figure that compares the findings for a variable critical size should be added to show how much their results deviated when changing this critical parameter. Furthermore, it was very unclear to me how this equation was derived, and I think this is important to have in the methods or supplementary materials.
- Finally, the way the paper is written I don't think balances stressing the interest to a broad scientific audience and explaining what was done very well. The introduction does not make it clear the larger implications of this research question; this is truly not revealed until the end of the discussion. Furthermore, it took several reads of the methods/supplementary materials for the main manuscript to make sense, so I think the MS could benefit from some significant re-writes for clarity or a change to a longer format article.

Line by line Comments:
Line 19: The opening line cites what you call a "central topic in petrology" yet you only cite papers first authored by Olivier Bachmann.
Line 29: I found this sentence difficult to read… perhaps because of the tense of this sentence? Maybe change to "have often experienced" or "often experience".
Line 37: Would be great to see some advances you have mentioned to include 3D work that many folks are doing cited here (e.g. Jerram and Higgins 2017 which you later cite).
Line 56-62: This section I got a bit confused with when came when relative to each other. The word "early" is used a couple times, but it is unclear these are both the same age and not? A stratigraphic column on the side of Fig 1 might help. Alternatively, if available some ages for the different units may help? If the units have names, then I think this might also help to clarify what you are talking about and when. I understand you have tried to simplify it to make it easier for the reader, but since time is a very important part of your discussion, I think it is valuable to include it.
Line 67-71: The different facies (green and pink) are introduced here while explaining the mineralogy which I found abrupt. Maybe add an intro sentence/statement that explains the syenites have different facies? Or average the two facies and present that?
Fig 2: Since this figure is used for the CSD it would be useful to have one or two of the maps showing how where what you chose as the measurements for a few crystals. Especially since the chemistry of these maps is not discussed in detail so I assume you included this figure as it is what you used for the CSD. Additionally, could you add a scale bar to make these maps quantitative?
Line 104: A basaltic flow? Fall?
Line 107-108: I cannot tell which one is kinked – the lines are a bit too thick, so it is hard to see which is connected to which. Maybe consider making them thinner or changing the shade slightly (e.g. warm tones = syenites, cool = granite)? It might be helpful to indicate where you think the kink is? As it stands, I am not convinced that there is a kink.
Line 111: Do you mean Ostwald ripening?
Line 112: What do you mean by "hump shaped distributions?"
Line 117: The right side of this equation seems wrong. There is a close bracket that doesn't have an open bracket. Maybe one of the "/" should be "(" ?
Line 119: How are this r distance chosen? Are you doing this? Is there a model that you are using that does this? It is unclear from what you have written (including the methods and supplementary) how this is chosen.
Line 124-128: CSD calculations comes with major assumptions that you can extrapolate 3D data from 2D data. This is not addressed at all here and should be.
Figure 3:

- I found the use of the term Porosity in this figure and the rest of the manuscript to be extremely confusing. I understand that Jerram et al 1996 use this terminology but porosity widely used to describe pores/void space and using it discuss other mineral phases is confusing. Especially considering Nature Communications is a multidisciplinary journal I think most readers will also be confused. Perhaps instead call it Volume Fraction of Bulk?
- The error bars are "uncertainties in population density". Is this an uncertainty on the measurement itself or a stdev of the population? Also why are some of them so much larger in b)?
- Furthermore, what is the spatial resolution of these measurements? Can this explain the curves between 0 and 5 mm? This should be discussed somewhere in the Methods or the Supplementary materials.
- It would be nice to have an explanation of the R-value somewhere in the graph or in the caption.
- Fig d) Again this comes with some major assumptions that are not addressed. Furthermore, I assume there is some errors associated with these measurements that should be plotted!

Line 157-172: I found this section to be particularly confusing and did not understand until I looked at S4 what you did. I think it might help if you explain with examples. Again, if this was a longer format paper you could bring S4 to the main manuscript and explain this a bit better. In any case, you make some major assumptions that crystals growth rate is constant regardless of the phase and that orientation of the large crystals stays constant, that the there is no progressive growth of the large crystals, and that there is no further settling (which seems like a pretty big assumption considering the findings of the paper…). While I understand to simplify things you need to make some assumptions these are some fairly major ones which are not adequately touched. A more detailed description of the method and it's limitations is required.

Line 161: It is quite unclear from what you have written what the "composite SDP lines" are. It took me at least 3 reads to understand. I would recommend you either label them as such on the figure or cite the figure here.

Line 163-164: Cite the figure here

Line 169-170: You only have one granite sample so how can you trust this is the correct value? Is there any evidence of heterogeneity in the granite samples?

171-172: It is unclear to me if you are referring to 45-55 vol% for your samples or just in general… If your samples then please ref the figure/table that shows this.

Line 171: You only use the short form RPT three times in the paper, and one of them is when you introduce it. There are a lot of short forms already so I would suggest you leave this one long form.

Line 179-184: IT is unclear what you corrected… Is there something missing in the Supplementary material? There is no discussion of the correction in the Sup materials I have access to.

Figure 4: This figure I found very confusing.
- It is exceptionally unclear how do the lines in a turn into points in b?
- What are the 4 pointed stars in b?
- The legend in a) only makes sense if you have looked at the supplementary figure…perhaps make this a 2x2 figure and add the supp figure to this figure?
- I also initially printed the MS and colours of the points in the figure are indistinguishable in black and white.

Line 208 – 216: The lack of legend in Fig 5 makes it very difficult to understand where you got these values from.

Line 225: What is secondary RPT? How is there a second threshold? Perhaps this is my own ignorance but I really don't understand.

Line 239: The wording "the apparent lack of a parental magma" in my opinion makes it seems a bit like you think there is no parental magma at all as opposed to no parental magma exposed/erupted. Maybe re-word to "The lack of parental magma at surface for the …etc"?

Figure 5:
- It's unclear to me here if these are the diagrams from ref 16 that you have just put your data on, or if you used equations from ref 16. In the former – what is the basis for their modelling? It looks to me like they looked only at granites and not syenite…
- What is the difference between the r-value and Clustering Index (R)? If nothing then why is the Y axis of Fig 3 and 4 different than Fig 5? If different this needs to be explained. Same for the x-axis.
- What is the difference between the symbols? The scale on the figures really make it seem like a and c are the same figure.
- It took me several read to understand where these numbers were coming from. Perhaps reference Fig 4 in the caption to explain this?

Line 252-254: I think you need to be a bit careful here with what you mean by "evolved". Here you are using the term evolved to be enrichment in SiO_2. But you model clearly shows magmatic evolution that results in a decrease in SiO_2. This still evolved, just evolved in a way typical for alkalic systems.

Line 260: The term "another accessory" is vague… It really makes me wonder what this mysterious accessory is and why can it not be named?! I would suggest you just name it or change to "other accessory minerals" (if there are multiples).

Fig 263: I'm confused why there are the numbers in the sentence and also in brackets? What are these different numbers?

Line 288-289: I'm not sure papers written 15-20 years ago can be considered "Recent studies"…

Line 290-294: There are more updated studies which suggest transcrustal systems with variable melt fractions (E.g. Cashmann et al 2017; Paulatto et al 2022).

Line 312: What do you mean by a crystal rich magmatic density current?

Line 314: Change "submitted to" to "subjected to"

Line 313-316: I'm not sure I follow on what basis you think there is convective stirring?

Line 332: For clarity add the word uniformly at the end of the sentence "... they will all move downwards."

Line 343: Would love to see a derivation of this equation in the Supplementary material. I am confused at how if Vset = Vflu then Velu = Vset-Vflu =/= 0? Also where is f(c') coming from? Clearly, I am missing something key, but not sure what and I think a derivation of the equation would help.

Line 346: Did you consider any syn-eruptive growth???

Fig 7:
1) Would be great to have somewhere written on the figure what you took at the magma upward flux speed of the magma.

Do you results change significantly if this changes?
2) I think the scale on the x-axis is not very useful for typical crystal sizes. I understand why you would want to do the calculations in meters but crystals are usually on the mm scale so I recommend you change this
3) I found the dashed vertical lines to be confusing. The inflection point for the volcanic line makes sense because you used this size as key part of your calculations but the dashed line for the plutonic crystal makes me think that there should be a set of equations you ran for the plutonic crystal max. But this is not the case. I think the fact that they are presented as the same thing, but the equation uses one and not the other is confusing.
4) It might be helpful to denote the xtal movement direction visually in the plot instead of just writing it in the figure caption… this is a very fundamental thing and it is not apparent from the plot alone what is happening. Maybe just add up and down arrows on either side of the line? Or some text?
Line 367-379: I find this logic somewhat circular… You state that the size changes whether a particle will sink or float, but you have set what size a particle will sink or float at. You have good reason to choose this, but for this system and its specific viscosity. We know you model cannot be perfect because this critical size is the same regardless of an order of magnitude viscosity change. Yet your discussion/the plot is very centred around the size a crystal is dictates if it sinks or floats…. Which you decided…
Line 370-371: Please use significant digits.
Line 395: The word necessitating suggests to me that you MUST have a small plutonic counterpart for massive eruptions. Your results suggest to me that you COULD. If you think it is a requirement to have a small plutonic counterpart, I think you need some stronger evidence.
Line 413: Is the S you are taking from Figure 3? I assume yes but this should be clearly stated.
Line 417: changing the unit mid-sentence is confusing. Stick with ky or years.
Line 425-426: I'm not sure you can say it far exceeds. Fig 7 shows settling times of 10^2-10^5 years which overlaps with you extremely wide range of 0.2-10 ky.
Line 429 – 431: If this is true, I would expect to see some zoning??? It looks like in Fig 2 there is some with K. Maybe discuss this a bit more, or change the maps to show an element which shows this stronger???
Line 486: What is (s.l.) for?
Figure 8: Please write out "crystal cumulate", "trapped melt" and "extracted melt". You introduce a lot of short formed in this manuscript which I think makes it harder to read. Since you only use these shortforms once in this figure and you have space to write out the words, I suggest you avoid the shortforms here.
Fig 9:
- Spelling mistake on word "Compositional"
- For e) and f) you already have the words melt extracted and crystal cumulate so I don't think it is needed to have ME and CM and it makes it a bit busy. IF you want to make it clearer maybe make the text match the colours? Or label with a } symbol?
- What is the y axis for e/f??
Line 519: Manually tracing 300-1000 crystals per sample is A TON of work!! Is there a reason why you didn't try to segment with image J or another software? There are a lot of great and free options out there. This approach, while I'm sure has a great control on details, is also not very reproducible… Furthermore, is there a reason for only 300 for some samples? From what I understand in CSD there are larger quantities needed?

Version 1:

Reviewer comments:

Reviewer #2

(Remarks to the Author)
I have carefully reviewed the revised version of the manuscript. Overall, I believe the manuscript has improved substantially in clarity and structure following the authors' revisions. The argumentation is now easier to follow, and several additional explanations introduced in the revised text help the reader better understand the methodological framework and the interpretation of the results.

The introduction is well written and clearly frames the central problem addressed in the study: the difficulty of recognizing silicic cumulates based on textural evidence, particularly given the polymineralic nature of these rocks and the potential for re-equilibration textures. The authors successfully contextualize their work within the broader and still highly relevant question of volcanic–plutonic connections in felsic magmatic systems. The objective of the study (to evaluate crystal–melt segregation processes in the case study presented) is clearly stated and well justified.

In terms of impact, the most significant contribution of the manuscript lies on its discussion of crystal–melt segregation processes in silicic reservoirs. In particular, the conclusion that melt extraction may begin at lower crystallinities than predicted by classical mush models represents an important conceptual advance. The statement that "our findings suggest that melt extraction began at lower crystallinities than predicted by the classical mush model, resulting in higher melt extraction efficiency than initially computed" effectively captures the novelty and implications of the study.

The presentation of the analytical results and modeling has also improved in the revised version. I find that the additional explanatory sentences included by the authors, for instance regarding the interpretation of the R-value, are helpful for

guiding the reader through the methodology. There remain a few minor points where the presentation could be further clarified (see attached pdf) so that readers do not need to revisit the text multiple times to follow certain methodological aspects, but overall the manuscript reaches a high standard consistent with what is expected for a journal such as Nature Communications.

The section introducing the dynamic mush model is also clearer in the revised version. The authors now begin by briefly describing the classical mush model and then contrast it with their results, particularly in terms of the crystallinity range over which crystal–melt separation may occur. The additional definitions introduced in the revised manuscript are welcome and improve the clarity of the text (lines 336-343).

Overall, the manuscript now presents a coherent argument showing how the proposed dynamic mush model links textural observations with broader implications for the nature and dynamics of shallow magmatic reservoirs, their role in the generation of felsic magmas, and their capacity to feed caldera-forming eruptions. In my view, the authors have addressed the comments raised in the first round of review in a satisfactory manner.

Regarding the broader significance of the work, the study provides quantitative textural evidence for crystal–melt segregation processes operating in a shallow silicic magmatic reservoir. These results have implications for understanding the formation of felsic cumulates, the internal dynamics of magma reservoirs, and their relationship with large volcanic eruptions. The quantitative analysis of crystal size distributions and related textural parameters suggests that crystal–melt separation may operate over a wider crystallinity window than predicted by classical mush models and that dynamic processes, such as synneusis, may enhance segregation efficiency. These insights contribute to ongoing discussions about the origin of silicic magmas and the assembly of magma reservoirs capable of feeding caldera-forming eruptions.

The work is therefore likely to be of significant interest to researchers studying magmatic systems, volcanic–plutonic relationships, and magma reservoir dynamics. The integration of quantitative textural analysis with compositional modeling is particularly valuable, and the case study itself appears to be exceptionally well characterized.

The conclusions are generally supported by the data and analyses presented. I do have some reservations regarding the interpretation of certain textures as evidence of synneusis. While this interpretation is plausible and consistent with the proposed dynamic framework, such processes are inherently difficult to demonstrate unequivocally in the rock record. Similar textures could potentially arise through alternative mechanisms. For this reason, I suggest presenting this interpretation somewhat more cautiously. This point is addressed in more detail in my annotated comments in the attached PDF.

In general, I did not identify major flaws in the data analysis or interpretation. The manuscript appears complete, and the supplementary material is useful and appropriate. The remaining issues are relatively minor and mainly concern clarity of presentation and explanation in specific passages. Addressing these points would further improve the accessibility of the manuscript but does not require substantial revision.

The methodology is sound and meets the standards expected in this field. The quantitative approach to textural analysis is appropriate and robust. One point that could improve reproducibility concerns the raw measurements used for the CSD and SDP analyses. Unless I overlooked them, I did not find a table including the individual crystal measurements (e.g., crystal sizes, positions within the section, and classification into size bins or families). Providing these data as a supplementary table would facilitate independent validation of the calculations and further strengthen the methodological rigor of the study.

In summary, I believe the manuscript has been substantially improved and now presents a strong and well-supported contribution to the study of crystal–melt segregation in silicic magmatic systems. The remaining comments provided in the annotated PDF are intended mainly to improve clarity and precision in the presentation.

Reviewer #3

(Remarks to the Author)
Overall comments:
Thanks for your significant work to improve the explanation of the modelling and errors/limitations. I think the paper has been improved. I still have a few general comments, as well as some minor comments (below):
1) I still think the paper could be improved by stressing the interest of this paper to a broader audience. The changes you made helped but I think you can spell it out a bit more, especially considering this is a Nat Comm submission. Would your findings have any implication for other high-silica caldera forming eruptions world wide? It would great to see this applied to some of these systems (though that is a lot of work) or at least mention a few other systems that would be impacted by your findings. The fact that you can get this sort of volcanism with a lower crystallinity is very interesting, but you can still stress this better I think.
2) Thanks for the further explanations about CSD in the supplementary! I think that really strengthens your arguments. However, the issue of resolvable crystal size has still not been properly addressed (e.g. add something on Figure 3 to show there is a resolution limit). And I think it is also important to include images you did CSD on… you have a few images in the Supplementary but since this is your main dataset it is important to have this accessible and available.

Line by line comments:

Line 18: I think to make this a bit more clearer I'd replace "the former" with syenite.
Line 19: Do you results indicate ONLY early melt extraction? Or also a smaller %? The smaller percentage is the thing that I found very striking and more important!
Fig 2: I think labelling the minerals in this figure, like you have done in Fig1 would be helpful. With respect to my previous comment about the scale bar – I meant the colour. You have green = K… but is dark green 10, 2 or 0.2 % Please add a compositional scale.
Line 125: It is unclear to me which intercept? Again, I'm not a CSD expert so maybe I'm missing something obvious, but this is supposed to be a Nat Comm paper, so it needs to be approachable to a general audience. Also maybe ref to the figures in the sentence for clarity? (e.g. "Regular trachyte (Fig 3a red) and the medium grained granites (Fig 3b blue))
Line 125-126: Missing a reference (or ref your supplementary Fig 1 here).
Line 117-134: This section I still this is missing a discussion regarding resolution of your method. I think the kink/hump pattern you see is probably actually because of a resolution issue, which you also agree with (in part) in your rebuttal. Yes, there will be a decrease in xtal size in the late stage but you cannot know this unless you account for the resolution problem. It looks like from your supplementary data you only did the CSD with one resolution. There is a lower limit of what size crystals you can functionally measure with any magnification, which you have one sentence about in the supplementary materials. My recommendation is that you determine what the lower limit of measurement is and plot that on Fig 3 and add at least one sentence here or in the Figure caption about the resolution limit.
I don't know if there is CSD literature which has done this (maybe Gualda 2006?) but I can point you to a nice paper by Thomas Shea (Textural studies of vesicles in volcanic rocks: An integrated methodology, 2010) which discusses this same problem but for bubbles (section 3.4 Choice of Imaging magnification)
Fig 3: See comments for line 117-134.
Additionally, my comment from the previous rendition still stand ("I cannot tell which one is kinked – the lines are a bit too thick, so it is hard to see which is connected to which. Maybe consider making them thinner or changing the shade slightly (e.g. warm tones = syenites, cool = granite)? It might be helpful to indicatewhere you think the kink is? As it stands, I am not convinced that there is a kink.")
I would suggest you make the differences between the reds clearer… maybe I am a bit colourblind but the difference between the dark and light red is really hard for me to see. And it is impossible to tell how the same shades of red (fig 3b) follow through. I would suggest each line gets it's own colour, but keep the tones the same (e.g. med grained syenites are orange, med to very coarse syenites are a dark red, red, dark pink, light pink etc).
Line 186-201: With respect to your answer to "You only have one granite sample so how can you trust this is the correct value? Is there any evidence of heterogeneity in the granite samples?" in the rebuttal – this makes sense but I still think it is not properly stressed in the MS that these samples are very homogenous. I suggest you add a sentence somewhere in this paragraph that explains that! I cannot know the samples are homogenous as a non-expert of the CAC-VIPS without you telling me.
Line 203: I find the use of the word compaction throughout the MS a little bit confusing… you redefine it at line 338 but it might be more useful to define what you mean by compaction here instead. Compaction to me make me think that there is a force (other than gravity because that would be settling) driving the crystals to compact… but not sure what this force would be (at least at this stage in the MS).
Line 212: What is the natural correction? This is the first time in the manuscript you mention it. It would be helpful for a clearer explanation of what you are defining as the natural correction.
Fig 4: I still think it would be helpful to include the Sup Fig 4 figures in this figure to better understand what you did. It is good to have a visual representation too. Since you keep stressing it is a novel approach then it is worth it to properly explain it.
Line 270: Thanks for your explanation of the secondary ridged percolation threshold in the rebuttal!
Line 394: Thanks for the additional information in the Supplementary to derive this!
Line 420: Perhaps ref to Sup Figure 8 here as well?
Line 470-472: I still think you have significant overlap in the two timescales you present here. I don't think you can say it clearly exceeds when there is 8,000 years of overlap!! Perhaps you could say the maximal times exceed?
Supplementary methods: Thanks for the additional details here! I found this to be very helpful and I think rounds the paper out a bit more for a general audience.
Sup part 5: is dynamic viscosity including crystals???
Fig 8: I still don't understand how the different viscosities have the same d0? It is very non-intuitive to me… I wonder if you are accounting for the different densities you would get from a mafic v felsic phenocryst? Perhaps it would be helpful to know what you took at the density of the crystal and melt for Fig 8b? I'm missing something here!
Sup Fig 2-3 Is this all the data? I only see three samples here and you clearly have more. I cannot check you data (the CSD) if you don't include it…
Overall minor suggestion: I found it a bit confusing that there are supplementary Fig 1 and figure 1 in this document. Maybe just combine it all (or extend the numbering system?).

Open Access This Peer Review File is licensed under a Creative Commons Attribution 4.0 International License, which permits use, sharing, adaptation, distribution and reproduction in any medium or format, as long as you give appropriate credit to the original author(s) and the source, provide a link to the Creative Commons license, and indicate if changes were made.
In cases where reviewers are anonymous, credit should be given to 'Anonymous Referee' and the source.
The images or other third party material in this Peer Review File are included in the article's Creative Commons license, unless indicated otherwise in a credit line to the material. If material is not included in the article's Creative Commons license and your intended use is not permitted by statutory regulation or exceeds the permitted use, you will need to obtain permission directly from the copyright holder.
To view a copy of this license, visit https://creativecommons.org/licenses/by/4.0/

Response to Reviewer #2 Comments

Reviewer #2 (Remarks to the Author):

I am pleased to provide my assessment of this manuscript, drawing on my expertise in igneous petrology, plutonic–volcanic connections, and quantitative petrography. From the outset, I would like to emphasize that the manuscript is clearly written, well organized, and appropriately contextualized within the current literature. The authors make effective use of recent work, and the results are presented in a way that is accessible to a broad readership. I recommend that this manuscript be accepted for publication in Nature Communications after minor revisions addressing the points outlined below.

The study emphasizes the dynamic nature of magmatic systems, in contrast to previous static models. By applying quantitative petrography techniques, particularly the analysis of crystal size distributions (CSD), the authors provide new insights into the mush paradigm. The manuscript contributes to the ongoing debate regarding the volcanic–plutonic connection and the elusive nature of silicic cumulates, issues also highlighted in recent studies such as Clemens et al. (2022). The originality and potential impact of this work are evident, and I believe it represents a meaningful step forward in our understanding of crustal magmatic processes. In addition, the study provides valuable evidence supporting the connection between rhyolites and silicic intrusive rocks, an issue that is rarely documented in detail and remains the subject of ongoing debate.

The methods are robust, based on established approaches but applied here in a novel and systematic way that emphasizes the dynamic character of mush systems. The quality of the petrographic data and its presentation are adequate for supporting the conclusions. One limitation lies in the CSD analyses of volcanic rocks, where some crystals show evidence of dissolution or partial regrowth. While this complicates interpretation, the authors mitigate the issue by focusing primarily on plutonic samples. This limitation does not undermine the manuscript's overall conclusions.

In general, the conclusions are well supported by the data. The key inference that compaction occurs at lower crystallinities derives directly from the petrographic analyses. However, the claim that the system must have assembled rapidly (lines 396–397) is less

convincing. The connection between this conclusion and the presented data is not clearly articulated, and additional explanation or supporting evidence would strengthen the argument.

The most suggested improvements:

• Clarify the reasoning behind the inference of rapid system assembly.

Response: We have based our reasoning for a rapid assembly in several previous works and in the variation of textures registered in the plutonic counterpart of the CAC-VIPS. Firstly, the caldera has been developed in a post-collisional tectonic setting during metacratonization of the crustal section studied. So, it was emplaced at shallow crustal levels within a cold host-rock, demanding heat advection to keep it "alive" until collapsing as a caldera-volcano. Secondly, the elutriation of fine-grained crystals resulting in the increase sorting of Afs in syenites points to a system marked by a significant upwards-moving magma flux, which is only possible during underaccretion of magmas within a crystal mush. The timing between consecutive pulses must be small enough to prevent the full crystallization of the previous pulses and it is necessary to keep the base of the pluton warm and submitted to lower undercoolings. Such process can result in the coarsening of the subhedral to euhedral Afs crystals and keep it as a melt-rich up to melt-dominated system. In this new version of the manuscript, we tried to make this interpretation clearer.

• Provide brief additional explanation in the text regarding how some parameters are interpreted, to help readers follow the method more easily.

Response: In this new version of the manuscript, we have included the basis of quantitative textural analysis in the supplementary material

• Discuss briefly the implications of dissolution and partial growth in volcanic crystals for the robustness of the CSD results.

Response: As we only observed evidence for partial dissolution and regrowth in quartz crystals, such a discussion remains out of question and beyond the scope of the manuscript. However, in fact, if Afs crystals were submitted to such a process under

‘equilibrium’ conditions, CSDs of the volcanic rocks are expected to record a progressive decrease in both crystal sizes and the Y-intercept (population densities). Only in the case of crystals being completely consumed, will the SDPs decrease accompanied by a decrease in the Afs modal volume. As our discussions are based on the plutonic record, such question has a further implication for the reactivation of the crystal mush preceding the caldera-forming eruption, which will be the focus of a future study.

For further clarity, I have also attached a PDF of the manuscript with detailed comments and suggestions marked directly in the text.

In conclusion, this is a clear, original, and significant study that contributes to our understanding of magmatic mush systems and the origin of high-silica rhyolites. With minor revisions, the manuscript will fully meet the standards of Nature Communications and will be of broad interest to the community.

Sincerely.

Detailed comments by Reviewer #2

Line 20: Could you give crystallinity ranges?

Response: The crystallinity range has been included.

Lines 23-24: Why cumulates are cryptic? Explain briefly.

Response: In this sentence we state that silicic cumulates are a mixture of cumulus crystals and entrapped residual melts. Additionally, further in this section, such as in lines 34-35, we discuss that silicic cumulates are cryptic because they are usually polymineralic instead of the monomineralic examples of mafic systems. Thus, the presence of residual melts and its polymineralic nature are both explaining why silicic cumulates are difficult to recognize.

Line 43: Changes made.

Line 46: The term "dynamic, melt-rich system" could benefit from clarification. Do you mean a system characterized by physical mobility? Crystal-melt interactions, time dependent, or more generally one that does not evolve toward a static framework? Please elaborate to avoid ambiguity.

Response: We appreciate the suggestion, and we have clarified the use of the term.

Lines 50-51: Please, provide a brief description of the basis of this interpretation (whole-rock geochemistry, isotopes, other)?

Response: These silicic igneous rocks are both cogenetic and "*consanguineous*", as supported by recent studies based on whole-rock geochemistry, zircon U-Pb geochronology, Sm-Nd and Lu-Hf isotopes, and zircon trace elements. In this reviewed version of our manuscript, all the references for this interpretation have been included and this sentence was rewritten to make clear the basis for this interpretation of volcano-plutonic connection.

Lines 69-71: Could the authors clarify the distinction between the pink and green facies? Is this differentiation based solely on color, or are there mineralogical/petrological criteria as well? Explain if this has relevance for the overall interpretation. Later in the manuscript, syenites are distinguished at different structural levels of the system. Is the facies division related to this framework?

Response: The main petrographic facies are mainly characterized by their colors, but it most probably results from a differential alteration due to hydrothermal circulation. They do not correlate with the structural levels of occurrences and other significant aspects besides differential alteration. We believe this information has been made clearer now in the manuscript.

Figure 1b: Quartz crystals appear either partially dissolved or incompletely grown. Could you clarify which case applies here, and how this is taken into account in your model?

Response: We appreciate the observation made by reviewer #2, and indeed we do have textural evidence, revealed by cathodoluminescence images, suggesting that qtz-crystals were partially dissolved and later regrown, forming a high-CL rim. However, we do not have any further evidence for such a process in the Afs crystals from the pyroclastic sequences constituting the caldera-forming eruption(s). Although in the trachydacitic/trachytic lava flows we can see some evidence of resorption (**Figure 1e**), most of the interpretations made

using textural analysis were based on the plutonic record, lacking such evidence of disequilibrium. In this sense, we did not consider such process, for simplicity.

Figure 1l: I don't see the synneusis in the photo. Could you indicate it?

Response: We appreciate the suggestion and following it we have added an indication to the attaching plane of crystals submitted to synneusis.

Figure 1 caption: To which lithology does the photographs correspond? Please clarify this in the figure caption.

Response: We appreciate and following it we have added an indication to which facies the photographs correspond to.

Figure 2 caption: (b) is missing. In the text you describe different facies (as pink and green, or different levels), so, are these photos representative of all of them? Be specific.

Response: (b) has been included in the figure caption and the proper designation of facies.

Lines 107-108: I don't see this sample in the Figure.

Response: We have changed Figure 2 to make this clearer.

Line 117: It would improve accessibility if the authors added a brief, one- or two-line explanation of how to interpret R values (e.g., what low vs. high R values implies, such as $R \approx 1$, $R < 1$, or $R > 1$). This would help a broader readership to follow the results more easily.

Response: This discussion has been included in the main text and explored in more detail in the Methods section and supplementary material.

Figure 3: What is represented with red lines? Are these "regular trachytes?

Response: Figure 3 has been modified to be clearer for readers.

Figure 3 caption - Lines 131-135: This should be part of the main text.

Response: This discussion is now part of the main text and was included in the supplementary material for a better understanding.

Lines 139-140: This would be helpful in the main text.

Response: This discussion is now part of the main text and was included in the supplementary material for a better understanding.

Lines 158-161: Did you do this in four steps? I'm missing this part, so, clarify

Response: This textural treatment was made in several steps, depending on the number of crystal size populations in each sample. As seen in the CSDs (Fig.3a-b), each sample is composed of several size populations, and based on classical theory of magma crystallization, the larger crystals should have crystallized earlier also following a “stepped” path as the one applied in our analysis. The composite SDP lines were constructed by progressively including each crystal size population in the SPD analysis, starting from the larger ones. Each successive crystal size is then included in the textural analysis, resulting in a composite path that is roughly coincident with the textural maturation observed. The aim of this approach is to check the spatial distribution of crystals based on their sizes. To our knowledge, this is a new approach that has never been adopted before, and it is useful to investigating in detail how the final texture results from the mutual relationship of its multiple crystal size populations. For example, this analysis shows that our samples probably underwent crystal accumulation until they achieved the rheological lock-up and then proceeded with crystallization during a phase of crystal over-growth (equilibrium textures). These composite paths suggest that primocrysts and overgrowth rims and late formed crystals are in fact represented by different textural trends, and when compared with a manual correction (similarly made by Fiedrich et al., 2017 to exclude the late crystallized phases), our SDP lines show that these results can be compared with the average of R-values and crystallinity of minimum and maximum points from the ‘compaction’ trend. Although this treatment can be made only by the manual correction of the late grown phases, our approach seems to be a simple way to solve this correction, especially for cases in which there are no preserved compositional variations from crystal cores to rims. It only depends on a large sample submitted to a detailed textural analysis. Each step of textural development is described in Supplementary Material 3, and the textural treatment is illustrated in our supplementary figure S4. In this new version of the manuscript, including the Supplementary Table, we hope to have made it clearer.

Lines 169-172: This is a pretty nice result.

Response: We appreciate your comment.

Lines 188-189: What are exactly the gray dashed arrows? I guess it is the R-crystallinity path after the extraction scenario (ii). Please, specify. I don´t know if I'm missing something, but it would be good to include the predicted path for scenarios (i) and (iii). Just for reference and discussion. You mention it, but dont show the result assuming that.

Response: We have included a brief description of each trend illustrated in the inset of Figure 4. However, including these paths in the main Figure could cause some misunderstanding that we are trying to avoid.

Line 208: What do you mean with this term? Could you define it?

Response: The term "textural maturity" has been poorly employed in this sentence, because it might refer to cumulates that undergone textural equilibrium by minimizing interfacial energy, often indicated by grain boundary rounding and increased dihedral angles (c. 120º). This is not the case, and we have improved this sentence to make it clear that the granitic sample probably reached the rheological lock-up.

Line 215: a or c?

Response: In this section, we refer Figure 5 as a whole, discussing the values of MEP and initial crystallinity from both methos used for textural correction.

Lines 218-227: This paragraph reads more like part of the discussion rather than the results. It may be clearer to move it to the Discussion section.

Response: We appreciate the suggestion offered by reviewer #3. However, we opted to keep the main discussion of our manuscript in the section where we introduce the "dynamic mush model". To do so, we have changed the name of this section, to avoid this misunderstanding. As the format of papers in NatComm are different from the traditional Introduction-Methods-Results-Discussion-Conclusion structure, we deliberately chose to keep the more broadly significant discussion of our work in the "discussion" section. However, there are multiple

minor discussions that are needed throughout the main text to explain the significance of our results and bridge between different parts of our work.

Figure 5: It would be helpful if you could briefly explain how the colored contours were calculated.

Response: We have added a new section in the Supplementary Material to explain the modeling of Špillar & Dolejš (2015). There, the reviewers and readers can assess a simplified explanation of the 3D and 2D modeling resulting in the diagrams to estimate mechanical modifications of igneous textures. We hope such a detailed explanation of their work will help in clarifying how we estimate melt extraction in our samples.

Figure 5 caption: Please indicate what the blue and red symbols represent, either in the figure caption or in the text.

Response: This information has been included in the figure caption.

247-250: Are you talking only about the volcanics? If that is the case, be specific.

Response: We were referring to both volcanic and plutonic occurrences, as is stated more clearly in the new version of our manuscript.

Line 278: with respect to other rocks? Or diverse?

Response: We have rephrased the sentence to make it clearer.

Lines 285-286: Honestly, I understand that this is according several models about the construction of magma chambers and the accumulation of "large" volumes of crystal-poor magmas, which it's interpreted to occur under high-injection rates (https://doi.org/10.1130/G34241.1). But I don´t see the reason to define compaction indicating a magma chamber. This should depend only on the crystal content of the magma, not necesarily indicating a magma chamber assembled at high injection rates. If you bulit a crystal-poor magma body by one pulse, independent on the size, do you think that "compaction" does not occur? Please reconsider this definition.

Response: We appreciate this comment and we fully agree with it, this sentence has been improved in the revised form of our manuscript reflecting the suggestion.

Lines 294-296: This is a great result, but, more specifically, you spread the crystallinity window of extraction, or at least you displace it. That is the main result.

Response: We appreciate this comment and we fully agree with it, this sentence has been improved in the revised form of our manuscript.

Lines 313-315: I'm sorry but I don´t see how your results support this. Do you see some particular textures? Other evidences to infer underaccretion and high-rates?

Response: In our manuscript, we interpret that the textural record can be used to interpret the mechanisms of magma differentiation and reservoir growth. We interpret that underaccretion is necessary to increase crystal sorting by promoting the elutriation of the smaller crystal sizes while keeping the lower part of the reservoir warm and at lower undercoolings, resulting in the textural coarsening of Afs. Furthermore, the formation of large crystal clots of coarse-grained Afs stuck together by *synneusis* require magma motion with freely suspended crystals. All this evidence, when considered together, support our interpretations. We hope this point has been clarified in the revised manuscript.

Lines 333-335: Could you explain briefly this interaction? What kind of interaction is? Is this mechanical? Chemical? Both?

Response: We mentioned the mechanical interaction between crystals, now properly stated in this new version of our manuscript.

Figure 7: Please, indicate in the figure the legend for dashed and continuous lines.

Response: Changes made.

Figure 7 caption: Why 500 m? Justify.

Response: We used the same distance used by Bachmann and Bergantz (2004), so that the results would be comparable. This is now mentioned in the caption.

Lines 378-379: Nice result.

Response: We appreciate the comment!

Lines 396-397: I insist, I don't see how your results support this interpretation.

Response: Please, see response to comments on lines 313-315.

Line 400: In which rocks?

Response: We were referring to the syenites, now properly mentioned in the text.

Line 404: You must describe this in the results or background.

Response: We now mention this information in the background section in this new version of the manuscript.

Lines 405-406: But this could be explained also by considering a linear geothermal gradient as initial condition of the crust. See: https://doi.org/10.1038/ngeo2959 and https://doi.org/10.1130/G34241.1

Response: This is an interesting observation. However, there is no considerable difference between the depths of the outcropping upper and lower regions of the Corupá Pluton, which is a relatively small intrusion with a maximum depth of 1.5 km emplaced at shallow crustal levels. Thus, this textural variation must result from heat advection rather than an increase in temperature controlled by the geothermal gradient.

Lines 413-417: This sentence could be rephrased to improve its fluency and readability. Why did you choose those growth values? Justify

Response: We have changed the sentence to make it clearer and properly cited the reference used to justify the values used for computing times of crystal growth.

Lines 420-421: 2–8 years, 200–800 years, and up to 20–80 kyr.

Response: Changes in the presentation of timescales were made to make it clearer.

Lines 429-430: I don't see this from your results. If there is a relationship between the CSD parameters and the structural location in the system, you should mention and show it.

Response: We appreciate this comment, so this sentence has been improved in the revised form of our manuscript.

Response to Reviewer #3 Comments

Reviewer #3 (Remarks to the Author):

This manuscript titled "'Mush in Motion: How dynamic crystal-melt segregation links silicic cumulates with caldera-forming eruptions" by Lino et al., aims to re-evaluate the mush model and introduce a dynamic mush model. The case study is Campo Alegre- Corupá system (Brazil). The study combines textural analysis with thermodynamic modelling. The main finding of the paper is that volumes of high SiO2 magma can be generated with low crystallinities via a dynamic reservoir where crystals are elutriated, creating crystal clusters which rapidly sink.

While this is a significant piece of work and I do think the findings and implications are interesting, I have come concerns about the many assumptions used that are not properly addressed. My major concerns are as follows (see line by line comments for further details):

- Utilizing 2D textural data for CSD, while a common technique, is one that comes with the major assumption that the 2D sections are an accurate representation of the 3D crystals. This is not addressed in the MS at all and there is no error presented with the measurements of the crystal axes.

Response: In this new version of the manuscript, we have stressed that this is a limitation of the CSD technique, thus the stereological corrections must be taken with caution. However, most of our discussion is based on the 2D distribution of crystal's centroids, as supported by the 3D models of Špillar and Dolejš (2015), and these quantitative analyses do not depend on accurate stereological corrections. In order to clarify this point, we have included an entire section of Supplementary Material to make it clear, by detailing the numerical models presented by these authors. Finally, by propagating errors based on the 5 best-fitted results of 3D crystal shapes, we have included the uncertainties in the measurements of crystal habits.

- The SDP calculations make several assumptions that are not addressed (e.g. variable crystal growth rate, continued crystal growth of large crystals during growth of small crystals, etc.) and the manual correction of these measurements is inadequately explained.

Response: This is an important point, as we respectfully disagree with the reviewer in a key point: Following CSD theory, SPD calculations themselves are not based on these assumptions because they are the simple estimation of the spatial relationship between crystals ***already formed***. In our study, additional SPD calculations were made to correct for equilibrium textures, irrespective if crystal growth has changed, or if larger crystals continued to crystallize during the formation of the smaller ones. Thus, the assumptions cited by the reviewer are not an issue in our quantification, as they are all variables acting during magma crystallization, which are accounted for in CSD theory. That said, we agree that the measurements made before correcting the final textures include assumptions that were not well explained. Our correction of textures was based on the textural development of igneous rocks, which include the late formation of overgrown rims and late interstitial crystals crystallized before the magma reached the rheological lock-up. We have clarified these issues in the new version of our manuscript.

- The modelling crystal settling speeds/timescales, while really cool and I think something that will be useful to many readers, only works with a major assumption of the critical crystal size. While this choice is rooted in natural samples, again, there are many assumptions that come with this decision (e.g. no crystal growth after separation from the original melt, it is actually the largest crystal found, etc). This model cannot be correct as at different viscosities we would expect the critical size to change. While of course models require some degree of simplification, the assumptions must be clearly stated. I suggest an additional supplementary figure that compares the findings for a variable critical size should be added to show how much their results deviated when changing this critical parameter. Furthermore, it was very unclear to me how this equation was derived, and I think this is important to have in the methods or supplementary materials.

Response: Once more, this is an important issue that should be clarified, and we thank the reviewer for pointing this out. While it is true that changes in viscosities result in different critical crystal sizes, this is accounted for in our model using Stokes' law. For this, we shift from the traditional perspective of a particle descending through a static fluid, to the

opposite referential, in which the fluid moves relative to the solid particles. However, and importantly, this relationship is still governed by the same equation. Thus, as correctly pointed out by the reviewer, by changing the velocity of an upwards-moving magma flux the critical particle size will also be changed. This is now clarified in the main manuscript. Also, following the suggestion of the reviewer, the new version of our Supplementary Material includes a new figure that illustrates how changing the velocities to values compatible with other critical $\boldsymbol{d_0}$ (*e.g.*, 5, 10, and 15 mm) has a major influence on the smaller crystals than the larger ones. The same treatment has been made for varying magma composition to account for contrasting viscosities. As expected, based on Stokes' law, an increase or decrease in magma viscosity is accompanied by a proportional variation in the velocities of suspended particles, which means that our approach can be easily extended to different conditions than those discussed in our study, which focused on silicic magmas. Additionally, as pointed out by the reviewer, it is true that this model does not consider crystal growth, as the equation derived in our study does not have a time-integrated form yet. This is now more explicitly stated in the manuscript. Finally, a *step-by-step* derivation of the equation describing elutriation velocities was also included in the supplementary material.

- Finally, the way the paper is written I don't think balances stressing the interest to a broad scientific audience and explaining what was done very well. The introduction does not make it clear the larger implications of this research question; this is truly not revealed until the end of the discussion. Furthermore, it took several reads of the methods/supplementary materials for the main manuscript to make sense, *so I think the MS could benefit from some significant re-writes for clarity or a change to a longer format article*.

Response: We appreciate the comments and we have taken this opportunity to rewrite significant parts of the manuscript. In particular, we now emphasize, in the introduction, that our model has the potential to change a long-stating paradigm associating crystal mushes with large silicic eruptions, by offering alternative scenarios for the formation of multiple and diverse silicic occurrences worldwide. Furthermore, it increases our potential to investigate how magma reservoirs can feed silicic caldera-forming eruptions.

Line by line Comments:

Line 19: The opening line cites what you call a "central topic in petrology" yet you only cite papers first authored by Olivier Bachmann.

Response: Thank you for pointing this out, as this indeed causes an impression which we did not intend. This first paragraph has at least 10 references supporting the statement that the volcano-plutonic connection is still a central topic in igneous petrology. However, most references have been cited in the following sentences, in which specific processes are addressed. In order to correct this, we now cite the first 15 references in this first sentence and then cite them again throughout the paragraph aiming not to only cite papers first authored by Olivier Bachmann, one of the coauthors, in the first sentence.

Line 29: I found this sentence difficult to read… perhaps because of the tense of this sentence? Maybe change to "have often experienced" or "often experience".

Response: This sentence has been rewritten with the aim of making it clearer.

Line 56-62: This section I got a bit confused with when came when relative to each other. The word "early" is used a couple times, but it is unclear these are both the same age and not? A stratigraphic column on the side of Fig 1 might help. Alternatively, if available some ages for the different units may help? If the units have names, then I think this might also help to clarify what you are talking about and when. I understand you have tried to simplify it to make it easier for the reader, but since time is a very important part of your discussion, I think it is valuable to include it.

Response: There are different stages for the silicic occurrences in the CAC-VIPS, as supported by their stratigraphical relationship. We identified effusive trachytic/trachydacitic occurrences before and after the caldera-forming eruption(s), and in the main pyroclastic sequences associated with the caldera collapse we also observe a temporal variation in the volcanic products. There is a Plinian-type phase marked by high-silica ignimbrites, followed by the main sequence of welded-tuffs erupted during the caldera-collapse. Such

stratigraphical variation and its association with the volcano-plutonic connection have been discussed elsewhere (*e.g.*, Lino *et al*., 2023 ChemGeo; Quiroz-Valle *et al*., 2023). However, this was difficult to follow in the former version of our manuscript, as identified by the reviewer, and so we have improved these sentences to make it clearer.

Line 67-71: The different facies (green and pink) are introduced here while explaining the mineralogy which I found abrupt. Maybe add an intro sentence/statement that explains the syenites have different facies? Or average the two facies and present that?
Response: This paragraph has been rewritten and the association by colors of the different syenitic facies was made clearer.

Fig 2: Since this figure is used for the CSD it would be useful to have one or two of the maps showing how where what you chose as the measurements for a few crystals. Especially since the chemistry of these maps is not discussed in detail so I assume you included this figure as it is what you used for the CSD. Additionally, could you add a scale bar to make these maps quantitative?
Response: The compositional maps and the petrographic images were used in combination to exclude the overgrown rims (usually K-rich) and the late interstitial crystals formed before the mush reached the rheological lock-up. In this new version of the manuscript, we have added a drawn of the outline of crystal-clots exhibiting a K-rich rim. Unfortunately, the internal division of crystals has can often only be recognized in the petrographic images, which prevents us from illustrating this further in the compositional maps. Furthermore, all maps share the same scale bar, as illustrated in the late, Fe compositional map.

Line 104: A basaltic flow? Fall?
Response: This sample is a basalt from an extensive lava-flow, now clearly stated in this new version of our manuscript.

Line 107-108: I cannot tell which one is kinked – the lines are a bit too thick, so it is hard to see which is connected to which. Maybe consider making them thinner or changing the shade slightly (e.g. warm tones = syenites, cool = granite)? It might be helpful to indicate where you think the kink is? As it stands, I am not convinced that there is a kink.
Response: This figure has been changed to make this information clearer.

Line 111: Do you mean Ostwald ripening?
Response: We were referring to coarsening in the sense of Ostwald ripening, as now properly stated in this new version of our manuscript.

Line 112: What do you mean by “hump shaped distributions?”
Response: CSD lines are usually straight segments with a progressive decrease in the number of crystals at smaller sizes a pattern that is commonly described as hump-shaped in the CSD literature (*e.g.*, Higgins, 2006). This downward displacement typically reflects a natural decrease in smaller crystal sizes, and it can be associated with crystal accumulation, as discussed in the cited references. In this new version of the manuscript, we believe the terminology regarding hump-shaped distributions is clearer for those unfamiliar with the CSD technique.

Line 117: The right side of this equation seems wrong. There is a close bracket that doesn’t have an open bracket. Maybe one of the “/” should be “(“ ?
Response: This equation has been rewritten in its correct form.

Line 119: How are this r distance chosen? Are you doing this? Is there a model that you are using that does this? It is unclear from what you have written (including the methods and supplementary) how this is chosen.
Response: In this new version of the manuscript, both supplementary data and the methos section have been significantly expanded, detailing, among other things, how R-values are computed for the quantitative measurement of spatial distribution patterns.

Line 124-128: CSD calculations comes with major assumptions that you can extrapolate 3D data from 2D data. This is not addressed at all here and should be.

Response: The reviewer is correct that the correspondence between 3D and 2D forms is certainly a drawback of quantitative textural analysis. However, this assumption is the basis of CSD technique, which has been used for over 30 years. Major problems in the stereological corrections of crystal's 3D shapes can result from both multiples stages of crystallization and multiple crystal sizes, and in the large relative variation of their axes. Most of our samples are clearly constituted of simple equant to tabular/prismatic crystal shapes gathered in a single crystallization stage, which makes the classical assumptions of CSD a minor problem. Furthermore, our most relevant discussion is based on SDP rather than CSDs, which are not affected by stereological corrections. We have reviewed our manuscript including statements that summarize all points above to clarify to the reader the potential limitations of our approach.

Figure 3:

- I found the use of the term Porosity in this figure and the rest of the manuscript to be extremely confusing. I understand that Jerram et al 1996 use this terminology but porosity widely used to describe pores/void space and using it discuss other mineral phases is confusing. Especially considering Nature Communications is a multidisciplinary journal I think most readers will also be confused. Perhaps instead call it Volume Fraction of Bulk?

Response: We agree with the observation offered by the reviewer. We have changed this term for "non-crystal volume (%)" referring to the complementary volume of phases not considered in the quantitative textural analysis.

- The error bars are "uncertainties in population density". Is this an uncertainty on the measurement itself or a stdev of the population? Also why are some of them so much larger in b)?

Response: These uncertainties are computed with the software CSDCorrections (Higgins, 2000) by propagating errors, and they typically represent problems in both small and larger

crystal sides of a size distribution. For larger crystal sizes, uncertainties are relatively greater because of the small number of measured crystals. On the other hand, finer crystal populations are usually smaller than the predicted number of crystals by the CSD theory because of the spatial resolution of measurements and the diminishing in their frequency owing to the dynamics of crystallization of final liquids. By detailing how the uncertainties were quantified in the "Conceptualization and Methodology" section of the manuscript, we hope that the reader will be able to assess the basis of quantitative textural analysis and understand the application and limits of the methods used. That said, we also stress that it is beyond the scope of our study to explain the basis of quantitative textural analysis, which has been properly made in the list of references included.

- Furthermore, what is the spatial resolution of these measurements? Can this explain the curves between 0 and 5 mm? This should be discussed somewhere in the Methods or the Supplementary materials.

Response: The curves in the CSDs (commonly described as hump-shaped, see comment above) are typical of these types of measurements, and that is why the logo of the *CSDCorrections* software has the same shape (Higgins, 2002). It results from two main factors, including the spatial resolution of measurements and the natural decrease in the number of small crystals formed during the late stages of magma crystallization, causing a diminishment in the final nucleation rates that is smaller than predicted by theory. In this new version of our study, we have included a full section in the Supplementary Material to further explain the basis of theoretical and applications of CSDs.

- It would be nice to have an explanation of the R-value somewhere in the graph or in the caption.

Response: We have detailed how the measurement of crystal ordering works and how the R-values are compared to each other in a full section included in the new version of our Supplementary Material.

- Fig d) Again this comes with some major assumptions that are not addressed. Furthermore, I assume there is some errors associated with these measurements that should be plotted!

Response: The propagated errors have been computed and are now included in the new version of this figure. Furthermore, it noteworthy mention that the caveats about 3D/2D crystal shapes derived from stereological corrections, and mentioned in the previous reply, also address this point.

Line 157-172: I found this section to be particularly confusing and did not understand until I looked at S4 what you did. I think it might help if you explain with examples. Again, if this was a longer format paper you could bring S4 to the main manuscript and explain this a bit better. In any case, you make some major assumptions that crystals growth rate is constant regardless of the phase and that orientation of the large crystals stays constant, that the there is no progressive growth of the large crystals, and that there is no further settling (which seems like a pretty big assumption considering the findings of the paper…). While I understand to simplify things you need to make some assumptions these are some fairly major ones which are not adequately touched. A more detailed description of the method and it's limitations is required.

Response: This is certainly one of the most significant parts of the manuscript and it investigates how the final textures of holocrystalline rocks might reflect the processes resulting in it. To our knowledge, this is a new approach that has never been done before, and is very useful for investigating in detail how the final texture results from the mutual relationship of its multiple crystal size populations. We highlight the novelty of the approach in the revised manuscript and dedicate an entire section of the Supplementary Material to explain our approach in detail. That said, we respectfully disagree with parts of the reviewer's comment. First, no assumptions about crystal growth rates and orientation are necessary for this analysis which merely characterizes how the largest, and usually subhedral to euhedral crystals, are arranged relatively to each other. Because it proceeds stepwise from larger to smaller crystals, it testes how the rock texture might be "constructed" during progressive crystallization. It is well known that final textures are composed of early-grown

primocrysts, and overgrowths and late grown small crystals formed after magma reaches its rheological "lock-up". Such progressive formation of crystals, owing to the continued crystallization of magmas, may or may not record mechanical modifications, and correctly identifying them is fundamental to our approach. Starting from the larger crystal populations, we can observe a progressive increase in crystal clustering associated with modal abundances that follows a trend of crystal accumulation until ~55vol.%. From there on, it increases slightly or it is kept almost constant until crystallization finishes, suggesting a stage of '*textural maturation*' probably owing to crystal overgrowth. If there was no mechanical modification (*i.e.*, clustering), textural trends would follow the Random Texture Trend (RTT) from ref.16, in which the gentle increase in R-values are a result of crystallization. As supported by our Supplementary Material 4, if there was mechanical modification, it should be recorded somewhere in between the trend of '*compaction*', registered by the relative position of centers of 2D crystal sections. Thus, we show that we can effectively separate primocrysts from late-grown rims and small crystals either by using detailed analysis of compositional maps and petrographic evidence, or alternatively simply using quantitative textural analysis. Our modeling shows that both approaches converge with the same results. And even more significantly for the rest of our manuscript, it means that our volcano-plutonic system records melt-extraction and cumulate formation at stages of crystallization never seen in the literature.

Line 161: It is quite unclear from what you have written what the "composite SDP lines" are. It took me at least 3 reads to understand. I would recommend you either label them as such on the figure or cite the figure here.

Response: The composite SDP lines were constructed by progressively including each crystal size population in the SPD analysis, starting from the larger ones, with the aim to check the spatial distribution of crystals based on their sizes. This is a new approach, to our knowledge never done before, that investigates in detail how the final texture results from the mutual relationship of its multiple crystal size populations. As seen in the CSDs (**Fig.3a-b**), each sample is composed of several size populations, and based on classical theory of

magma crystallization, the larger crystals may have crystallized earlier also following a "steeped" path as the one applied in our analysis. Each successive crystal size is then included in the textural analysis, resulting in a composite path that is roughly coincident with the textural maturation observed. Our samples probably underwent crystal accumulation until they achieved the rheological lock-up and then proceeded with crystallization during a phase of crystal over-growth (equilibrium textures). Each step of textural development is described in Supplementary Material 3, and the textural treatment is illustrated in our supplementary figure S4. These composite paths suggest that primocrysts and overgrowth rims and late formed crystals are in fact represented by different textural trends, and when compared with a manual correction (similarly made by Fiedrich *et al*., 2017 to exclude the late crystallized phases), our SDP lines show that these results can be compared with the average of R-values and crystallinity of minimum and maximum points from the '*compaction*' trend. Although this treatment can be made only by the manual correction of the late grown phases, our approach seems to be a simple way to solve this correction, especially for cases in which there are no preserved compositional variations from crystal cores to rims. It only depends on a large sample submitted to a detailed textural analysis.

Line 163-164: Cite the figure here

Response: The figure has been properly cited in this section.

Line 169-170: You only have one granite sample so how can you trust this is the correct value? Is there any evidence of heterogeneity in the granite samples?

Response: We only selected one granite sample because we chose, in this study, to investigate the compositional and especially the textural evolution of residual silicic cumulates (*i.e.*, in the case of the CAC-VIPS, syenites from the Corupá Pluton), which has gained little attention in the literature, and was rarely documented in association with the extracted melts that resulted in caldera-forming eruptions. In contrast, there is ample geochemical, stratigraphic, and textural evidence, summarized in the extensive research on the CAC-VIPS we have conducted in recent years (Quiroz-Valle *et al*., 2019,2023; Lino *et al*.,

2021, 2023a, 2023b, 2024), that the granitic cupola represents the residual, high-silica melt extracted from a felspathic mush. In this sense, given the compositional variability of the syenites compared to the granitic cupola, and our interest in the textural evolution of the cumulates, we chose to only include one sample of the high-silica plutonic rocks as a control sample to check if the same textural analysis works for samples without textural mechanical modification (which it does, see **Fig.4**). Thus, in our view, it is not necessary, and is not worth the additional work, to include several quantitative textural analyses of a relatively homogeneous granitic facies of the Corupá Pluton.

171-172: It is unclear to me if you are referring to 45-55 vol% for your samples or just in general… If your samples then please ref the figure/table that shows this.
Response: We have improved this sentence to make the statement clearer.

Line 171: You only use the short form RPT three times in the paper, and one of them is when you introduce it. There are a lot of short forms already so I would suggest you leave this one long form.
Response: Following this suggestion, we have changed the abbreviation to its full format throughout the new version of our manuscript.

Line 179-184: IT is unclear what you corrected… Is there something missing in the Supplementary material? There is no discussion of the correction in the Sup materials I have access to.
Response: The final textures of holocrystalline igneous rocks are constituted of primocrysts, which represents the first mineral phases that formed from magma before it reached the rheological lock-up, and the rims and late-grown minerals which finishes the process of crystallization. We have improved this new version of the manuscript to clarify this point.

Figure 4: This figure I found very confusing.

- It is exceptionally unclear how do the lines in a turn into points in b?

Response: The SPD lines correspond to a full analysis of spatial distribution patterns of crystals considering their size distribution. Thus, starting the analysis from the larger ones, each size population is then included incrementally until completing the whole volume of Afs crystals. The points represent the textures corrected for rimmed overgrowths and interstitial late crystals. In this new version of our manuscript, we hope this information has been made clearer for the reviewer and the readers.

- What are the 4 pointed stars in b?

Response: We have changed the figure caption to clarify what each motif represents.

- The legend in a) only makes sense if you have looked at the supplementary figure…perhaps make this a 2x2 figure and add the supp figure to this figure?

Response: In our opinion, the step-by-step explanation of how this figure was constructed is better suited as a Supplementary Material. In the revised version of the manuscript, we explain the approach behind this analysis in more detail, and the supplementary figure 4 is mentioned more often to highlight its importance.

Line 208 – 216: The lack of legend in Fig 5 makes it very difficult to understand where you got these values from.

Response: Figure 5 has been modified to group more clearly the results obtained through the different methods to correct the final textures, and its caption was also improved.

Line 225: What is secondary RPT? How is there a second threshold? Perhaps this is my own ignorance but I really don't understand.

Response: In classical magma rheology, there are two rigid percolation thresholds, corresponding to major variations in magma viscosity. These occur when: 1) a magma-suspension/crystal-bearing magmas becomes a mush (partially rigid liquid); and 2) when a rigid material nears mechanical immobility. The variations in viscosity, which correspond to

a progressive increase in the crystal fraction of the magma, lead to at least three different flow regimes, separated by the primary and secondary rigid percolation thresholds, as seen in the following Figure from Frontoni *et al*. (2022). We have improved the supplementary material and the main text to make this information clearer to the readers.

[*REDACTED*]

Line 239: The wording “the apparent lack of a parental magma” in my opinion makes it seems a bit like you think there is no parental magma at all as opposed to no parental magma exposed/erupted. Maybe re-word to “The lack of parental magma at surface for the …etc”?
Response: This sentence has been changed to make clear the selection of a primary magma composition for the thermodynamic modeling.

Figure 5: - It’s unclear to me here if these are the diagrams from ref 16 that you have just put your data on, or if you used equations from ref 16. In the former – what is the basis for their modelling? It looks to me like they looked only at granites and not syenite…
Response: This is a fair point. In this figure, we indeed plot our data over existing diagrams. These diagrams were constructed based on 3D modeling, and they can be used for any type of igneous rocks, especially monomineralic ones. In this new version of our manuscript, we

have included an entire section of Supplementary Material to explain how the diagrams of ref 16 (Špillar and Dolejš, 2015) work, and why we need to apply a textural correction before using it.

- What is the difference between the r-value and Clustering Index (R)? If nothing then why is the Y axis of Fig 3 and 4 different than Fig 5? If different this needs to be explained. Same for the x-axis.

Response: There is no difference between both, and we have changed figures 3 and 4 to make use of the same terminology.

- What is the difference between the symbols? The scale on the figures really make it seem like a and c are the same figure.

Response: The different symbols were used to distinguish between samples from which textures were corrected manually using petrography and XRF maps (eight-pointed star motif) form those corrected by our simplified textural analysis method (four-pointed star motif). The similarities between the results thus confirm that our simplified approach has similar outcomes. We have made changes in the Figure and its caption to clarify this.

- It took me several read to understand where these numbers were coming from. Perhaps reference Fig 4 in the caption to explain this?

Response: Thank you for the suggestion, we have referred to Fig.4 in the caption.

Line 252-254: I think you need to be a bit careful here with what you mean by "evolved". Here you are using the term evolved to be enrichment in SiO_2. But you model clearly shows magmatic evolution that results in a decrease in SiO_2. This still evolved, just evolved in a way typical for alkalic systems.

Response: As we are working on a silica saturated system, we have changed this sentence to make clear what we meant.

Line 260: The term "another accessory" is vague… It really makes me wonder what this mysterious accessory is and why can it not be named?! I would suggest you just name it or change to "other accessory minerals" (if there are multiples).

Response: Thank you for the suggestion, we have changed the term following it.

Fig 263: I'm confused why there are the numbers in the sentence and also in brackets? What are these different numbers?

Response: We appreciate the observation, and we have rewritten this sentence to avoid a misunderstanding of the values presented.

Line 288-289: I'm not sure papers written 15-20 years ago can be considered "Recent studies"…

Response: We agree with the comment, and we have referred to it as "classical studies".

Line 290-294: There are more updated studies which suggest transcrustal systems with variable melt fractions (E.g. Cashmann et al 2017; Paulatto et al 2022).

Response: We appreciate the references suggested, we have added in the revised text.

Line 312: What do you mean by a crystal rich magmatic density current?

Response: Following Marsh (1988b), based on previous works of Irvine (1987), we refer to crystal-laden density currents. In this new version of the manuscript, we hope to have clarified this terminology and its implications for our work.

Line 314: Change "submitted to" to "subjected to"

Response: We appreciate the suggestion.

Line 313-316: I'm not sure I follow on what basis you think there is convective stirring?

Response: The presence of numerous crystal agglomerates in the plutonic counterpart of the CAC-VIPS, including the pairs formed via *synneusis*, point out to a magma system

constantly submitted to stirring/motion. Crystal *synneusis* most commonly forms when freely suspended, faceted crystals gently collide and stick together in a mobile, crystal-poor magma, with continued growth locking them in place. Furthermore, *synneusis* pairs of large, euhedral/subhedral *Afs* crystals supports the interpretation that a dynamic, crystal-poor stage of the magma system has lasted for long periods, allowing crystals to growth. In this new version of the manuscript, we have improved this section to make these points clearer.

Line 332: For clarity add the word uniformly at the end of the sentence "… they will all move downwards."
Response: We appreciate the suggestion.

Line 343: Would love to see a derivation of this equation in the Supplementary material. I am confused at how if Vset = Vflu then Velu = Vset-Vflu =/= 0? Also where is f(c') coming from? Clearly, I am missing something key, but not sure what and I think a derivation of the equation would help.
Response: We appreciate this comment and, in this new version of the manuscript, we have provided a more detailed derivation of the equation used to compute elutriation velocities, as well as other situations for varying the critical crystal size and magma viscosities.

Line 346: Did you consider any syn-eruptive growth???
Response: This is a good point. There is no textural or other evidence for syn-eruptive growth of the macrocrysts, and most of the results discussed in our manuscript are based on the relative position of their centers, which should have been established during eruption. Thus, syn-eruptive growth is expected to exert a minor influence on such variables, except for the modal abundance of Afs, which could change, also to a lesser extent. Only in the lava flows we can observe micro crystals forming the matrix, but there is no further evidence for syn-eruptive growth of macrocrysts. In this sense, we assume that if this process occurred to some degree, it did not affect the outcomes discussed in our manuscript, which is why it has not been considered in our computing.

Figure 7: 1) Would be great to have somewhere written on the figure what you took at the magma upward flux speed of the magma. Do you results change significantly if this changes?

Response: Based on this comment, we have included a new figure to our supplementary material (**Figure 8**), showing that, for critical particle sizes of 10 mm, the upwards-moving magma flux needs to be ≥ 2×10^{-8} m/s to keep them in suspension or moving up. Changing the velocity of the upwards moving flux, naturally changes the size of the critical particle. However, as seen in Figure 8 of Supplementary Material, it has a more significant influence on the smaller particles.

2) I think the scale on the x-axis is not very useful for typical crystal sizes. I understand why you would want to do the calculations in meters but crystals are usually on the mm scale so I recommend you change this;

Response: We appreciate the comment, and we have changed Figure 7.

3) I found the dashed vertical lines to be confusing. The inflection point for the volcanic line makes sense because you used this size as key part of your calculations but the dashed line for the plutonic crystal makes me think that there should be a set of equations you ran for the plutonic crystal max. But this is not the case. I think the fact that they are presented as the same thing, but the equation uses one and not the other is confusing.

Response: Following this suggestion, please see the new version of **Figure 7**.

4) It might be helpful to denote the xtal movement direction visually in the plot instead of just writing it in the figure caption… this is a very fundamental thing and it is not apparent from the plot alone what is happening. Maybe just add up and down arrows on either side of the line? Or some text?

Response: Following this suggestion, please see the new version of **Figure 7**.

Line 367-379: I find this logic somewhat circular… You state that the size changes whether a particle will sink or float, but you have set what size a particle will sink or float at. You have good reason to choose this, but for this system and its specific viscosity. We know you model cannot be perfect because this critical size is the same regardless of an order of magnitude viscosity change. Yet your discussion/the plot is very centred around the size a crystal is dictates if it sinks or floats…. Which you decided…

Response: We respectfully disagree with this comment. The critical particle size for whether a crystal will flow or descend in a moving magma corresponds is the size for which its settling velocity is equal to the velocity of the ascending magma. The settling velocity is calculated following Stoke's law for a given viscosity and is not decided by us. Ascending velocities that are higher than the settling velocity defined for a given particle size by Stoke's Law will lead to an upward crystal movement, while the inverse is true for lower velocities. Changing the viscosity of the medium requires a corresponding change in magma influx velocity to sustain a crystal of the same critical particle size. In order to clarify this point, the new version of the Supplementary Material demonstrates, step by step, how the elutriation equation was deduced (see Response to **Line 343**). In addition, we have further explored how the variations in size of a critical particle and how changing the viscosity of the magma can change the velocities of the moving crystals (new **Figure 8** in the supplementary material).

Line 370-371: Please use significant digits.

Response: We have changed the text following this suggestion.

Line 395: The word necessitating suggests to me that you MUST have a small plutonic counterpart for massive eruptions. Your results suggest to me that you COULD. If you think it is a requirement to have a small plutonic counterpart, I think you need some stronger evidence.

Response: Following this suggestion, we have changed the term.

Line 413: Is the S you are taking from Figure 3? I assume yes but this should be clearly stated.

Response: The slopes considered in our computing are from the straight segments exhibited in Figure 3, as now clearly stated in this new version of the manuscript.

Line 417: changing the unit mid-sentence is confusing. Stick with ky or years.

Response: In this new version of the manuscript, we have used solely one unit to keep the sentence consistent to avoid confusing the reader.

Line 425-426: I'm not sure you can say it far exceeds. Fig 7 shows settling times of 10^2-10^5 years which overlaps with you extremely wide range of 0.2-10 ky.

Response: In this sentence, we highlight that lower growth rates result in crystallization timescales that exceed the settling times. In this new version of the manuscript, we have rewritten this sentence to make this point clearer.

Line 429 – 431: If this is true, I would expect to see some zoning??? It looks like in Fig 2 there is some with K. Maybe discuss this a bit more, or change the maps to show an element which shows this stronger???

Response: There is no observable compositional zoning in the Afs crystals in petrographic photographs, compositional maps, and cathodoluminescence images either in volcanic or plutonic samples. Compositional zoning is expected to be observable only in the volcanic record, as in the plutonic realm crystals can achieve thermodynamic equilibrium and become homogeneous. Based on our unpublished data, there is evidence for compositional zoning in quartz crystals (CL images) and thermometric data supporting this interpretation, however there is no observable internal variation of major and trace elements in Afs.

Line 486: What is (s.l.) for?

Response: The *s.l.* stands for *sensu lato*, the Latin term that mean "in a broad sense". The revised manuscript uses the long form of the expression.

Figure 8: Please write out “crystal cumulate”, “trapped melt” and “extracted melt”. You introduce a lot of short formed in this manuscript which I think makes it harder to read. Since you only use these shortforms once in this figure and you have space to write out the words, I suggest you avoid the shortforms here.

Response: We appreciate the observation, and we have changed Figure 8, as suggested.

Fig 9: - Spelling mistake on word “Compositional”

- For e) and f) you already have the words melt extracted and crystal cumulate so I don’t think it is needed to have ME and CM and it makes it a bit busy. IF you want to make it clearer maybe make the text match the colours? Or label with a } symbol?
- What is the y axis for e/f??

Response: The spelling mistake has been corrected and following the reviewer’s suggestions we have removed MEs and CMs from each item. The y axis stands for the concentration of incompatible and compatible elements, respectively. In this new version of Figure 9 such information has been improved and it is now clearer.

Line 519: Manually tracing 300-1000 crystals per sample is A TON of work!! Is there a reason why you didn’t try to segment with image J or another software? There are a lot of great and free options out there. This approach, while I’m sure has a great control on details, is also not very reproducible… Furthermore, is there a reason for only 300 for some samples? From what I understand in CSD there are larger quantities needed?

Response: We appreciate this comment, and we agree with reviewer #3 that this is a ton of work. However, this can be done with little resources, and the time investment is not too high relative to the quality of textural data and its usefulness for petrology, as demonstrated in this contribution. As compacted Afs crystals are difficult to distinguish from one another (see **Fig.2**), there is no image treatment capable of tracing each crystal outline without losing resolution, so tracing it manually still leads to better results. Additionally, each outline can be treated separately, which has facilitated the redrawing of some samples and the explicit exclusion of overgrown rims and late-formed crystals. By having the compositional maps

and the high-resolution images of samples under parallel and crossed polarized light, we are certain that anyone is able to reproduce the same figures we have traced. Furthermore, CSD requires at least ∼250 crystals, but preferably >400 crystals for a good resolution at small sizes (see Gualda, 2006). Samples with fewer crystals (~300) in our dataset are in general of volcanic origin, and their lower numbers are caused by their very low abundances. However, they still contain > 250 crystals, considering solely the Afs phenocrysts, thus passing the threshold cited above, which is reflected in the coherence between their results and other samples analyzed in our study.

REVIEWERS' COMMENTS

Reviewer #2 (Remarks to the Author):

I have carefully reviewed the revised version of the manuscript. Overall, I believe the manuscript has improved substantially in clarity and structure following the authors' revisions. The argumentation is now easier to follow, and several additional explanations introduced in the revised text help the reader better understand the methodological framework and the interpretation of the results. The introduction is well written and clearly frames the central problem addressed in the study: the difficulty of recognizing silicic cumulates based on textural evidence, particularly given the polymineralic nature of these rocks and the potential for re-equilibration textures. The authors successfully contextualize their work within the broader and still highly relevant question of volcanic–plutonic connections in felsic magmatic systems. The objective of the study (to evaluate crystal–melt segregation processes in the case study presented) is clearly stated and well justified.

In terms of impact, the most significant contribution of the manuscript lies on its discussion of crystal–melt segregation processes in silicic reservoirs. In particular, the conclusion that melt extraction may begin at lower crystallinities than predicted by classical mush models represents an important conceptual advance. The statement that "our findings suggest that melt extraction began at lower crystallinities than predicted by the classical mush model, resulting in higher melt extraction efficiency than initially computed" effectively captures the novelty and implications of the study.

The presentation of the analytical results and modeling has also improved in the revised version. I find that the additional explanatory sentences included by the authors, for instance regarding the interpretation of the R-value, are helpful for guiding the reader through the methodology. There remain a few minor points where the presentation could be further clarified (see attached pdf) so that readers do not need to revisit the text multiple times to follow certain methodological aspects, but overall the manuscript reaches a high standard consistent with what is expected for a journal such as Nature Communications.

The section introducing the dynamic mush model is also clearer in the revised version. The authors now begin by briefly describing the classical mush model and then contrast it with their results, particularly in terms of the crystallinity range over which crystal–melt separation may occur. The additional definitions introduced in the revised manuscript are welcome and improve the clarity of the text (lines 336-343).

Overall, the manuscript now presents a coherent argument showing how the proposed dynamic mush model links textural observations with broader implications for the nature and dynamics of shallow magmatic reservoirs, their role in the generation of felsic magmas, and their capacity to feed caldera-forming eruptions. In my view, the authors have addressed the comments raised in the first round of review in a satisfactory manner.

Regarding the broader significance of the work, the study provides quantitative textural evidence for crystal–melt segregation processes operating in a shallow silicic magmatic reservoir. These results have implications for understanding the formation of felsic cumulates, the internal dynamics of magma reservoirs, and their relationship with large volcanic eruptions. The quantitative analysis of crystal size distributions and related textural parameters suggests that crystal–melt separation may operate over a wider crystallinity window than predicted by classical

mush models and that dynamic processes, such as synneusis, may enhance segregation efficiency. These insights contribute to ongoing discussions about the origin of silicic magmas and the assembly of magma reservoirs capable of feeding caldera-forming eruptions.

The work is therefore likely to be of significant interest to researchers studying magmatic systems, volcanic–plutonic relationships, and magma reservoir dynamics. The integration of quantitative textural analysis with compositional modeling is particularly valuable, and the case study itself appears to be exceptionally well characterized.

The conclusions are generally supported by the data and analyses presented. I do have some reservations regarding the interpretation of certain textures as evidence of synneusis. While this interpretation is plausible and consistent with the proposed dynamic framework, such processes are inherently difficult to demonstrate unequivocally in the rock record. Similar textures could potentially arise through alternative mechanisms. For this reason, I suggest presenting this interpretation somewhat more cautiously. This point is addressed in more detail in my annotated comments in the attached PDF.

In general, I did not identify major flaws in the data analysis or interpretation. The manuscript appears complete, and the supplementary material is useful and appropriate. The remaining issues are relatively minor and mainly concern clarity of presentation and explanation in specific passages. Addressing these points would further improve the accessibility of the manuscript but does not require substantial revision. The methodology is sound and meets the standards expected in this field. The quantitative approach to textural analysis is appropriate and robust. One point that could improve reproducibility concerns the raw measurements used for the CSD and SDP analyses. Unless I overlooked them, I did not find a table including the individual crystal measurements (e.g., crystal sizes, positions within the section, and classification into size bins or families). Providing these data as a supplementary table would facilitate independent validation of the calculations and further strengthen the methodological rigor of the study.

In summary, I believe the manuscript has been substantially improved and now presents a strong and well-supported contribution to the study of crystal–melt segregation in silicic magmatic systems. The remaining comments provided in the annotated PDF are intended mainly to improve clarity and precision in the presentation.

Response: We thank Reviewer #2 for the thorough evaluation of the revised manuscript and for recognizing the substantial improvements made in response to the first-round comments. We appreciate the positive assessment of the manuscript's clarity, methodological framework, and broader implications for crystal–melt segregation in silicic magmatic systems.

We also thank the reviewer for the constructive suggestions. Following these recommendations, we have revised the text to present the interpretation of synneusis-related textures more cautiously. In addition, we have included the raw figures obtained for crystal measurement datasets, further used for the CSD and SDP analyses, and we included the results from the quantitative textural analysis in the supplementary table 1 to enhance transparency and facilitate reproducibility. We are grateful for the reviewer's thoughtful comments and encouraging evaluation of the significance and quality of our work.

Line by line comments

Lines 56-57: Regarding the broader impact of the study, I consider this to be the main novel contribution of the manuscript: the discussion on the processes of liquid–crystal segregation. This aspect is particularly important and worth emphasizing. The statement "our findings suggest that

melt extraction began at lower crystallinities than predicted by the classical mush model, resulting in higher melt extraction efficiency than initially computed” effectively highlights the significance of the results and their implications for models of melt segregation.
Response: We appreciate your comment.

Line 78: vol.% (include the units hereafter).
Response: Following this suggestion, we included the units hereafter.

Figure 1, Line 98: The crystal aggregate shown in Fig. 1e and 1f are interpreted as formed by synneusis. While such a texture could potentially form through this process, I find the interpretation not entirely convincing based on the image provided. Textural evidence for synneusis is inherently difficult to demonstrate unequivocally in thin section, and the feature illustrated could also be explained by alternative processes, such as adjacent crystal growth or a twin relationship. I therefore suggest that the interpretation as synneusis should be presented more cautiously unless additional supporting evidence is provided. Line 106: To be franc, I'm not conveinced of that. Read the comment before this one.
Response: We appreciate your concern, and, as we interpret some of the crystal agglomerates as forming only in pairs as evidence for synneusis, we make clear that this is a supposition presented with caution and based on their distribution within the sample. Furthermore, we attempt to highlight regions in which twinning can be distinguished from planes of crystal attachment.

Lines 145-146: The sentence is somewhat unclear. It is not evident which samples you mean that display the lower R-values. From the figure, it appears that the volcanic samples plot at lower R-values, but this is not clearly stated in the text (lower is relative). I suggest specifying explicitly which group shows the lower R-values to avoid ambiguity.
Response: We appreciate the comment, and it was in fact ambiguous. We were referring to the entire data set in comparison to the RSDL (Randon Sphere Distribution Line). Samples from the occurrences included in the study case are apparently constituted by Afs crystals within frameworks following the trends of spheres that limit ordered and clustered distributions. This can be associated with a relatively regular size distribution within each sample, whereas the lower R-values are associated with a relative increase in poor-sorting, as the natural samples are not constituted by same sized particles randomly distributed.
Figure 3: I suggest that the authors provide a supplementary table including the raw measurements used for the CSD and SDP calculations (e.g., crystal sizes, position within the thin section, and the classification into size bins or families). Making these data available would facilitate validation and reproducibility of the analyses and would add further rigor to the study.
Response: We appreciate the suggestion, which was a concern similar to that of Reviewer #3, and in this new version of the manuscript, we have included the results obtained in the textural analysis in more detail as a supplementary table, as well as the images from which they were derived as supplementary figures.

Lines 200-201: The sentence appears somewhat abrupt and difficult to follow in its current context. It is not entirely clear how the crystallinity-ordering patterns observed in the volcanic record specifically indicate mechanical modification through melt gain or crystal loss. Why onbnly

the volcanic record? What processes do you mean with mechanical modification? I suggest that the authors clarify this statement and briefly explain the reasoning linking the observed patterns to this interpretation.
Response: We appreciate this observation and, following it, we have improved this sentence. In our Supplementary Material 3 (Assessing igneous textures in terms of crystal-melt segregation), we have defined the terminology, but to increase accessibility and a broad understanding, we added a quick explanation of what mechanically modified textures are.

Line 209: Again, what do you mean with this term?
Response: Please, see response to Lines 200-201.

Figure 4: It might be preferable to unify the way this plot is presented throughout the manuscript, particularly between Fig. 4 and Fig. 3c. A more consistent graphical format would make comparisons between these figures clearer for the reader.
Response: Thank you for your concern about a consistent graphical format throughout the manuscript. Each diagram comes from a different reference. We believe that the use of the terms "crystallinity" and "non-crystalline volume" should be sufficient for a broader understanding of our study. The terminology was improved during the first review by changing the term “porosity” to “non-crystal volume”. Thus, we opted to keep using both diagrams despite the divergence.

Figure 4, Lines 231-232: ok, but what do you mean with associated? Do they behave in a similar way? Are these pattern in the inset what you would expect in the R-value vs Crystallinity plot? The path-i is represented by the RTT?
Response: We appreciate the observation and following it we improved the caption to make it clearer. In fact, the melt-extracted fraction and R-values are intrinsically associated, as illustrated in Figure 4. An increase in liquid loss is accompanied by an increase in R-values owing to crystal clustering, so these trends are expected to be found in the R-value vs crystallinity plots. All paths are schematically representing trends of cumulative liquid loss, so none of them are related to the RTT.

Lines 323-325: The statement is not entirely clear. It is not obvious why the relatively small volume of the Corupá Pluton would imply more efficient crystal-melt segregation at low crystallinities. In addition, it is unclear what this volume is being compared to (e.g., other plutons, the expected volume of the parental magma body, or volumes predicted by mush models). I suggest that the authors clarify the reasoning behind this inference and make the comparison more explicit. One possible interpretation is that a smaller magma body would cool more rapidly, implying that the time spent within the crystallinity window typically invoked in classical mush models would be relatively short. If this is the reasoning behind the statement, it would be helpful for the authors to make this argument more explicit. Or at least mention the cooling rates.
Response: We appreciate this observation and we have improved this section. In fact, the sentence argue that the observations are difficult to reconcile with the classical crystal mush model presented in the former paragraph. In that framework, crystal-melt segregation becomes efficient mainly at ~50-70 vol.% crystallinity, so the extraction of large volumes of crystal-poor silicic melt should leave behind proportionally large silicic cumulate bodies. The study provide

evidence that silicic cumulates formed during high-silica melt extraction in the CAC-VIPS, but the preserved Corupá Pluton (~15–30 km³) is relatively small compared with the erupted silicic sequences (~30–34 km³). We therefore infer that melt extraction must have been more efficient than predicted by the classical mush model, likely because significant crystal-melt segregation occurred at lower crystallinities, while the magma was still relatively mobile. This interpretation is consistent with a more dynamic mush scenario in which convection, crystal settling/elutriation, and other magmatic processes enhance segregation before a rigid crystal framework develops, allowing substantial melt extraction without producing an equivalently large cumulate residue.

Lines 366-367: How do you connect this with the sentence in lines 323-325 where you indicate the small volume of the pluton?
Response: As noted in the response to comments on lines 323-325, we interpret that the magmatic reservoir underwent an efficient process of crystal-melt segregation, rather than the formation of a small magmatic reservoir. The comparison between the volumes of preserved volcanic and plutonic rocks was made to discuss the lack of need for a proportionally larger crystal graveyard, which is the case using the extraction window predicted by the mush model.

Lines 451-453: This is somewhat difficult to follow in terms of the underlying argument. In particular, it is not entirely clear why enhanced crystal-melt segregation would reduce the need for large plutonic counterparts. If the intended idea is that the dynamic model, by including processes such as synneusis, allows more efficient crystal–melt separation and therefore requires smaller volumes of crystal-rich residues, it would be helpful to state this reasoning more explicitly for the reader.
Response: Based on our review, especially in the response to comments on lines 323-325, we believe that this issue has been resolved. Furthermore, the mechanical separation of crystals and melt has an intrinsic relationship with the thermodynamics of crystallization and reservoir assembling, which was the focus of this section. We interpreted a magma reservoir constituted by zones at which Afs crystals nucleated or grew at contrasting rates depending on the emplacement mode, but the timing required for it is the main limiting factor of the hydrodynamic mechanisms previously discussed. Thus, as the CSDs allow for an estimation of the crystallization times, we used them to suggest that the formation of glomerocrysts and the elutriation of the smaller crystals are mandatory for the construction of a thermally and compositionally zoned magma reservoir, especially those feeding silicic caldera-forming eruptions.

Reviewer #3 (Remarks to the Author):
Overall comments:

Thanks for your significant work to improve the explanation of the modelling and errors/limitations. I think the paper has been improved. I still have a few general comments, as well as some minor comments (below):

1) I still think the paper could be improved by stressing the interest of this paper to a broader audience. The changes you made helped but I think you can spell it out a bit more, especially considering this is a Nat Comm submission. Would your findings have any implication for other high-silica caldera forming eruptions worldwide? It would be great to see this applied to some of these systems (though that is a lot of work) or at least mention a few other systems that

would be impacted by your findings. The fact that you can get this sort of volcanism with a lower crystallinity is very interesting, but you can still stress this better, I think.

2) Thanks for the further explanations about CSD in the supplementary! I think that really strengthens your arguments. However, the issue of resolvable crystal size has still not been properly addressed (e.g. add something on Figure 3 to show there is a resolution limit). And I think it is also important to include images you did CSD on… you have a few images in the Supplementary but since this is your main dataset it is important to have this accessible and available.

Response: We thank Reviewer #3 for the positive evaluation of the revised manuscript and for the constructive suggestions.

We agree that the broader implications of our findings can be emphasized more clearly, especially because our results identify a mechanism that, if operating in other silicic reservoirs, would increase melt-extraction efficiency beyond that predicted by classical mush models. Although the plutonic counterparts of active caldera systems are rarely exposed and therefore cannot be directly evaluated using our approach, our study provides a texturally constrained example of enhanced crystal-melt segregation in a silicic reservoir.

If similar dynamic processes operate in other high-silica magma reservoirs, melt extraction may occur more efficiently and at lower crystallinities than predicted by classical mush models, with implications for the generation of eruptible magma, the interpretation of plutonic-volcanic volume relationships, and the rapid assembly of large silicic eruptions.

We appreciate the reviewer's comments regarding the CSD dataset. To better document the analytical limitations, we have added information on the minimum resolvable crystal size and the associated resolution limit of the method. In addition, we have included the images used for the CSD analyses in the Supplementary Information, ensuring that the primary dataset is fully accessible and available for evaluation and reproducibility.

We thank the reviewer for these valuable suggestions, which have helped improve the clarity, accessibility, and broader significance of the manuscript.

Line by line comments:

Line 18: I think to make this a bit more clearer I'd replace "the former" with syenite.

Line 19: Do you results indicate ONLY early melt extraction? Or also a smaller %? The smaller percentage is the thing that I found very striking and more important!

Response: We have estimated the percentage of interstitial liquid, which may include a dominant liquid fraction plus subordinate crystals, only for samples from the plutonic counterpart of the CAC-VIPS. The only example that could fit this interpretation is the granitic cupola. However, it matches geochemically the extracted melt computed using the rhyolite-MELTS algorithm, and it intersects, within errors, the Random Texture Trend, which is supposedly representative of the textural evolution of a static magma batch. We appreciate the concern, but the larger volume of extracted melt has fed a caldera-forming eruption, whereas if there was a percentage of liquid loss from the granitic cupola, it probably resulted in the silicic dikes that cross-cut the volcanic sequences later. This is an important interpretation, but it is based on the possibility of liquid loss from the granitic cupola, which was already demonstrated by the literature elsewhere. So, we

keep on focusing on the extraction of silicic liquids to give rise to caldera-forming eruptions, which is in our opinion the main finding of this contribution.

Fig 2: I think labelling the minerals in this figure, like you have done in Fig1 would be helpful. With respect to my previous comment about the scale bar – I meant the colour. You have green = K… but is dark green 10, 2 or 0.2 % Please add a compositional scale.
Response: Although the compositional maps using µ-XRF are a good tool for textural and compositional evaluation, they are merely qualitative, which means that there are no compositional scales available. So, this is why we referred to them as "relatively enriched zones" for instance, rather than interpreting in terms of composition, petrological evolution, and/or thermodynamics. In the same regard, as these maps are qualitative estimates, labeling the mineral phases can be problematic, because olivine, clinopyroxenes, and amphiboles can be indistinguishable at Fe maps.

Line 125: It is unclear to me which intercept? Again, I'm not a CSD expert so maybe I'm missing something obvious, but this is supposed to be a Nat Comm paper, so it needs to be approachable to a general audience. Also maybe ref to the figures in the sentence for clarity? (e.g. "Regular trachyte (Fig 3a red) and the medium grained granites (Fig 3b blue))
Response: We appreciate the comment, and we have improved the sentence adding the term "y-intercept" and the references to Figures 3a and 3b to make it clearer.

Line 125-126: Missing a reference (or ref your supplementary Fig 1 here).
Response: Reference and citation for our Supplementary Material 1 have been added.

Line 117-134: This section I still this is missing a discussion regarding resolution of your method. I think the kink/hump pattern you see is probably actually because of a resolution issue, which you also agree with (in part) in your rebuttal. Yes, there will be a decrease in xtal size in the late stage but you cannot know this unless you account for the resolution problem.

It looks like from your supplementary data you only did the CSD with one resolution. There is a lower limit of what size crystals you can functionally measure with any magnification, which you have one sentence about in the supplementary materials. My recommendation is that you determine what the lower limit of measurement is and plot that on Fig 3 and add at least one sentence here or in the Figure caption about the resolution limit.

I don't know if there is CSD literature which has done this (maybe Gualda 2006?) but I can point you to a nice paper by Thomas Shea (Textural studies of vesicles in volcanic rocks: An integrated methodology, 2010) which discusses this same problem but for bubbles (section 3.4 Choice of Imaging magnification).
Response: Following the reviewer's suggestion, we quantified the effective lower size limit of our measurements. Crystals smaller than 10 pixels in area were excluded, corresponding to an effective minimum measurable size of approximately 0.20 - 0.34 mm. The kink occurs at crystal sizes substantially larger than both the effective measurement threshold (~0.05 mm) and the smallest retained crystals in the dataset (~0.20 - 0.34 mm), suggesting that it is not an artifact produced by image-resolution limitations. Furthermore, image resolution does not affect the CSDs of igneous samples (see Higgins, 2006) nor outcomes of our manuscript because we have

focused on the larger crystals, and we have even excluded the smallest crystals for the corrected textural analysis.

Additionally, my comment from the previous rendition still stand ("I cannot tell which one is kinked – the lines are a bit too thick, so it is hard to see which is connected to which. Maybe consider making them thinner or changing the shade slightly (e.g. warm tones = syenites, cool = granite)? It might be helpful to indicate where you think the kink is? As it stands, I am not convinced that there is a kink.")
I would suggest you make the differences between the reds clearer… maybe I am a bit colourblind but the difference between the dark and light red is really hard for me to see. And it is impossible to tell how the same shades of red (fig 3b) follow through. I would suggest each line gets it's own colour, but keep the tones the same (e.g. med grained syenites are orange, med to very coarse syenites are a dark red, red, dark pink, light pink etc).
Response: We appreciate the observation, and we tried once again to improve this figure for a better visualization. The color coding followed the one used to distinguish volcanic from plutonic samples, and we used shades of orange and red to highlight samples exhibiting features that are different from the usual. This new version of Figure 4 and the comments about size resolution are strong arguments for a mixed crystal size population in this particular sample.

Line 186-201: With respect to your answer to "You only have one granite sample so how can you trust this is the correct value? Is there any evidence of heterogeneity in the granite samples?" in the rebuttal – this makes sense but I still think it is not properly stressed in the MS that these samples are very homogenous. I suggest you add a sentence somewhere in this paragraph that explains that! I cannot know the samples are homogenous as a non-expert of the CAC-VIPS without you telling me.
Response: We appreciate the concern of Reviewer #3, and following its guidance, we have detailed our reasoning for using only one granitic sample in the "Methods" section. We stress once again that the granitic cupola can register some minor mechanical modification, but it certainly does not influence the caldera-forming eruption registered within the CAC-VIPS.

Line 203: I find the use of the word compaction throughout the MS a little bit confusing… you redefine it at line 338 but it might be more useful to define what you mean by compaction here instead. Compaction to me make me think that there is a force (other than gravity because that would be settling) driving the crystals to compact… but not sure what this force would be (at least at this stage in the MS).
Response: In this section of the manuscript, the term is merely descriptive, indicating that crystals were clustered together irrespective of the driving mechanism. We have added a justification that we observe an increase in crystal clustering, to avoid include interpretations. In fact, the term comes form Jerram et al. (1996), but we further presents a more detailed definition at line 338. Although being critical, we kept the original trends from this classical reference in the figures and in the text, but using single quotes.

Line 212: What is the natural correction? This is the first time in the manuscript you mention it. It would be helpful for a clearer explanation of what you are defining as the natural correction.

Response: We have previously introduced the terminology to explain that it is a manual correction to separate primocrysts from crystals grown lately and supposedly before the magma reached the rheological locking.

Fig 4: I still think it would be helpful to include the Sup Fig 4 figures in this figure to better understand what you did. It is good to have a visual representation too. Since you keep stressing it is a novel approach then it is worth it to properly explain it.
Response: We appreciate the comment, and this is a novelty for quantitative textural analysis. However, our Supplementary Figure 4 represents the textural treatment we applied to our samples, and it is not a result that supports the main interpretations of our manuscript. For this reason, we have chosen to keep it as supplementary material for our contribution.

Line 270: Thanks for your explanation of the secondary ridged percolation threshold in the rebuttal!
Response: We appreciate.

Line 394: Thanks for the additional information in the Supplementary to derive this!
Response: We appreciate

Line 420: Perhaps ref to Sup Figure 8 here as well?
Response: We have added a reference to our supplementary material 5.

Line 470-472: I still think you have significant overlap in the two timescales you present here. I don't think you can say it clearly exceeds when there is 8,000 years of overlap!! Perhaps you could say the maximal times exceed?
Response: We agree that the original wording overstated the distinction between the two timescales. The lower end of the crystallization-time range (~2,000 - 10,000 years) overlaps with the upper end of the settling-time range and therefore cannot be considered clearly longer. We have revised the text to emphasize that the two timescales are comparable at their overlapping limits, whereas the maximum crystallization times exceed settling times by up to an order of magnitude.

Supplementary methods: Thanks for the additional details here! I found this to be very helpful and I think rounds the paper out a bit more for a general audience.
Response: We appreciate.

Sup part 5: is dynamic viscosity including crystals???
Response: Dynamic viscosity μ only considers the fluid, as mentioned in the Supplement to Part 5. Although an increase in the crystal fraction causes an increase in viscosity, this can be easily compared to the effects of increased viscosity, but we did not attempt to model this parameter as it is beyond the scope of our study.

Fig 8: I still don't understand how the different viscosities have the same d0? It is very non-intuitive to me… I wonder if you are accounting for the different densities you would get from a mafic v

felsic phenocryst? Perhaps it would be helpful to know what you took at the density of the crystal and melt for Fig 8b? I'm missing something here!

Response: The critical particle size d_0 is a measure of the diameter for which a given magma velocity moving upwards will keep this spherical object suspended. It does not depend on the magma viscosity, so it can be compared between magmas with contrasting viscosities. Furthermore, the densities of crystals and magmas are not a problem because the effective density depends on the contrast between the densities of solids and liquids. In the case of basalts and granites, the effective density is in the same order of magnitude, and they will have a minor influence on the difference in their settling/elutriation velocities. In this sense, the only effective variable that is considerably different between these two magmas is their viscosities.

Sup Fig 2-3 Is this all the data? I only see three samples here and you clearly have more. I cannot check you data (the CSD) if you don't include it…

Response: We appreciate the suggestion, which was a concern similar to that of Reviewer #2, and in this new version of the manuscript, we have included the results obtained in the textural analysis in more detail within our supplementary table 1, as well as the images from which they were derived as supplementary figures.

Overall minor suggestion: I found it a bit confusing that there are supplementary Fig 1 and figure 1 in this document. Maybe just combine it all (or extend the numbering system?).

Response: We have sectioned the Supplementary Material into several units, each one including "Information", "Figures", "Tables", and "Data Therefore, we believe that maintaining the current nomenclature will make it easier for readers to understand.
